# Joint analysis of lncRNA m^6^A methylome and lncRNA/mRNA expression profiles in gastric cancer

**DOI:** 10.1186/s12935-020-01554-8

**Published:** 2020-09-25

**Authors:** Zhi Lv, Liping Sun, Qian Xu, Chengzhong Xing, Yuan Yuan

**Affiliations:** 1 Tumor Etiology and Screening Department of Cancer Institute and General Surgery, The First Hospital of China Medical University, No. 155 NanjingBei Street, Heping District, Shenyang, 110001 Liaoning China; 2 Key Laboratory of Cancer Etiology and Prevention in Liaoning Education Department, The First Hospital of China Medical University, Shenyang, 110001 China; 3 Key Laboratory of GI Cancer Etiology and Prevention in Liaoning Province, the First Hospital of China Medical University, Shenyang, 110001 China

**Keywords:** LncRNA, m^6^A, Methylome profile, Expression profile, Gastric cancer

## Abstract

**Background:**

*N*^6^-methyladenosine (m^6^A) modification might be closely associated with the genesis and development of gastric cancer (GC). Currently, the evidence established by high-throughput assay for GC-related m^6^A patterns based on long non-coding RNAs (lncRNAs) remains limited. Here, a joint analysis of lncRNA m^6^A methylome and lncRNA/mRNA expression profiles in GC was performed to explore the regulatory roles of m^6^A modification in lncRNAs.

**Methods:**

Three subjects with primary GC were enrolled in our study and paired sample was randomly selected from GC tissue and adjacent normal tissue for each case. Methylated RNA Immunoprecipitation NextGeneration Sequencing (MeRIP-Seq) and Microarray Gene Expression Profiling was subsequently performed. Then co-expression analysis and gene enrichment analysis were successively conducted.

**Results:**

After data analysis, we identified 191 differentially m^6^A-methylated lncRNAs, 240 differentially expressed lncRNAs and 229 differentially expressed mRNAs in GC. Furthermore, four differentially m^6^A-methylated and expressed lncRNAs (dme-lncRNAs) were discovered including RASAL2-AS1, LINC00910, SNHG7 and LINC01105. Their potential target genes were explored by co-expression analysis. And gene enrichment analysis suggested that they might influence the cellular processes and biological behaviors involved in mitosis and cell cycle. The potential impacts of these targets on GC cells were further validated by CCLE database and literature review.

**Conclusions:**

Four novel dme-lncRNAs were identified in GC, which might exert regulatory roles on GC cell proliferation. The present study would provide clues for the lncRNA m^6^A methylation-based research on GC epigenetic etiology and pathogenesis.

## Background

*N*^6^-methyladenosine (m^6^A) is the most predominant internal chemical modification of messenger RNAs (mRNAs) in eukaryotes [1, 2]. It has been implicated in all aspects of post-transcriptional RNA metabolism, including half-life, splicing, translational efficiency, nuclear export and RNA structure [3]. The widespread nature of m^6^A in human transcriptomes has attracted a huge interest owing to technological advances in sequencing. The exploration on methylation patterns in tissue and cells could not only reveal the specific distribution of m^6^A modification in numerous transcripts, but also uncover the differences in m^6^A status under physiological and pathophysiological conditions [4]. Therefore, in-depth knowledge of m^6^A methylome profile has great benefit for elucidating the development of various human diseases especially malignancy [5, 6].

In recent years, the regulatory roles of m^6^A methylation in gastric cancer (GC) has been paid increasing attention, which is the fifth most common cancer worldwide and the third leading cause among cancer-related deaths [7]. The m^6^A modification might be closely associated with GC genesis and progression [5, 8]. Previously, several studies have investigated some m^6^A or expression patterns of m^6^A-related genes in GC. Zhang et al. evaluated the m^6^A modification patterns of GC samples based on 21 m^6^A regulators and their correlation with tumor microenvironmental (TME) cell infiltration [9]. Another research conducted by Guan et al. analyzed the expression status and determinate prognostic values of m^6^A-related genes in GC [10]. Nevertheless, all these reports were supported by complete bioinformatics methods utilizing the public data of GC patients from the Cancer Genome Atlas (TCGA) and Gene Expression Omnibus (GEO) database. To date, a dataset established by high-throughput assay for m^6^A methylome and expression profiles has been still lacking. Moreover, nearly all studies for m^6^A and GC were focused on protein-coding genes. The field of GC-related m^6^A methylation based on non-coding RNAs (ncRNAs), however, remains relatively blank.

Long non-coding RNAs (lncRNAs), generally defined as transcripts longer than 200nt, comprise the majority of ncRNAs [11]. They play critical roles in chromatin organization, transcriptional and posttranscriptional regulation [12, 13]. Similar to mRNAs, lncRNAs are also modulated by m^6^A and the levels of m^6^A residues strongly depend on the cell line, tissue type and growth condition [1, 14, 15]. According to the methylated sites located on lncRNAs, m^6^A might affect their biosynthesis, secondary structure and thus biological function for tumorigenesis [16, 17]. Several lncRNAs involved in different types of cancer were shown to simultaneously acquire dynamic m^6^A modification within their structures, such as XIST, MALAT1 and HOTAIR [18]. It has been reported that ALKBH5, a demethylase mediating methylation reversal, could promote GC invasion and metastasis by demethylating the lncRNA NEAT1. In spite of this, the evidence for m^6^A-reglulated lncRNAs associated with GC is very limited. It remains unclear what the overall patterns of lncRNA m^6^A methylation in GC are like, whether they have differences with normal status, and how they influence downstream molecules and participate in gastric carcinogenesis and progression.

In the present study, the lncRNA m^6^A methylome was established based on tissue samples to identify the differentially m^6^A-methylated lncRNAs in GC. Meanwhile, the expression profiles of lncRNA/mRNA were also designed to further explore the potential function of m^6^A-regulated lncRNAs involved in GC initiation. The study aims to provide novel clues for the disclosure of epigenetic etiology and pathogenesis of GC related to lncRNA m^6^A methylation.

## Methods

### Sample collection

The project has been approved by the ethics committee of the First Hospital of China Medical University and each participant has signed written informed consent. Three subjects with primary GC who attended the hospital seeking for surgical therapy were enrolled in our study, from January 2010 to February 2011. They were screened to have no history of other malignancies and not receive any preoperative radiotherapy or chemotherapy. After surgical resection, gastric specimens were respectively obtained from them, consisting of carcinoma tissue and adjacent cancer-free tissue. Histopathological diagnosis for GC was independently carried out by two senior gastrointestinal pathologists. The basic and pathological characteristics of the three GC cases were presented in Additional file 1: Table S1. For each case, paired sample was randomly selected from GC tissue and adjacent normal tissue, which was taken out in the distance of > 3 cm from tumor margin. Finally, all the paired fresh samples were divided into three parts, one for HE-confirmed diagnosis, another for m^6^A methylation sequencing and the other for expression profiling microarray, which were immediately frozen in liquid nitrogen and stored at − 80 °C to be detected.

### LncRNA m^6^A methylation sequencing and identification

Methylated RNA Immunoprecipitation NextGeneration Sequencing (MeRIP-Seq) was performed by Cloudseq Biotech Inc. (Shanghai, China) based on published procedures [1]. Briefly, fragmented RNA was incubated with anti-m^6^A polyclonal antibody (Synaptic Systems, 202003) in IPP buffer for 2 h at 4 °C. The mixture was then immunoprecipitated by incubation with protein-A beads (Thermo Fisher) at 4 °C for an additional 2 h. Bound RNA was eluted from the beads with m^6^A (BERRY&ASSOCIATES, PR3732) in IPP buffer and then extracted with Trizol reagent (Thermo Fisher) by following the manufacturer’s protocol. Purified RNA was used for RNA-seq library generation with NEBNext^®^ Ultra™ RNA Library Prep Kit (NEB). Both the input sample without immunoprecipitation and the m^6^A IP samples were subjected to 150 bp paired-end sequencing on Illumina HiSeq sequencer.

Paired-end reads were harvested from Illumina HiSeq 4000 sequencer, and quality control was carried out by Q30. After 3′ adaptor-trimming and the removal of low quality reads by Cutadapt software (v1.9.3) [19], clean reads of all libraries were aligned to the reference genome (UCSC HG19) by Hisat2 software (v2.0.4) [20]. Finally, methylated sites on RNAs (peaks) were identified by MACS software and annotated by homemade scripts [21].

### LncRNA/mRNA expression profiling microarray

Microarray Gene Expression Profiling was performed by Bio Miao Biological Technology (Beijing, China). The Agilent Human lncRNA Micorarry V5 (4*180 K, Design ID: 076500) was adopted in the experiment. Total RNA was quantified by NanoDrop ND-2000 (Thermo Scientific) and RNA integrity was assessed using Agilent Bioanalyzer 2100 (Agilent Technologies). Sample labeling, microarray hybridization and washing were performed according to the manufacturer’s standard instructions. Briefly, total RNA were transcribed to double strand cDNA, then synthesized into cRNA and labeled with Cyanine-3-CTP. The labeled cRNAs were hybridized onto the microarray. After washing, the arrays were scanned by Agilent Scanner G2505C (Agilent Technologies).

Feature Extraction software (v10.7.1.1) was used to analyze array images to get raw data. Genespring (v13.1) was employed to finish basic analysis with the raw data. To begin with, the raw data was normalized with quantile algorithm. The probes that at least 1 group had 100% flags in “P” were chosen for further data analysis.

### Data analysis

Differentially m^6^A-methylated lncRNA sites were identified by diffReps software [22]. Differentially expressed lncRNAs or mRNAs were identified by calculation with paired t-test. The threshold for up- or down-regulated methylation or expression was set at absolute fold change (FC) > 2.0 and *P *< 0.05. The expression correlation of differentially m^6^A-methylated and expressed lncRNAs (dme-lncRNAs) with differentially expressed mRNAs was evaluated using Pearson correlation by SPSS software (v22.0). And the co-expression network was constructed by Cytoscape software (v3.6.1). Funrich database (v3.1.3) was adopted to perform gene enrichment analysis for the differentially co-expressed genes related to dme-lncRNAs, including the prediction of expression sites, Gene Ontology (GO) enrichment and biological pathways. The normalized data for mRNA expression levels of differentially co-expressed genes in GC cell lines were downloaded from Cancer Cell Line Encyclopedia (CCLE, https://portals.broadinstitute.org/ccle). And independent t-test was used to estimate the differences of mRNA expression levels between two groups. R-project (v3.6.2) and Rstudio software (v1.2.5033) were applied to data processing and mapping. Records with |FC| > 2.0 and *P *< 0.05 (after Bonferroni correction) were considered to have statistically significant difference.

## Results

### Characteristics of lncRNA m^6^A methylome in GC

**Fig. 1:**
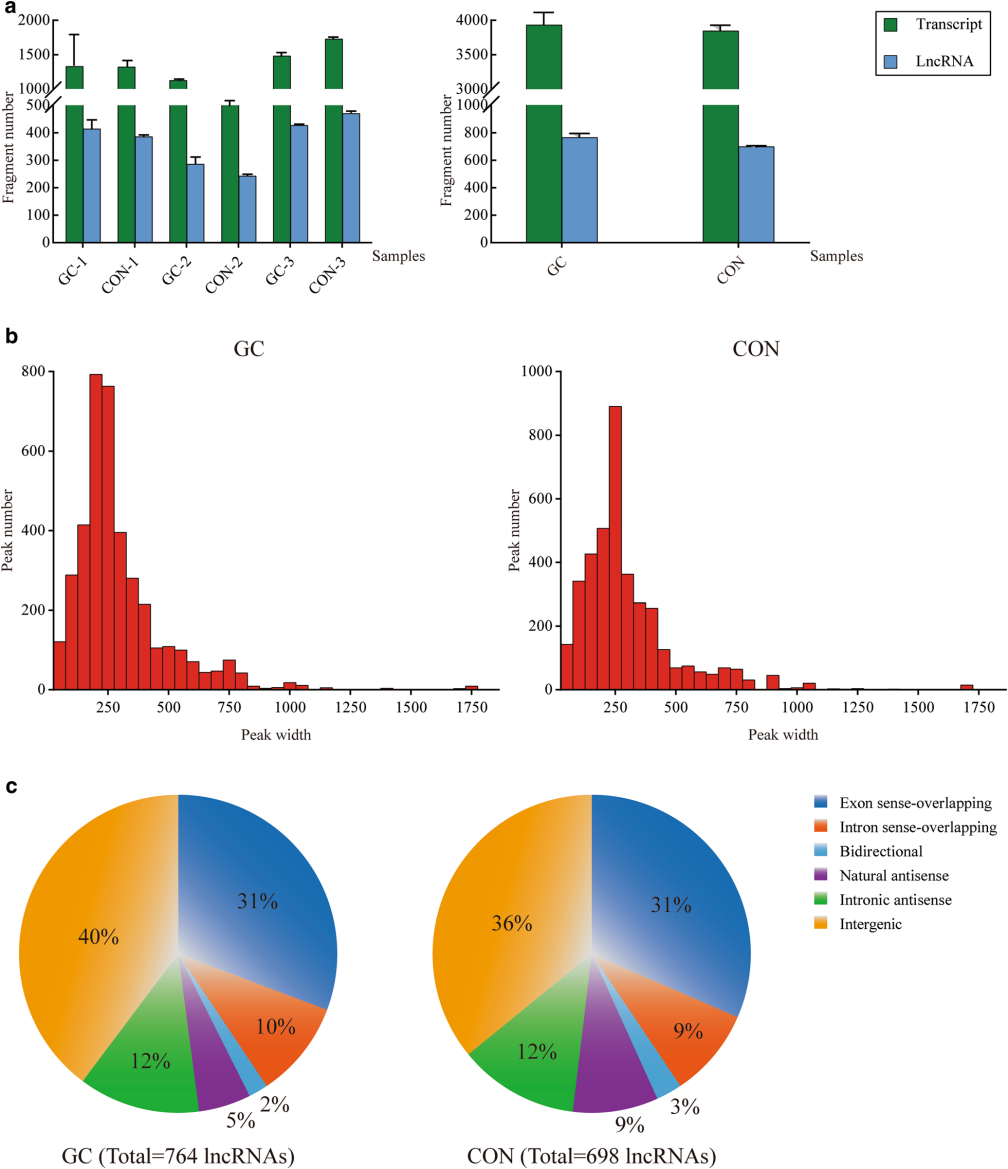
The overall features of lncRNA m^6^A methylome in GC. **a** the quantitative data of RNA transcripts in MeRIP and m^6^A-methylated lncRNAs; **b** the peak width distribution of lncRNA m^6^A-methylated sites in sequencing; **c** the source distribution of m^6^A-methylated lncRNAs

In this study, six samples were detected for lncRNA m^6^A methylation, including three GC tissue specimens (GC1-3) as the GC group and three paired normal tissue specimens (CON1-3) as the CON group. The overall features of lncRNA m^6^A methylome were shown in Fig. 1. In total, 7775 m^6^A-containing RNA transcripts were successfully enriched on lncRNA genomes for all samples. Then, 764 and 698 lncRNAs with m^6^A methylation were respectively identified in the GC and CON group (Fig. 1a). The distribution of peak width for methylated sites on lncRNAs had a similar trend between the two groups. Most lengths of m^6^A-methylated sites were concentrated in the period of 50–400 bp (Fig. 1b). A descriptive statistics was also made for the source of all m^6^A-methylated lncRNAs. It was found that intergenic lncRNAs and exon sense-overlapping lncRNAs took up the majority of them (40% and 31% in GC; 36% and 31% in CON), while bidirectional lncRNAs had the least proportion in the both groups (Fig. 1c). Furthermore, a total of 191 lncRNAs with differential m^6^A methylation were identified in GC compared with normal tissue, including 57 up-regulated lncRNAs and 134 down-regulated lncRNAs (|FC| > 2.0, *P *< 0.05, Additional file 1: Table S2).

### Characteristics of lncRNA/mRNA expression profiles in GC

**Fig. 2:**
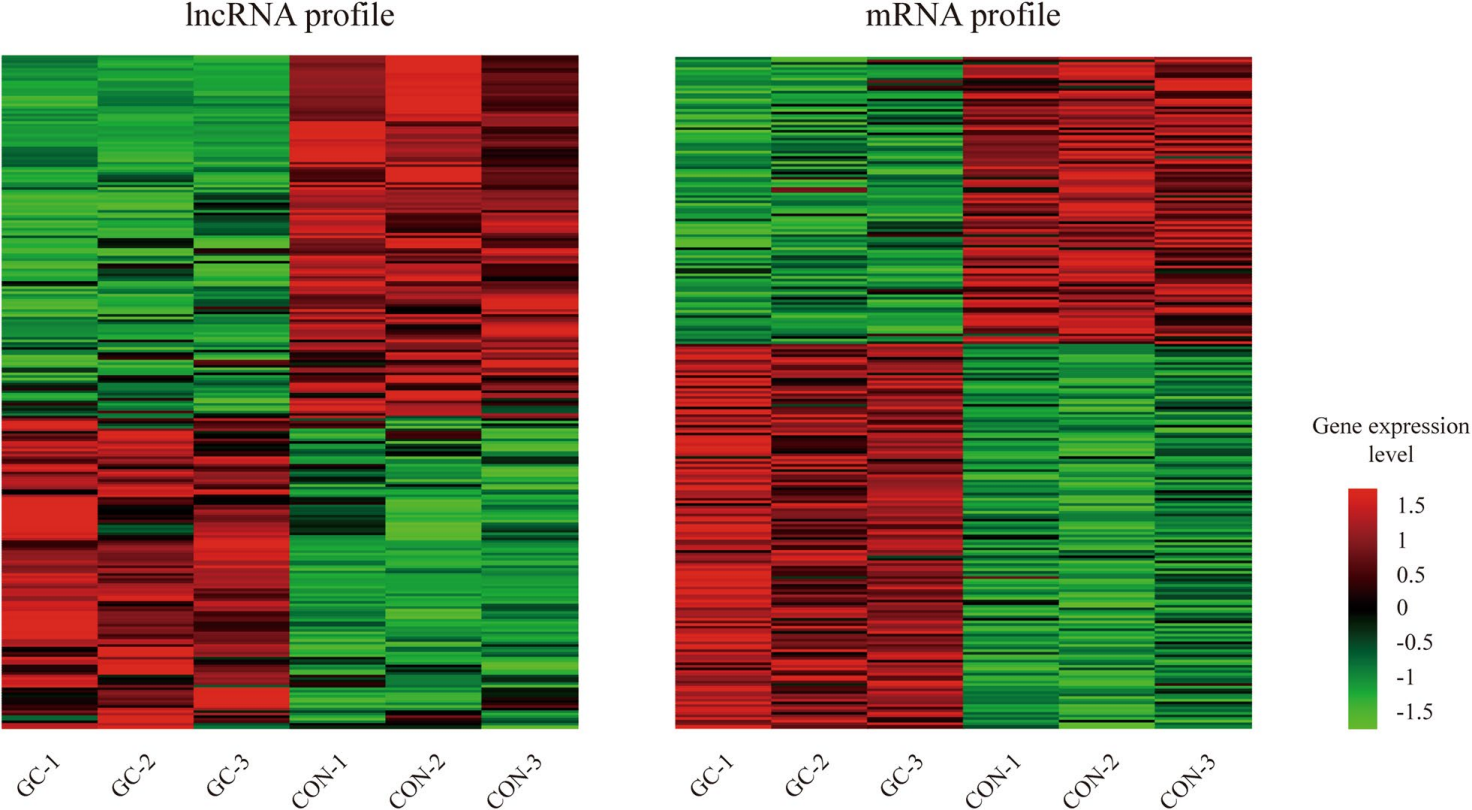
The heat map of expression levels for differentially expressed lncRNAs and mRNAs in GC

The expression patterns of lncRNA/mRNA in GC were investigated by designing three paired tissue chips of gene expression profiling. In the microarray, 89459 probes and 36143 probes were annotated to lncRNA and mRNA genomes respectively. Then we got the expression levels of 9989 lncRNAs and 9987 mRNAs with gene symbol in the six samples. Differentially expressed genes between the GC and CON group were further screened. Compared with normal tissue, 240 lncRNAs and 229 mRNAs with differential expression in GC were obtained, including 118 up-regulated lncRNAs, 122 down-regulated lncRNAs, 143 up-regulated mRNAs and 95 down-regulated mRNAs (|FC| > 2.0, *P *< 0.05). Their differential expression profiles in all the samples were shown in Fig. 2, and the details of lncRNA and mRNA expression levels were presented in Additional file 1: Tables S3 and S4.

### Selection of differentially m^6^A-methylated and expressed lncRNAs in GC

**Fig. 3:**
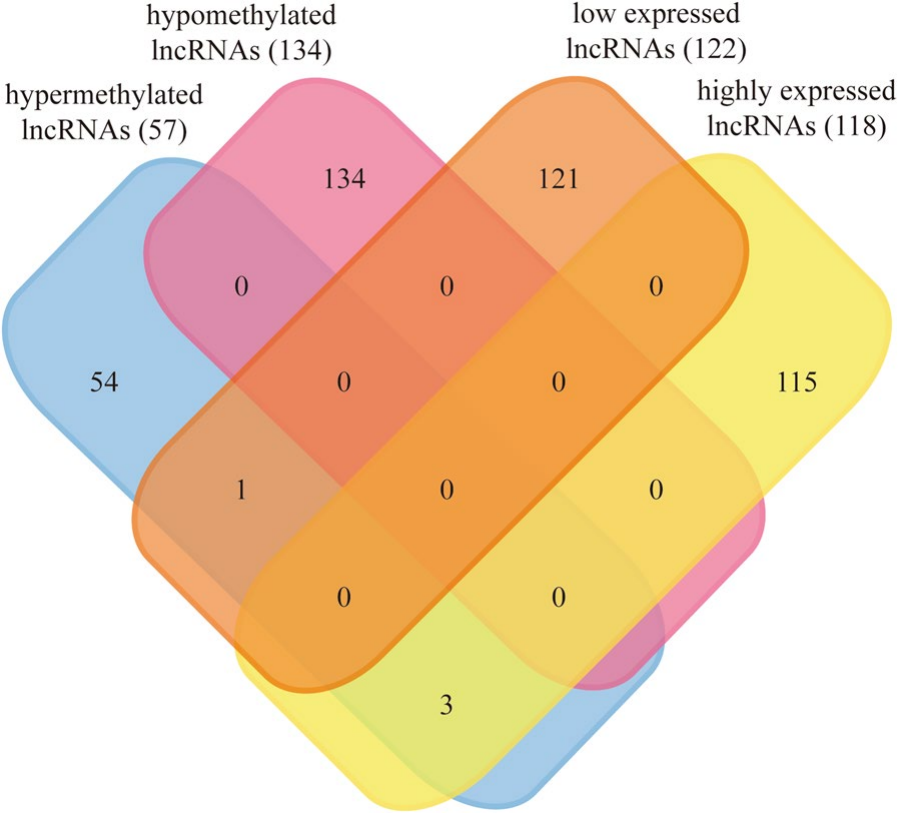
The Venn diagram of differentially m^6^A-methylated lncRNAs and differentially expressed lncRNAs in GC

Based on the findings mentioned above, a combined analysis was conducted to select the lncRNAs with both differential methylation and expression levels in GC. Thus, the 191 differentially m^6^A-methylated lncRNAs and 240 differentially expressed lncRNAs were involved. And then four dme-lncRNAs were found out, including RASAL2-AS1, LINC00910, SNHG7 and LINC01105 (Fig. 3). Among them, three lncRNAs RASAL2-AS1, LINC00910 and SNHG7 were hypermethylated and highly expressed in GC, while the other lncRNA LINC01105 was hypermethylated and low expressed in GC. Their relevant genetic information was presented in Table 1.

**Table 1:** The basic information of differentially m^6^A-methylated and expressed lncRNAs in GC

Items	RASAL2-AS1	LINC00910	SNHG7	LINC01105
Location	chr1	chr17	chr9	chr2
Length	2485	19053	3590	47531
Exon count	2	6	5	2
Relationship	Natural antisense	Intergenic	Intergenic	Exon sense overlapping
Methylated regulation	Up	Up	Up	Up
Expressed regulation	Up	Up	Up	Down

*GC* gastric cancer

### Co-expression analysis of differentially expressed mRNAs with dme-lncRNAs

**Fig. 4:**
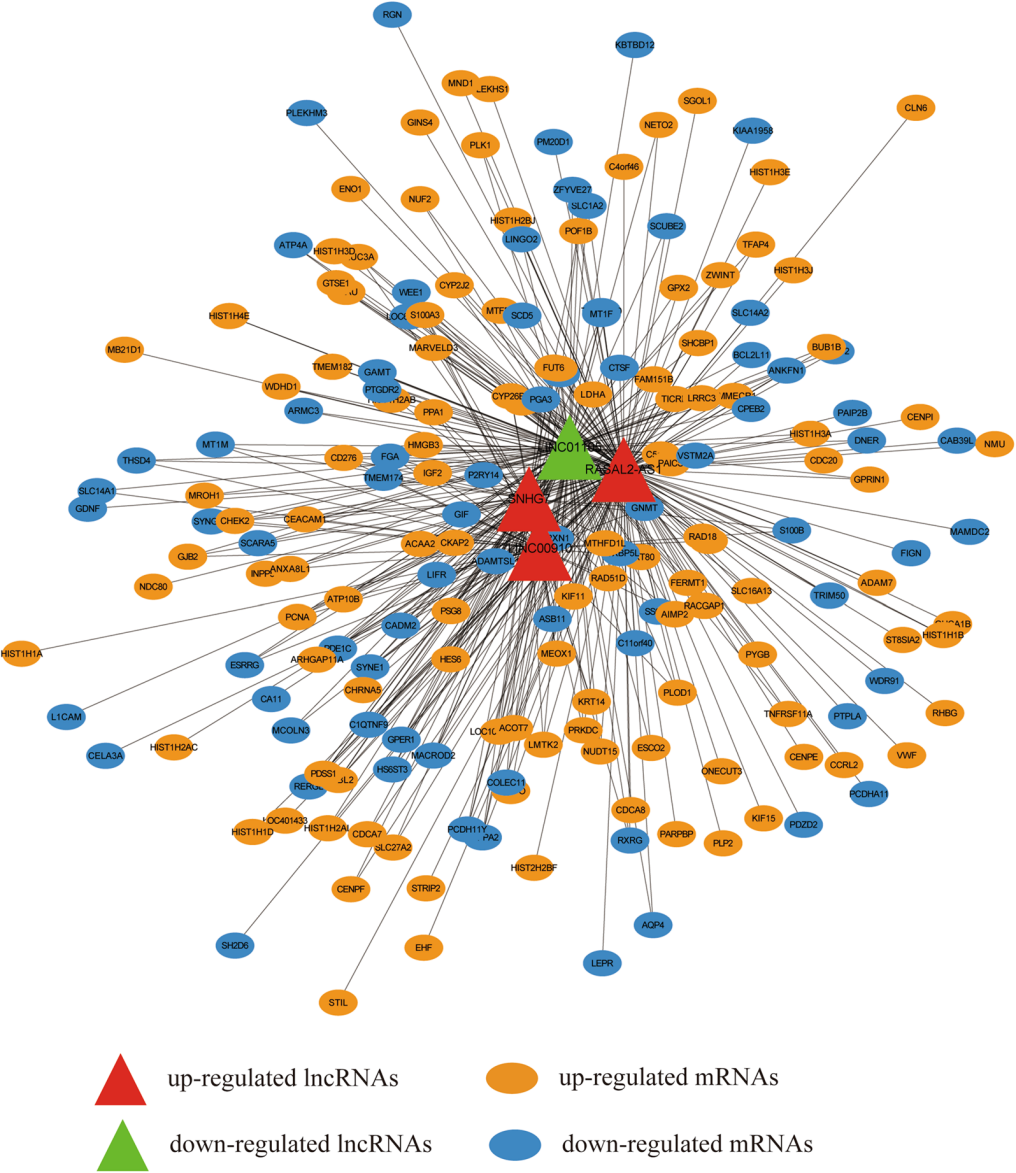
The co-expression network of dme-lncRNAs and differentially co-expressed mRNAs in GC

To explore the potential target genes of four dme-lncRNAs in GC, we analyzed the expression correlation of all differentially expressed mRNAs with them successively. The co-expressed genes of each dme-lncRNA were obtained, including 173 mRNAs for RASAL2-AS1, 54 mRNAs for LINC00910, 58 mRNAs for SNHG7 and 135 mRNAs for LINC01105. Their correlation of expression levels was shown in Additional file 1: Table S5. After integrating the four groups of genes, a co-expression network was constructed for the four dme-lncRNAs and 192 differentially co-expressed genes to visually manifest the relationship (Fig. 4). Among them, 116 genes were highly expressed and 76 genes were low expressed in GC.

### Gene enrichment analysis of differentially co-expressed genes for dme-lncRNAs

**Fig. 5:**
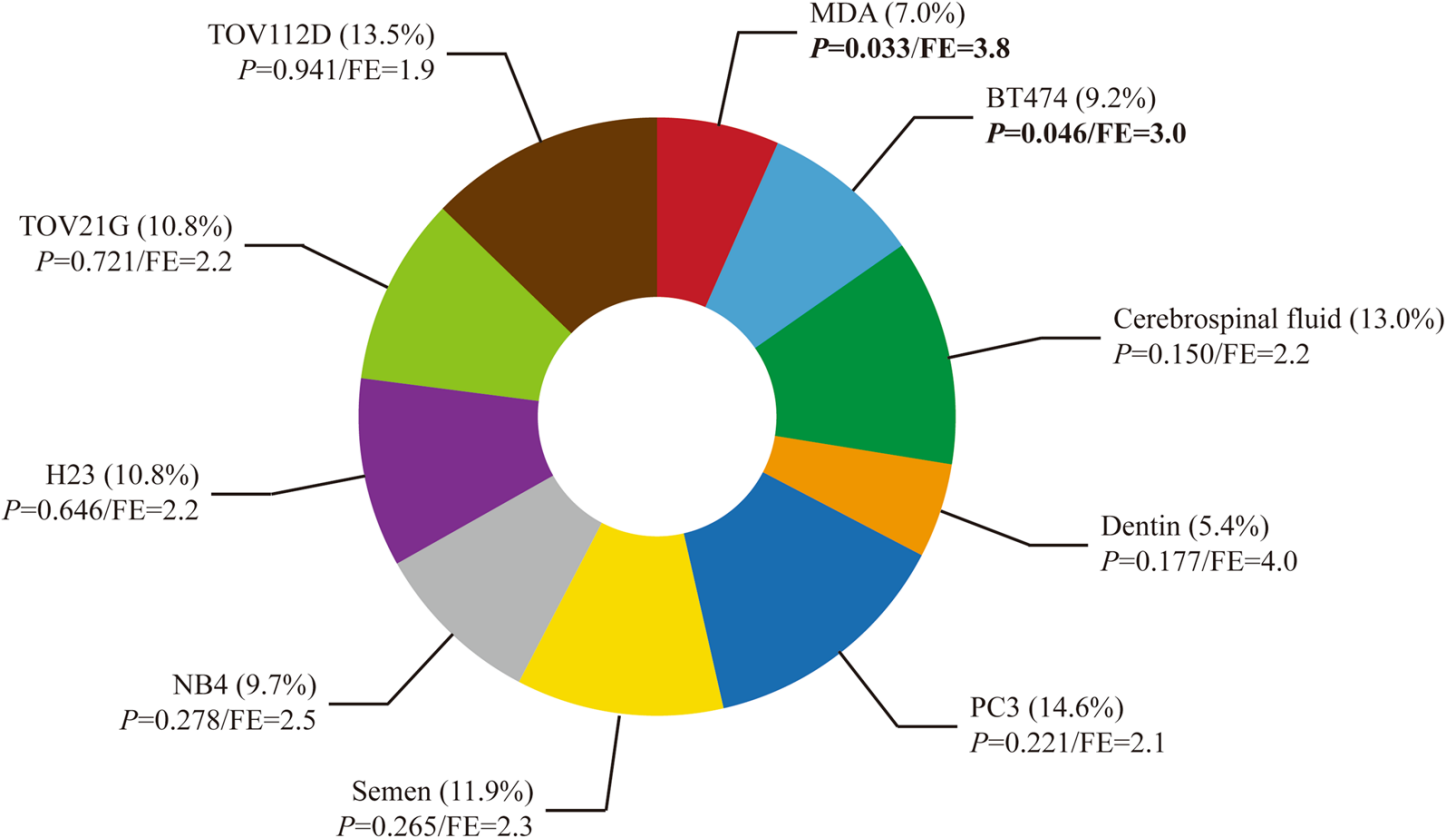
The prediction of top 10 expression sites in the enrichment analysis of differentially co-expressed genes in GC. *FE* fold enrichment

Finally, gene enrichment analysis was performed to investigate the potential biological function of differentially co-expressed genes related to dme-lncRNAs in GC. Foremost, we focused on the prediction of expression site containing a variety of normal tissues, cancer tissues, cell types and cell lines. These co-expressed genes were found to be significantly enriched in MDA and BT474, which were two types of breast cancer cell strains (*P *= 0.033 and 0.046, Fold enrichment = 3.8 and 3.0, respectively). Their percentages of enriched genes were 7.0% and 9.2% (Fig. 5).

**Fig. 6:**
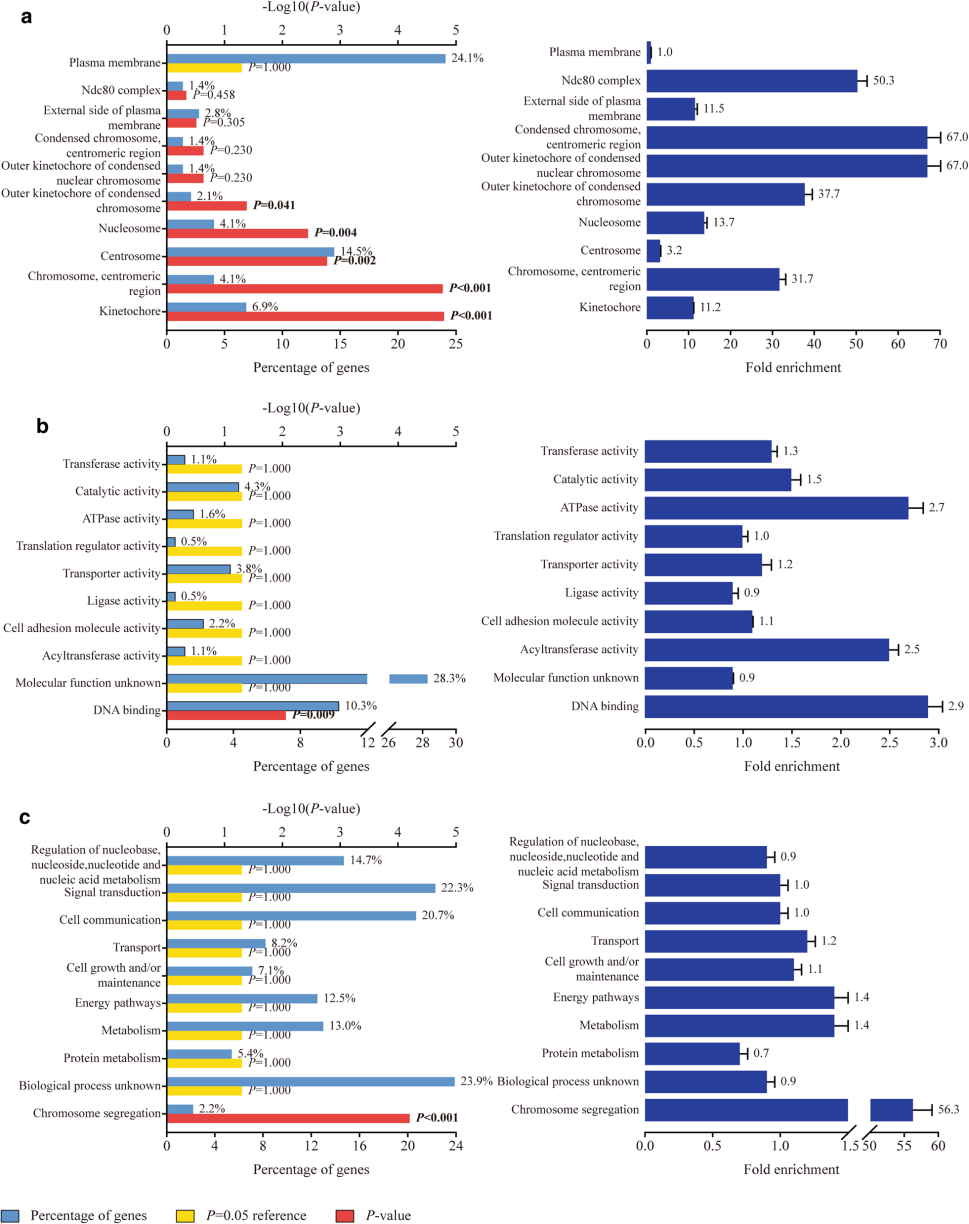
The GO-term enrichment analysis of differentially co-expressed genes in GC. Top 10 items were selected for each term. **a** cellular component; **b** molecular function; **c** biological process

GO-term enrichment analysis was next performed in regard to cellular component (CC), molecular function (MF) and biological process (BP). The top 10 items of significance were selected for each term. Concerning with CC, five items were shown to have remarkable enrichment effect of the differentially co-expressed genes, including kinetochore (*P *< 0.001), centromeric region of chromosome (*P *< 0.001), centrosome (*P *= 0.002), nucleosome (*P *= 0.004) and outer kinetochore of condensed chromosome (*P *= 0.041). All their fold enrichment was more than 2.0 (Fig. 6a). One term in MF, DNA binding, was suggested to significantly enrich those co-expressed genes (*P *= 0.009, Fold enrichment = 2.9), with a gene percentage of 10.3% (Fig. 6b). As for BP, significant enrichment effect was found in chromosome segregation (*P *< 0.001, Fold enrichment = 56.3), and its percentage of enriched genes was 2.2% (Fig. 6c).

**Fig. 7:**
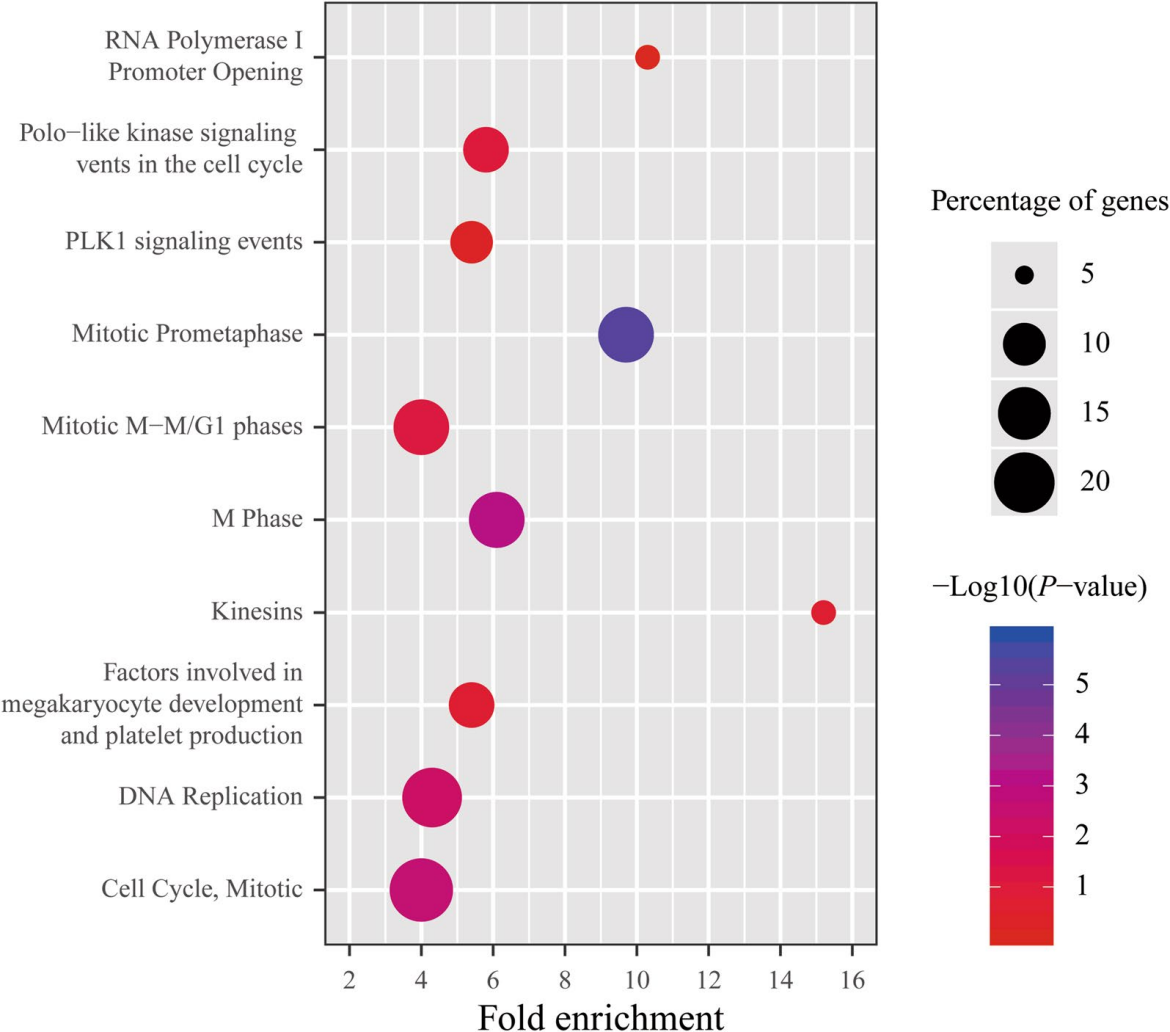
The pathway analysis of differentially co-expressed genes in GC. Top 10 items were selected

Furthermore, pathway analysis was conducted to trace the possible biological pathways where the differentially co-expressed genes could function. We also analyzed the top 10 items of significance. It was suggested that they might remarkably enriched in four biological systems including mitotic prometaphase (*P *< 0.001), M phase (*P *= 0.001), mitotic cell cycle (*P *= 0.002) and DNA replication (*P *= 0.006), with fold enrichment of 9.7, 6.1, 4.0 and 4.3. Their gene percentages of enrichment were 15.2%, 15.2%, 20.3% and 17.7%, respectively (Fig. 7).

### Validation for the potential impacts of differentially co-expressed genes on GC cells

**Fig. 8:**
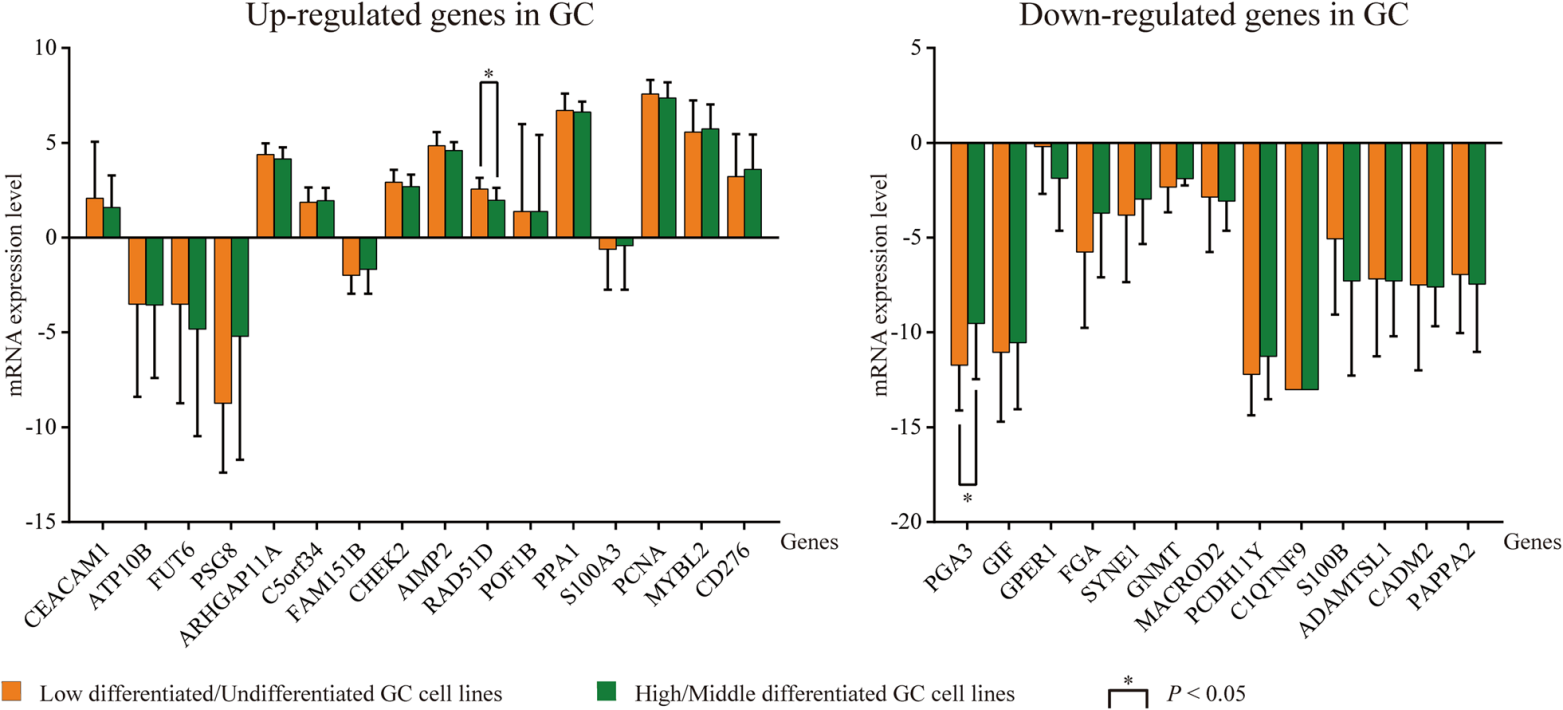
The differences in mRNA expression levels of selected differentially co-expressed genes in GC cell lines grouped by differentiation

To generally validate the regulatory roles of above targets on the biological behaviors of GC cells such as cell proliferation, we took advantage of two comprehensive approaches including an analysis in CCLE database and a literature review. Based on the previous results of co-expression analysis, 29 differentially co-expressed genes with expression correlation in all the four dme-lncRNAs were chosen as representative targets, consisting of 16 up-regulated and 13 down-regulated genes. First, the normalized data for mRNA expression levels of selected genes in 37 GC cell lines were obtained from CCLE (Additional file 1: Table S6). Then all the cell lines were classified into two groups according to their histological types, the low differentiated/undifferentiated group and the high/middle-differentiated group. Afterwards, inter-group difference in the cellular expression of selected genes was estimated respectively (Fig. 8). For up-regulated genes, their expression levels in low differentiated/undifferentiated GC cell lines were generally higher than that in the other group. Among them, the difference in RAD51D gene was statistically significant (2.55 ± 0.60 vs. 1.96 ± 0.67, *P *= 0.029). On the contrary, the down-regulated genes had normally lower expression levels in low differentiated/undifferentiated GC cell lines when compared with the other group. Markedly, the PGA3 gene showed statistical significance (-11.73 ± 2.36 vs. − 9.53 ± 2.92, *P *= 0.045).

Meanwhile, a brief review was conducted for the regulatory roles of selected differentially co-expressed genes on cancer cells (Table 2). Plenty of original studies demonstrated that the up-regulated genes could promote cell proliferation, invasion and migration or suppress cell apoptosis, thus resulting in the growth and metastasis of various tumors such as GC. By contrast, the down-regulated genes were usually involved in the inhibiting effects on cancer cell proliferation and other malignant transformation. Overall, the findings extracted from publications were relatively consistent with CCLE database.

**Table 2:** Review for the regulatory roles of selected differentially co-expressed genes on cancer cells

Genes	Chromosome	Regulation in GC tissue	Regulatory roles on cancer cells	Citation
CEACAM1	chr19	Up	Modulating GC invasiveness, lumen formation, growth and metastasis	[38, 39]
ATP10B	chr5	Up	Unknown	
FUT6	chr19	Up	Promoting CRC and BC cell proliferation, migration and invasion	[40, 41]
PSG8	chr19	Up	Unknown	
ARHGAP11A	chr15	Up	Facilitating HCC cell proliferation, invasion, migration and EMT; Promoting BLBC growth	[42–44]
C5orf34	chr5	Up	Unknown	
FAM151B	chr5	Up	Facilitating LAD cell proliferation and migration	[45]
CHEK2	chr22	Up	Promoting GC cell proliferation	[46–48]
AIMP2	chr7	Up	Suppressing cell apoptosis of lung cancer and EOC	[49–51]
RAD51D	chr17	Up	Increasing BC cell growth	[52]
POF1B	chrX	Up	Unknown	
PPA1	chr10	Up	Promoting cell proliferation and inhibiting apoptosis of NSCLC, ovarian cancer and colon cancer	[53–56]
S100A3	chr1	Up	Suppressing cell apoptosis and increasing invasion of CRPC	[57]
PCNA	chr20	Up	Cell proliferation marker	[58, 59]
MYBL2	chr20	Up	Regulating cell proliferation, survival and differentiation	[60, 61]
CD276	chr15	Up	Promoting GC cell migration and invasion	[62, 63]
PGA3	chr11	Down	Unknown	
GIF	chr11	Down	Gastric intrinsic factor	[64, 65]
GPER1	chr7	Down	Increasing GC cell apoptosis	[66]
FGA	chr4	Down	Inhibiting LAD cell proliferation, migration and invasion	[67]
SYNE1	chr6	Down	Unknown	
GNMT	chr6	Down	Promoting cell proliferation and regulating apoptosis of PC; Reducing HCC cell proliferation;	[68–70]
MACROD2	chr20	Down	Inhibiting HCC cell proliferation, invasiveness and EMT	[71]
PCDH11Y	chrY	Down	Unknown	
C1QTNF9	chr13	Down	Unknown	
S100B	chr21	Down	Inhibiting GC cell growth and invasion	[72]
ADAMTSL1	chr9	Down	Regulating chondrosarcoma cell proliferation	[73]
CADM2	chr3	Down	Inhibiting cell proliferation, migration, invasion and inducing apoptosis of various cancer	[74–79]
PAPPA2	chr1	Down	Unknown	

*GC* gastric cancer, *CRC* colorectal cancer, *BC* breast cancer, *HCC* hepatocellular carcinoma, *EMT* epithelial-to-mesenchymal transition, *BLBC* basal-like breast cancer, *LAD* lung adenocarcinoma, *EOC* epithelial ovarian cancer, *NSCLC* non-small cell lung cancer, CRPC castration-resistant prostate cancer, *PC* prostate cancer

## Discussion

LncRNA modification is a hot emerging field in cancer epigenetics with rapidly expanding interest. Here, we presented a comprehensive identification of differentially m^6^A-methylated lncRNAs in GC via MeRIP-Seq. Combined with gene expression profiling, four dme-lncRNAs were discovered including RASAL2-AS1, LINC00910, SNHG7 and LINC01105. Co-expression analysis and gene enrichment analysis were subsequently performed to explore their potential target genes and related function. Finally, the targets were selected and validated by CCLE database and literature review. To the best of our knowledge, this study firstly established the lncRNA m^6^A methylome by means of high-throughput assay and reported four dme-lncRNAs in GC. It is also the first time to illustrate the regulatory roles of differential m^6^A in lncRNAs with further impacts on the biological behaviors of GC cells.

Currently, m^6^A-centred RNA methylation has been well accepted to have close relationship with tumorigenesis including GC. For instance, a vitro experiment proved that m^6^A suppression promoted GC cell proliferation and invasiveness through activating Wnt and PI3K-Akt signaling, while m^6^A elevation reversed these phenotypical and molecular changes [8]. Another research claimed that the level of m^6^A in peripheral blood RNA was a promising noninvasive diagnostic biomarker for GC patients [5]. Therefore, the exploration for m^6^A patterns would deepen our insights into RNA posttranscriptional regulatory network participating in the complex biological processes implicated in cancer. In contrast to mRNAs, m^6^A residues in lncRNAs are distributed along the whole body of transcripts and are more concentrated in the lncRNAs undergoing alternative splicing [15]. In 2014, Batista et al. mapped m^6^A methylome in mouse and human embryonic stem cells and thousands of lncRNAs showed conserved m^6^A modification, suggesting that m^6^A was a mark of transcriptome flexibility required for stem cells to differentiate to specific lineages [23]. Moreover, Xiao et al. generated 21 whole-transcriptome m^6^A methylomes across major fetal tissues and reported that m^6^A were enriched in enhancer long intergenic non-coding RNAs (lincRNAs) [15]. Interestingly, this outcome was also indicated in our methylome study. The lincRNAs and exon-derived lncRNAs were shown to enrich m^6^A in GC, and similar trend also occurred in the 191 differentially methylated lncRNAs. It has been revealed that tissue m^6^A regions may preferentially occupy genes with single nucleotide polymorphisms (SNPs) and CpG-rich promoters, and genetic or epigenetic variation at promoters was widely associated with cancer [15, 24]. Hence, it is worth further verification whether the m^6^A modifications of differentially methylated lncRNAs in GC are regulated by these factors.

Dynamic RNA modifications are often enriched for quantitative traits and complex traits including common diseases, and thus m^6^A is potentially correlated with gene expression homeostasis [25, 26]. For instance, METTL3-mediated m^6^A modification led to LINC00958 upregulation through stabilizing its RNA transcript, and LINC00968 sponged miR-3619-5p to upregulate HDGF expression thereby facilitating the lipogenesis and progression of hepatocellular carcinoma (HCC) [27]. Another investigation revealed that m^6^A demethylase ALKBH5 could suppress the degradation of lncRNA PVT1, and its overexpression promoted osteosarcoma cell proliferation in vitro and tumor growth in vivo [28]. Similar phenomenon could also be observed in GC-related lncRNAs with m^6^A [29]. In our research, four dme-lncRNAs were newly found to have both significant m^6^A hypermethylation and differential expression levels in GC compared with normal tissue. Among them, RASAL2-AS1, SNHG7 and LINC01105 have been preliminarily studied so far, except LINC00910. RASAL2-AS1 (RASAL2 antisense RNA 1), located in chromosome 1q25.3, is a natural antisense lncRNA with 2485nt length. It has only been referred to in a bioinformatics analysis based on TCGA database for the prognostic implications of aberrantly expressed methylation-driven genes in HCC [30]. The methylation degree of RASAL2-AS1 was included in the calculation of prognostic risk score for HCC, suggesting that it could be a functional m^6^A-regulated lncRNA in carcinoma. SNHG7 (small nucleolar RNA hostgene 7) is an intergenic lncRNA located in chromosome 9q34.3 with 3590nt length, which is a novel vital oncogenic lncRNA [31]. Accumulating studies have demonstrated the association of SNHG7 with multiple human cancers via complicated mechanisms. It was found that the relative expression of SNHG7 was up-regulated in GC tissues and cells, and partially contributed to GC development and progression through regulating the expression of p15 and p16 [32]. SNHG7 was also shown to accelerate cell migration and invasion through regulating miR-34A-Snail-EMT axis in GC [33]. However, few investigations have addressed the m^6^A modification in this lncRNA yet. As for LINC01105, also named ‘SILC1’, is an exon sense overlapping lncRNA located in chromosome 2p25.2 with 47531nt length. The study for LINC01105 has been limited in its role as an oncogene of neuroblastoma. It could influence the proliferation and apoptosis of neuroblastoma cells via HIF-1alpha and p53 pathways [34, 35]. Despite the lack of direct evidence for m^6^A regulation in the four dme-lncRNAs involved in GC, our methylome and expression profiles indicated the expression of them were very likely to be regulated by m^6^A. The specific mechanisms need to be clarified by further molecular experiments.

Given that lncRNAs usually exert their regulatory roles by making effects on the expression of protein-coding genes, we identified the potential target genes for all dme-lncRNAs by analyzing their expression correlation in GC. The subsequent prediction of biological function revealed their possible association with mitosis and cell cycle. A hint could be obtained that these dme-lncRNAs might be retained and functioned in the nucleus. Nuclear lncRNAs may regulate gene expression by modulating the activity of regulatory protein complexes, chromosomal conformations and more generally, nuclear organization [36]. Our results showed the differentially co-expressed genes were mainly enriched in the following items: nucleosome, kinetochore and centrosome of CC; DNA binding of MF; chromosome segregation of BP; and DNA replication, mitotic phase and prometaphase of pathways. The cell cycle of mitosis is comprised of interphase (G1 phase, S phase and G2 phase) and mitotic phase (prophase, prometaphase, metaphase, anaphase and telophase) [37]. DNA replication occurs in S phase. After mitotic period begins, the chromatin is condensed into two chromatids connected by kinetochore and two centrosomes move towards the cell poles forming a spindle. The kinetochores are linked to centrosomes in prometaphase and then the chromatids are separated. Consequently, the potential function of those differentially co-expressed genes almost covered the biological activities in the whole stages of mitotic cell cycle, suggesting that they might be tightly associated with GC cell proliferation. Based on the above-mentioned findings, a reasonable access for lncRNA m^6^A methylation to GC could be inferred that some exogenous or endogenous factors elevated the m^6^A levels of several nuclear lncRNAs in normal cells, caused the up- or down-regulated expression of them, and then changed the expression levels of their target genes. As a result, the cell cycle and mitotic processes in which these genes may participate were affected, leading to aberrant cell proliferation and ultimate gastric carcinogenesis.

To verify the feasibility of our assumed mechanisms, the regulatory roles of screened targets on cellular biological behaviors were further explored by the aid of CCLE database and available publications. We chose 29 differentially co-expressed genes with expression correlation in all the four dme-lncRNAs as representatives comprised of 16 up-regulated and 13 down-regulated genes. Their background expression status in GC cell lines demonstrated that the up-regulated genes generally had higher expression levels in low differentiated/undifferentiated GC cell lines than high/middle-differentiated GC cell lines (RAD51D gene with significance). Meanwhile, an opposite trend was manifested in the down-regulated genes (PGA3 gene with significance). As is known to all, the tumor cells with poor differentiation usually had more malignant features like faster proliferation, stronger invasion and migration when compared with well-differentiated types. In other words, those up-regulated oncogenes might facilitate the malignant transformation of GC cells, while the down-regulated ‘tumor suppressor genes’ could inhibit or reverse their malignant phenotypes. Similar findings could also be found in published original studies. Briefly, most up-regulated genes were associated with the promotion of tumor growth, invasiveness and metastasis, while the down-regulated genes tended to suppress tumor growth and induce apoptosis. Considering the two aspects of validation results, we believed that these targets were very likely to affect the biological behaviors of GC cells such as cell proliferation, and thus indeed potential pathways mediating the m^6^A in dme-lncRNAs to exert regulatory function. Even so, the detail mechanisms for each step need exact confirmation.

It should be acknowledged that our study had a few limitations. Firstly, the sample size for detection needed to be enlarged for more accurate results. Secondly, only association study and bioinformatics analysis were focused on this topic. All the hypotheses and relevant mechanisms need to be verified by further investigations with molecular experiments. In spite of these defects, as the first report, the joint analysis of lncRNA m^6^A methylome and lncRNA/mRNA expression profiles in GC provided valuable reference for the researches in this field and also theoretical basis for future experiments.

## Conclusions

In summary, a joint analysis of lncRNA m^6^A methylome and lncRNA/mRNA expression profiles in GC was conducted to explore the regulatory roles of m^6^A modification in GC-related lncRNAs. We newly found four lncRNAs that might be modulated by m^6^A with differential expression in GC. Their potential target genes were suggested to influence the cellular processes and biological behaviors involved in mitosis and cell cycle. The potential impacts of these targets on GC cells were also supported by CCLE database and literature review. Therefore, the m^6^A levels in dme-lncRNAs might promote GC cell proliferation by regulating the expression of lncRNAs and associated genes. The present study would provide clues for the lncRNA m^6^A methylation-based research on GC epigenetic etiology and pathogenesis.

## Supplementary information

**Additional file 1.** Additional tables.

## Data Availability

All data generated and analyzed during this study are included in this published article.

## Abbreviations

m^6^A: *N*^6^-Methyladenosine
mRNA: Messenger RNA
GC: Gastric cancer
TME: Tumor microenvironmental
TCGA: The cancer genome atlas
GEO: Gene expression omnibus
ncRNA: Non-coding RNA
lncRNA: Long non-coding RNA
MeRIP-Seq: Methylated RNA Immunoprecipitation Next Generation Sequencing
FC: Fold change
dme-lncRNA: Differentially m^6^A-methylated and expressed lncRNA
GO: Gene ontology
CC: Cellular component
MF: Molecular function
BP: Biological process
CCLE: Cancer cell line encyclopedia
lincRNA: Long intergenic non-coding RNA
SNP: Single nucleotide polymorphism
HCC: Hepatocellular carcinoma
