# Supplementary material for: Joint analysis of lncRNA m6A methylome and lncRNA/mRNA expression profiles in gastric cancer
Source: Cancer Cell Int. 2020 Sep 25;20:464. doi: 10.1186/s12935-020-01554-8 (PMC7517696; doi:10.1186/s12935-020-01554-8)
Supplement: Supplementary file 1 — Additional file 1. Additional tables. [file 12935_2020_1554_MOESM1_ESM.docx]

| Table S1. The differentially m^6^A-methylated lncRNAs in GC | | | | |
| --- | --- | --- | --- | --- |
| Transcript ID | Gene name | *P* | FC(abs) | Regulation |
| NR_002768 | HYMAI | 2.40E-07 | 2.3 | up |
| ENST00000597067 | MAP1S | <1.00E-15 | 6.2 | up |
| ENST00000464316 | CCNL1 | <1.00E-15 | 10.1 | up |
| NR_002768 | HYMAI | 2.40E-07 | 2.3 | up |
| ENST00000576468 | AJ003147.9 | <1.00E-15 | 2.3 | up |
| ENST00000355638 | ADAM11 | <1.00E-15 | 16.1 | up |
| ENST00000609202 | MIR663A | <1.00E-15 | 8.9 | up |
| ENST00000523009 | TDGF1P5 | 7.61E-07 | 14.1 | up |
| ENST00000604666 | hsa-mir-335 | 2.20E-08 | 6.4 | up |
| ENST00000503050 | MAML1 | <1.00E-15 | 12.5 | up |
| NR_038368 | LINC00273 | <1.00E-15 | 2.5 | up |
| ENST00000472150 | ARHGAP21 | 5.68E-07 | 14.4 | up |
| ENST00000600807 | EMC8 | <1.00E-15 | 36.8 | up |
| ENST00000366365 | LINC00338 | <1.00E-15 | 3.3 | up |
| ENST00000523009 | TDGF1P5 | 7.61E-07 | 14.1 | up |
| ENST00000576468 | AJ003147.9 | <1.00E-15 | 2.3 | up |
| ENST00000411828 | PSMG1 | 1.49E-08 | 27.0 | up |
| ENST00000604666 | hsa-mir-335 | 2.20E-08 | 6.4 | up |
| ENST00000604666 | hsa-mir-335 | 2.20E-08 | 6.4 | up |
| ENST00000363442 | RN7SKP255 | <1.00E-15 | 5.8 | up |
| ENST00000576468 | AJ003147.9 | <1.00E-15 | 2.3 | up |
| ENST00000364768 | RNY4P16 | 2.66E-15 | 32.2 | up |
| ENST00000523009 | TDGF1P5 | 7.61E-07 | 14.1 | up |
| ENST00000419045 | AC115617.2 | <1.00E-15 | 23.0 | up |
| uc003qaq.1 | AK127472 | 5.26E-08 | 6.2 | up |
| NR_002768 | HYMAI | 2.40E-07 | 2.3 | up |
| uc002bnv.2 | LINC00925 | 1.77E-08 | 2.1 | up |
| uc003qaq.1 | AK127472 | 5.26E-08 | 6.2 | up |
| NR_033926 | ACTG1P20 | <1.00E-15 | 18.5 | up |
| ENST00000586231 | LINC00910 | 3.15E-08 | 4.8 | up |
| NR_028502 | MIR22HG | <1.00E-15 | 2.6 | up |
| NR_028502 | MIR22HG | <1.00E-15 | 2.6 | up |
| NR_002768 | HYMAI | 2.40E-07 | 2.3 | up |
| ENST00000363618 | RN7SKP203 | <1.00E-15 | 3.5 | up |
| ENST00000523009 | TDGF1P5 | 7.61E-07 | 14.1 | up |
| ENST00000563103 | CTD-2651B20.6 | <1.00E-15 | 9.9 | up |
| ENST00000609687 | MIR663A | 1.16E-09 | 5.0 | up |
| ENST00000470786 | RN7SL116P | 4.83E-10 | 3.1 | up |
| ENST00000363973 | RNY4P4 | <1.00E-15 | 11.8 | up |
| ENST00000536597 | CTD-2102P23.1 | 2.93E-06 | 8.9 | up |
| ENST00000355638 | ADAM11 | <1.00E-15 | 16.1 | up |
| ENST00000472150 | ARHGAP21 | 5.68E-07 | 14.4 | up |
| NR_024383 | LINC00461 | 2.30E-12 | 2.0 | up |
| ENST00000582078 | RP11-1113L8.6 | <1.00E-15 | 2.2 | up |
| ENST00000522856 | RP11-318M2.2 | <1.00E-15 | 6.9 | up |
| ENST00000597067 | MAP1S | <1.00E-15 | 6.2 | up |
| NR_002768 | HYMAI | 2.40E-07 | 2.3 | up |
| NR_033926 | ACTG1P20 | <1.00E-15 | 18.5 | up |
| ENST00000453434 | MTND2P24 | 8.31E-07 | 3.5 | up |
| ENST00000366365 | LINC00338 | <1.00E-15 | 3.3 | up |
| ENST00000576468 | AJ003147.9 | <1.00E-15 | 2.3 | up |
| ENST00000576468 | AJ003147.9 | <1.00E-15 | 2.3 | up |
| ENST00000604666 | hsa-mir-335 | 2.20E-08 | 6.4 | up |
| ENST00000363973 | RNY4P4 | <1.00E-15 | 11.8 | up |
| ENST00000523009 | TDGF1P5 | 7.61E-07 | 14.1 | up |
| ENST00000536597 | CTD-2102P23.1 | 2.93E-06 | 8.9 | up |
| ENST00000600807 | EMC8 | <1.00E-15 | 36.8 | up |
| NR_033926 | ACTG1P20 | <1.00E-15 | 18.5 | up |
| NR_002768 | HYMAI | 2.40E-07 | 2.3 | up |
| ENST00000470786 | RN7SL116P | 4.83E-10 | 3.1 | up |
| ENST00000601079 | MIR663A | <1.00E-15 | 4.9 | up |
| ENST00000531908 | RPS13 | 8.12E-07 | 2.6 | up |
| ENST00000419932 | CTD-2328D6.1 | <1.00E-15 | 7.4 | up |
| ENST00000604666 | hsa-mir-335 | 2.20E-08 | 6.4 | up |
| NR_002768 | HYMAI | 2.40E-07 | 2.3 | up |
| ENST00000563103 | CTD-2651B20.6 | <1.00E-15 | 9.9 | up |
| ENST00000523790 | HOXA10-AS | 5.63E-07 | 3.9 | up |
| NR_002768 | HYMAI | 2.40E-07 | 2.3 | up |
| ENST00000464316 | CCNL1 | <1.00E-15 | 10.1 | up |
| ENST00000472150 | ARHGAP21 | 5.68E-07 | 14.4 | up |
| ENST00000523009 | TDGF1P5 | 7.61E-07 | 14.1 | up |
| ENST00000576468 | AJ003147.9 | <1.00E-15 | 2.3 | up |
| ENST00000568980 | HNRNPLP2 | 7.05E-06 | 8.4 | up |
| ENST00000604666 | hsa-mir-335 | 2.20E-08 | 6.4 | up |
| ENST00000523009 | TDGF1P5 | 7.61E-07 | 14.1 | up |
| ENST00000523009 | TDGF1P5 | 7.61E-07 | 14.1 | up |
| NR_002768 | HYMAI | 2.40E-07 | 2.3 | up |
| ENST00000579003 | MIR142 | <1.00E-15 | 2.6 | up |
| ENST00000363973 | RNY4P4 | <1.00E-15 | 11.8 | up |
| ENST00000366365 | LINC00338 | <1.00E-15 | 3.3 | up |
| ENST00000576468 | AJ003147.9 | <1.00E-15 | 2.3 | up |
| ENST00000417576 | RP11-171A24.3 | 4.88E-14 | 23.6 | up |
| ENST00000503050 | MAML1 | <1.00E-15 | 12.5 | up |
| ENST00000523009 | TDGF1P5 | 7.61E-07 | 14.1 | up |
| ENST00000568314 | CTD-2651B20.7 | <1.00E-15 | 12.9 | up |
| ENST00000366365 | LINC00338 | <1.00E-15 | 3.3 | up |
| ENST00000559681 | CNOT6LP1 | 9.03E-12 | 18.6 | up |
| NR_002768 | HYMAI | 2.40E-07 | 2.3 | up |
| ENST00000597067 | MAP1S | <1.00E-15 | 6.2 | up |
| ENST00000604666 | hsa-mir-335 | 2.20E-08 | 6.4 | up |
| ENST00000461448 | SNHG12 | <1.00E-15 | 3.5 | up |
| ENST00000604666 | hsa-mir-335 | 2.20E-08 | 6.4 | up |
| ENST00000471023 | NOP56 | <1.00E-15 | 5.5 | up |
| NR_033926 | ACTG1P20 | <1.00E-15 | 18.5 | up |
| ENST00000536597 | CTD-2102P23.1 | 2.93E-06 | 8.9 | up |
| ENST00000503630 | RP11-461G12.2 | 1.64E-10 | 3.9 | up |
| ENST00000604666 | hsa-mir-335 | 2.20E-08 | 6.4 | up |
| ENST00000531908 | RPS13 | 8.12E-07 | 2.6 | up |
| ENST00000604666 | hsa-mir-335 | 2.20E-08 | 6.4 | up |
| ENST00000411828 | PSMG1 | 1.49E-08 | 27.0 | up |
| NR_002768 | HYMAI | 2.40E-07 | 2.3 | up |
| ENST00000568314 | CTD-2651B20.7 | <1.00E-15 | 12.9 | up |
| ENST00000604666 | hsa-mir-335 | 2.20E-08 | 6.4 | up |
| NR_028502 | MIR22HG | <1.00E-15 | 2.6 | up |
| ENST00000582320 | MIR451B | <1.00E-15 | 25.0 | up |
| ENST00000503630 | RP11-461G12.2 | 1.64E-10 | 3.9 | up |
| ENST00000472150 | ARHGAP21 | 5.68E-07 | 14.4 | up |
| NR_028502 | MIR22HG | <1.00E-15 | 2.6 | up |
| ENST00000536597 | CTD-2102P23.1 | 2.93E-06 | 8.9 | up |
| ENST00000425653 | DANCR | 5.33E-15 | 2.6 | up |
| ENST00000363973 | RNY4P4 | <1.00E-15 | 11.8 | up |
| ENST00000531908 | RPS13 | 8.12E-07 | 2.6 | up |
| ENST00000523009 | TDGF1P5 | 7.61E-07 | 14.1 | up |
| ENST00000457532 | AC004987.9 | 4.18E-07 | 15.2 | up |
| ENST00000597067 | MAP1S | <1.00E-15 | 6.2 | up |
| ENST00000580118 | ULK2 | <1.00E-15 | 2.8 | up |
| ENST00000503630 | RP11-461G12.2 | 1.64E-10 | 3.9 | up |
| NR_002768 | HYMAI | 2.40E-07 | 2.3 | up |
| NR_002768 | HYMAI | 2.40E-07 | 2.3 | up |
| ENST00000530207 | FNBP4 | <1.00E-15 | 6.2 | up |
| ENST00000568314 | CTD-2651B20.7 | <1.00E-15 | 12.9 | up |
| NR_002768 | HYMAI | 2.40E-07 | 2.3 | up |
| NR_002768 | HYMAI | 2.40E-07 | 2.3 | up |
| NR_002768 | HYMAI | 2.40E-07 | 2.3 | up |
| ENST00000597067 | MAP1S | <1.00E-15 | 6.2 | up |
| ENST00000503050 | MAML1 | <1.00E-15 | 12.5 | up |
| ENST00000604666 | hsa-mir-335 | 2.20E-08 | 6.4 | up |
| ENST00000491883 | CENPVP2 | 1.35E-10 | 9.5 | up |
| NR_002768 | HYMAI | 2.40E-07 | 2.3 | up |
| NR_028502 | MIR22HG | <1.00E-15 | 2.6 | up |
| ENST00000503630 | RP11-461G12.2 | 1.64E-10 | 3.9 | up |
| ENST00000364228 | RNY1 | <1.00E-15 | 4.3 | up |
| uc001vea.1 | MIR16-1 | <1.00E-15 | 2.0 | up |
| ENST00000604666 | hsa-mir-335 | 2.20E-08 | 6.4 | up |
| NR_002768 | HYMAI | 2.40E-07 | 2.3 | up |
| ENST00000445125 | AC010970.2 | <1.00E-15 | 3.3 | up |
| ENST00000604666 | hsa-mir-335 | 2.20E-08 | 6.4 | up |
| NR_002768 | HYMAI | 2.40E-07 | 2.3 | up |
| NR_077058 | AHCTF1P1 | 2.74E-11 | 7.3 | up |
| ENST00000580118 | ULK2 | <1.00E-15 | 2.8 | up |
| NR_038368 | LINC00273 | 1.45E-08 | 4.7 | up |
| NR_002768 | HYMAI | 2.40E-07 | 2.3 | up |
| ENST00000576468 | AJ003147.9 | <1.00E-15 | 2.3 | up |
| NR_003672 | SNHG7 | <1.00E-15 | 5.2 | up |
| ENST00000540175 | RP11-424C20.2 | <1.00E-15 | 2.1 | up |
| ENST00000471023 | NOP56 | <1.00E-15 | 5.5 | up |
| ENST00000563103 | CTD-2651B20.6 | <1.00E-15 | 9.9 | up |
| NR_002768 | HYMAI | 2.40E-07 | 2.3 | up |
| ENST00000604666 | hsa-mir-335 | 2.20E-08 | 6.4 | up |
| ENST00000411828 | PSMG1 | 1.49E-08 | 27.0 | up |
| ENST00000577700 | SNHG15 | 6.90E-05 | 2.2 | up |
| NR_003672 | SNHG7 | <1.00E-15 | 5.2 | up |
| NR_026832 | LINC01105 | 5.36E-05 | 2.2 | up |
| NR_027982 | RASAL2-AS1 | 1.35E-02 | 7.7 | up |
| NR_027982 | RASAL2-AS1 | 1.35E-02 | 7.7 | up |
| ENST00000487309 | RN7SL397P | <1.00E-15 | 2.1 | down |
| ENST00000568314 | CTD-2651B20.7 | 1.89E-13 | 9.4 | down |
| ENST00000494487 | RN7SL691P | 3.79E-11 | 106.1 | down |
| ENST00000489392 | RPL7A | <1.00E-15 | 13.9 | down |
| ENST00000463999 | RN7SL515P | 1.00E-06 | 15.3 | down |
| ENST00000563749 | RPL13 | <1.00E-15 | 35.5 | down |
| ENST00000482732 | RPL10 | 4.96E-07 | 2.8 | down |
| uc001yxa.1 | SNURF | <1.00E-15 | 290.8 | down |
| ENST00000584058 | RN7SL4P | 3.35E-14 | 21.9 | down |
| ENST00000578824 | ACADVL | <1.00E-15 | 9.1 | down |
| ENST00000584901 | EIF4A1 | <1.00E-15 | 2.8 | down |
| ENST00000511037 | MTND5P11 | 2.98E-12 | 2.8 | down |
| ENST00000584901 | EIF4A1 | <1.00E-15 | 2.8 | down |
| NR_027033 | MIRLET7BHG | <1.00E-15 | 3.3 | down |
| ENST00000497899 | ENAH | 1.11E-08 | 6.5 | down |
| ENST00000563749 | RPL13 | <1.00E-15 | 35.5 | down |
| ENST00000563749 | RPL13 | <1.00E-15 | 35.5 | down |
| ENST00000416931 | MTND1P23 | <1.00E-15 | 11.5 | down |
| ENST00000579495 | RPL17 | <1.00E-15 | 13.2 | down |
| ENST00000483264 | CCAR1 | <1.00E-15 | 7.6 | down |
| ENST00000580921 | MED24 | <1.00E-15 | 47.0 | down |
| ENST00000421068 | GAS5 | <1.00E-15 | 7.8 | down |
| ENST00000547169 | CTDSP2 | <1.00E-15 | 3.1 | down |
| ENST00000579495 | RPL17 | <1.00E-15 | 13.2 | down |
| ENST00000579085 | EIF4A1 | 3.91E-09 | 5.9 | down |
| ENST00000584058 | RN7SL4P | 3.35E-14 | 21.9 | down |
| ENST00000419932 | CTD-2328D6.1 | <1.00E-15 | 2.2 | down |
| ENST00000482732 | RPL10 | 4.96E-07 | 2.8 | down |
| ENST00000481456 | DDX39B | <1.00E-15 | 16.6 | down |
| ENST00000476268 | RPL13A | <1.00E-15 | 26.3 | down |
| ENST00000485752 | RNF149 | <1.00E-15 | 16.1 | down |
| ENST00000527871 | RPS2 | <1.00E-15 | 2.2 | down |
| ENST00000481456 | DDX39B | <1.00E-15 | 16.6 | down |
| ENST00000579495 | RPL17 | <1.00E-15 | 13.2 | down |
| ENST00000579085 | EIF4A1 | 3.91E-09 | 5.9 | down |
| ENST00000582718 | AC010761.8 | <1.00E-15 | 43.8 | down |
| ENST00000514057 | MTATP6P1 | <1.00E-15 | 4.3 | down |
| ENST00000485390 | RPS8 | <1.00E-15 | 36.3 | down |
| ENST00000587743 | SNHG16 | <1.00E-15 | 15.8 | down |
| ENST00000431268 | GAS5 | <1.00E-15 | 14.5 | down |
| ENST00000513060 | GNB2L1 | <1.00E-15 | 21.1 | down |
| ENST00000566491 | RPL4 | <1.00E-15 | 36.7 | down |
| ENST00000481456 | DDX39B | <1.00E-15 | 16.6 | down |
| ENST00000580645 | CTD-2006O16.2 | <1.00E-15 | 10.5 | down |
| ENST00000563103 | CTD-2651B20.6 | 2.08E-06 | 10.1 | down |
| ENST00000602573 | SNHG8 | 4.62E-10 | 2.8 | down |
| ENST00000422990 | MTND2P23 | 1.28E-07 | 68.1 | down |
| ENST00000473668 | RN7SL364P | 7.31E-11 | 3.5 | down |
| uc001yxa.1 | SNURF | <1.00E-15 | 290.8 | down |
| ENST00000481456 | DDX39B | <1.00E-15 | 16.6 | down |
| ENST00000513060 | GNB2L1 | <1.00E-15 | 21.1 | down |
| ENST00000584901 | EIF4A1 | <1.00E-15 | 2.8 | down |
| ENST00000520612 | MTND6P3 | <1.00E-15 | 6.0 | down |
| ENST00000580921 | MED24 | <1.00E-15 | 47.0 | down |
| ENST00000513060 | GNB2L1 | <1.00E-15 | 21.1 | down |
| ENST00000431268 | GAS5 | <1.00E-15 | 14.5 | down |
| ENST00000577438 | RP11-846F4.5 | <1.00E-15 | 11.0 | down |
| ENST00000527871 | RPS2 | <1.00E-15 | 2.2 | down |
| ENST00000590268 | PPP1R12C | 3.41E-10 | 9.8 | down |
| ENST00000496254 | GNL3 | <1.00E-15 | 15.8 | down |
| ENST00000584901 | EIF4A1 | <1.00E-15 | 2.8 | down |
| ENST00000553465 | MEG8 | <1.00E-15 | 16.0 | down |
| ENST00000565181 | RP11-1007O24.3 | 3.21E-09 | 20.9 | down |
| ENST00000483264 | CCAR1 | <1.00E-15 | 7.6 | down |
| NR_110178 | SNHG24 | <1.00E-15 | 32.4 | down |
| ENST00000513060 | GNB2L1 | <1.00E-15 | 21.1 | down |
| ENST00000489392 | RPL7A | <1.00E-15 | 13.9 | down |
| ENST00000485101 | EIF4A2 | <1.00E-15 | 21.3 | down |
| ENST00000574371 | RP11-750B16.1 | 8.26E-08 | 3.4 | down |
| ENST00000579085 | EIF4A1 | 3.91E-09 | 5.9 | down |
| ENST00000364663 | RN7SKP237 | 2.33E-06 | 54.6 | down |
| ENST00000460537 | RN7SL151P | 2.02E-11 | 8.3 | down |
| ENST00000563749 | RPL13 | <1.00E-15 | 35.5 | down |
| ENST00000498589 | PPIL2 | 3.11E-15 | 3.3 | down |
| ENST00000567229 | RPL4 | <1.00E-15 | 5.7 | down |
| ENST00000580755 | RPL23A | <1.00E-15 | 21.7 | down |
| ENST00000485101 | EIF4A2 | <1.00E-15 | 21.3 | down |
| ENST00000476268 | RPL13A | <1.00E-15 | 26.3 | down |
| ENST00000485101 | EIF4A2 | <1.00E-15 | 21.3 | down |
| ENST00000484022 | GNL3 | <1.00E-15 | 8.6 | down |
| ENST00000579085 | EIF4A1 | 3.91E-09 | 5.9 | down |
| uc001yxa.1 | SNURF | <1.00E-15 | 290.8 | down |
| ENST00000572453 | MIR497HG | <1.00E-15 | 4.4 | down |
| NR_024127 | SNHG12 | <1.00E-15 | 19.9 | down |
| ENST00000591956 | SNHG16 | 8.16E-08 | 2.3 | down |
| ENST00000530585 | RPL27A | <1.00E-15 | 2.4 | down |
| ENST00000514318 | GNB2L1 | <1.00E-15 | 23.4 | down |
| NR_110835 | KCTD2 | 1.15E-09 | 3.9 | down |
| ENST00000600238 | RPL18A | <1.00E-15 | 6.7 | down |
| ENST00000481456 | DDX39B | <1.00E-15 | 16.6 | down |
| ENST00000582718 | AC010761.8 | <1.00E-15 | 43.8 | down |
| ENST00000433442 | RP11-162J8.3 | <1.00E-15 | 18.9 | down |
| ENST00000498589 | PPIL2 | 3.11E-15 | 3.3 | down |
| ENST00000481456 | DDX39B | <1.00E-15 | 16.6 | down |
| ENST00000595794 | C19orf48 | <1.00E-15 | 15.3 | down |
| ENST00000482732 | RPL10 | 4.96E-07 | 2.8 | down |
| ENST00000547169 | CTDSP2 | <1.00E-15 | 3.1 | down |
| ENST00000485706 | RPL7A | <1.00E-15 | 8.8 | down |
| uc001yxa.1 | SNURF | <1.00E-15 | 290.8 | down |
| ENST00000594728 | METTL12 | <1.00E-15 | 10.7 | down |
| ENST00000484599 | RPS8 | <1.00E-15 | 3.5 | down |
| ENST00000485706 | RPL7A | <1.00E-15 | 8.8 | down |
| uc004avg.4 | MIR23B | <1.00E-15 | 4.6 | down |
| ENST00000471759 | RABGGTB | <1.00E-15 | 3.4 | down |
| ENST00000481456 | DDX39B | <1.00E-15 | 16.6 | down |
| ENST00000566491 | RPL4 | <1.00E-15 | 36.7 | down |
| ENST00000514318 | GNB2L1 | <1.00E-15 | 23.4 | down |
| ENST00000485101 | EIF4A2 | <1.00E-15 | 21.3 | down |
| ENST00000481456 | DDX39B | <1.00E-15 | 16.6 | down |
| ENST00000579085 | EIF4A1 | 3.91E-09 | 5.9 | down |
| ENST00000579495 | RPL17 | <1.00E-15 | 13.2 | down |
| ENST00000582718 | AC010761.8 | <1.00E-15 | 43.8 | down |
| ENST00000478941 | NOP58 | <1.00E-15 | 29.3 | down |
| ENST00000365328 | RN7SK | <1.00E-15 | 2.1 | down |
| NR_132114 | SNHG19 | <1.00E-15 | 13.7 | down |
| ENST00000563749 | RPL13 | <1.00E-15 | 35.5 | down |
| ENST00000566491 | RPL4 | <1.00E-15 | 36.7 | down |
| ENST00000456812 | GAS5 | <1.00E-15 | 14.2 | down |
| ENST00000568314 | CTD-2651B20.7 | 1.89E-13 | 9.4 | down |
| ENST00000490496 | RN7SL288P | 1.40E-09 | 89.2 | down |
| ENST00000513060 | GNB2L1 | <1.00E-15 | 21.1 | down |
| ENST00000575234 | ZNF75A | <1.00E-15 | 4.0 | down |
| ENST00000401578 | RP11-296E7.1 | 6.52E-09 | 82.0 | down |
| ENST00000488398 | RN7SL521P | 2.91E-12 | 3.5 | down |
| ENST00000545109 | PGA5 | <1.00E-15 | 32.7 | down |
| ENST00000527871 | RPS2 | <1.00E-15 | 2.2 | down |
| ENST00000445125 | AC010970.2 | <1.00E-15 | 2.5 | down |
| ENST00000576468 | AJ003147.9 | 2.35E-08 | 2.2 | down |
| uc001yxa.1 | SNURF | <1.00E-15 | 290.8 | down |
| ENST00000484599 | RPS8 | <1.00E-15 | 3.5 | down |
| ENST00000484022 | GNL3 | <1.00E-15 | 8.6 | down |
| ENST00000496254 | GNL3 | <1.00E-15 | 15.8 | down |
| ENST00000481456 | DDX39B | <1.00E-15 | 16.6 | down |
| ENST00000497519 | RPL5 | <1.00E-15 | 11.8 | down |
| NR_110835 | KCTD2 | 1.15E-09 | 3.9 | down |
| ENST00000576468 | AJ003147.9 | 2.35E-08 | 2.2 | down |
| ENST00000580755 | RPL23A | <1.00E-15 | 21.7 | down |
| ENST00000607097 | MIR378D2 | 1.80E-07 | 3.9 | down |
| ENST00000585237 | RN7SL296P | 2.39E-12 | 8.8 | down |
| ENST00000421068 | GAS5 | <1.00E-15 | 19.9 | down |
| ENST00000572453 | MIR497HG | <1.00E-15 | 4.4 | down |
| ENST00000568314 | CTD-2651B20.7 | 1.89E-13 | 9.4 | down |
| ENST00000473668 | RN7SL364P | 7.31E-11 | 3.5 | down |
| ENST00000451607 | GAS5 | <1.00E-15 | 19.0 | down |
| ENST00000478473 | C9orf3 | <1.00E-15 | 2.6 | down |
| ENST00000579495 | RPL17 | <1.00E-15 | 13.2 | down |
| ENST00000520612 | MTND6P3 | <1.00E-15 | 6.0 | down |
| ENST00000575234 | ZNF75A | <1.00E-15 | 4.0 | down |
| uc001yxa.1 | SNURF | <1.00E-15 | 290.8 | down |
| ENST00000584901 | EIF4A1 | <1.00E-15 | 2.8 | down |
| ENST00000422990 | MTND2P23 | 1.28E-07 | 68.1 | down |
| ENST00000481456 | DDX39B | <1.00E-15 | 16.6 | down |
| NR_003604 | ZFAS1 | <1.00E-15 | 15.3 | down |
| ENST00000580921 | MED24 | <1.00E-15 | 47.0 | down |
| ENST00000535568 | PGA5 | <1.00E-15 | 74.1 | down |
| ENST00000579495 | RPL17 | <1.00E-15 | 13.2 | down |
| ENST00000579495 | RPL17 | <1.00E-15 | 13.2 | down |
| ENST00000414273 | hsa-mir-6723 | 2.04E-14 | 4.0 | down |
| ENST00000473668 | RN7SL364P | 7.31E-11 | 3.5 | down |
| ENST00000435212 | MTATP8P2 | <1.00E-15 | 5.6 | down |
| ENST00000483264 | CCAR1 | <1.00E-15 | 7.6 | down |
| ENST00000600238 | RPL18A | <1.00E-15 | 6.7 | down |
| ENST00000421068 | GAS5 | <1.00E-15 | 7.8 | down |
| NR_110835 | KCTD2 | 1.15E-09 | 3.9 | down |
| ENST00000485101 | EIF4A2 | <1.00E-15 | 21.3 | down |
| ENST00000461653 | RABGGTB | <1.00E-15 | 7.7 | down |
| ENST00000435212 | MTATP8P2 | <1.00E-15 | 5.6 | down |
| ENST00000514318 | GNB2L1 | <1.00E-15 | 23.4 | down |
| ENST00000451607 | GAS5 | <1.00E-15 | 19.0 | down |
| ENST00000496254 | GNL3 | <1.00E-15 | 15.8 | down |
| ENST00000536269 | PGA5 | <1.00E-15 | 38.1 | down |
| ENST00000579003 | MIR142 | 5.68E-10 | 7.9 | down |
| ENST00000489408 | ZNF860 | <1.00E-15 | 7.9 | down |
| ENST00000484022 | GNL3 | <1.00E-15 | 8.6 | down |
| NR_038397 | DNM3OS | <1.00E-15 | 8.3 | down |
| ENST00000594728 | METTL12 | <1.00E-15 | 10.7 | down |
| ENST00000582718 | AC010761.8 | <1.00E-15 | 43.8 | down |
| ENST00000488398 | RN7SL521P | 2.91E-12 | 3.5 | down |
| ENST00000481456 | DDX39B | <1.00E-15 | 16.6 | down |
| ENST00000514318 | GNB2L1 | <1.00E-15 | 23.4 | down |
| ENST00000451607 | GAS5 | <1.00E-15 | 19.0 | down |
| ENST00000565907 | RBMX | <1.00E-15 | 18.4 | down |
| ENST00000579495 | RPL17 | <1.00E-15 | 13.2 | down |
| ENST00000538654 | SNHG1 | <1.00E-15 | 21.6 | down |
| ENST00000496254 | GNL3 | <1.00E-15 | 15.8 | down |
| ENST00000482732 | RPL10 | 4.96E-07 | 2.8 | down |
| ENST00000580645 | CTD-2006O16.2 | <1.00E-15 | 10.5 | down |
| NR_038397 | DNM3OS | <1.00E-15 | 8.3 | down |
| ENST00000513060 | GNB2L1 | <1.00E-15 | 21.1 | down |
| ENST00000421068 | GAS5 | <1.00E-15 | 7.8 | down |
| ENST00000514318 | GNB2L1 | <1.00E-15 | 23.4 | down |
| ENST00000485101 | EIF4A2 | <1.00E-15 | 21.3 | down |
| ENST00000579085 | EIF4A1 | 3.91E-09 | 5.9 | down |
| ENST00000582718 | AC010761.8 | <1.00E-15 | 43.8 | down |
| NR_047022 | LINC00434 | 4.46E-10 | 3.0 | down |
| ENST00000579085 | EIF4A1 | 3.91E-09 | 5.9 | down |
| uc001hgr.2 | mir-29b-2 | <1.00E-15 | 5.5 | down |
| ENST00000527871 | RPS2 | <1.00E-15 | 2.2 | down |
| NR_045196 | SNHG18 | <1.00E-15 | 6.3 | down |
| ENST00000527871 | RPS2 | <1.00E-15 | 2.2 | down |
| ENST00000584901 | EIF4A1 | <1.00E-15 | 2.8 | down |
| ENST00000530585 | RPL27A | <1.00E-15 | 2.4 | down |
| ENST00000476268 | RPL13A | <1.00E-15 | 26.3 | down |
| NR_038368 | LINC00273 | <1.00E-15 | 2.4 | down |
| ENST00000490496 | RN7SL288P | 1.40E-09 | 89.2 | down |
| ENST00000580645 | CTD-2006O16.2 | <1.00E-15 | 10.5 | down |
| NR_038397 | DNM3OS | <1.00E-15 | 8.3 | down |
| uc001yxh.1 | SNURF-SNRPN | <1.00E-15 | 18.1 | down |
| ENST00000467883 | RN7SL128P | <1.00E-15 | 7.7 | down |
| ENST00000538654 | SNHG1 | <1.00E-15 | 21.6 | down |
| NR_027033 | MIRLET7BHG | <1.00E-15 | 8.3 | down |
| ENST00000364663 | RN7SKP237 | 2.33E-06 | 54.6 | down |
| uc004coq.4 | DQ582201 | <1.00E-15 | 9.2 | down |
| ENST00000594728 | METTL12 | <1.00E-15 | 10.7 | down |
| ENST00000580645 | CTD-2006O16.2 | <1.00E-15 | 10.5 | down |
| ENST00000572453 | MIR497HG | <1.00E-15 | 4.4 | down |
| ENST00000463999 | RN7SL515P | 1.00E-06 | 15.3 | down |
| ENST00000595794 | C19orf48 | <1.00E-15 | 15.3 | down |
| ENST00000578824 | ACADVL | <1.00E-15 | 9.1 | down |
| uc004avg.4 | MIR23B | <1.00E-15 | 4.6 | down |
| ENST00000471023 | NOP56 | <1.00E-15 | 20.4 | down |
| ENST00000595794 | C19orf48 | <1.00E-15 | 15.3 | down |
| ENST00000530585 | RPL27A | <1.00E-15 | 2.4 | down |
| ENST00000604619 | RP11-475J5.5 | 2.16E-06 | 4.9 | down |
| ENST00000514318 | GNB2L1 | <1.00E-15 | 23.4 | down |
| ENST00000481456 | DDX39B | <1.00E-15 | 16.6 | down |
| ENST00000467525 | FCGR2A | 1.01E-10 | 3.2 | down |
| ENST00000530585 | RPL27A | <1.00E-15 | 2.4 | down |
| ENST00000421068 | GAS5 | <1.00E-15 | 19.9 | down |
| ENST00000584901 | EIF4A1 | <1.00E-15 | 2.8 | down |
| ENST00000582718 | AC010761.8 | <1.00E-15 | 43.8 | down |
| ENST00000590268 | PPP1R12C | 3.41E-10 | 9.8 | down |
| ENST00000482732 | RPL10 | 4.96E-07 | 2.8 | down |
| ENST00000467525 | FCGR2A | 1.01E-10 | 3.2 | down |
| ENST00000363804 | RN7SKP160 | 6.05E-06 | 6.1 | down |
| ENST00000578824 | ACADVL | <1.00E-15 | 9.1 | down |
| ENST00000579085 | EIF4A1 | 3.91E-09 | 5.9 | down |
| ENST00000579495 | RPL17 | <1.00E-15 | 13.2 | down |
| ENST00000584901 | EIF4A1 | <1.00E-15 | 2.8 | down |
| ENST00000580755 | RPL23A | <1.00E-15 | 21.7 | down |
| NR_023358 | SCARNA9L | <1.00E-15 | 7.0 | down |
| ENST00000538654 | SNHG1 | <1.00E-15 | 20.7 | down |
| ENST00000498589 | PPIL2 | 3.11E-15 | 3.3 | down |
| ENST00000513060 | GNB2L1 | <1.00E-15 | 21.1 | down |
| ENST00000584901 | EIF4A1 | <1.00E-15 | 2.8 | down |
| ENST00000591956 | SNHG16 | 2.17E-13 | 2.7 | down |
| ENST00000364663 | RN7SKP237 | 2.33E-06 | 54.6 | down |
| ENST00000478473 | C9orf3 | <1.00E-15 | 2.6 | down |
| ENST00000461653 | RABGGTB | <1.00E-15 | 7.7 | down |
| ENST00000431268 | GAS5 | <1.00E-15 | 14.5 | down |
| ENST00000584901 | EIF4A1 | <1.00E-15 | 2.8 | down |
| ENST00000563749 | RPL13 | <1.00E-15 | 35.5 | down |
| ENST00000498589 | PPIL2 | 3.11E-15 | 3.3 | down |
| ENST00000580755 | RPL23A | <1.00E-15 | 21.7 | down |
| ENST00000590268 | PPP1R12C | 3.41E-10 | 9.8 | down |
| ENST00000422990 | MTND2P23 | 1.28E-07 | 68.1 | down |
| ENST00000538654 | SNHG1 | <1.00E-15 | 21.6 | down |
| ENST00000478473 | C9orf3 | <1.00E-15 | 2.6 | down |
| ENST00000497519 | RPL5 | <1.00E-15 | 11.8 | down |
| ENST00000584901 | EIF4A1 | <1.00E-15 | 2.8 | down |
| ENST00000513060 | GNB2L1 | <1.00E-15 | 21.1 | down |
| ENST00000600238 | RPL18A | <1.00E-15 | 6.7 | down |
| ENST00000517961 | AC084082.3 | 6.14E-13 | 4.8 | down |
| ENST00000483264 | CCAR1 | <1.00E-15 | 7.6 | down |
| ENST00000482732 | RPL10 | 4.96E-07 | 2.8 | down |
| ENST00000579495 | RPL17 | <1.00E-15 | 13.2 | down |
| uc001yxa.1 | SNURF | <1.00E-15 | 290.8 | down |
| ENST00000481456 | DDX39B | <1.00E-15 | 16.6 | down |
| ENST00000595794 | C19orf48 | <1.00E-15 | 15.3 | down |
| ENST00000527871 | RPS2 | <1.00E-15 | 2.2 | down |
| ENST00000584058 | RN7SL4P | 3.35E-14 | 21.9 | down |
| ENST00000490496 | RN7SL288P | 1.40E-09 | 89.2 | down |
| ENST00000545109 | PGA5 | <1.00E-15 | 32.7 | down |
| ENST00000497774 | FAM211A-AS1 | <1.00E-15 | 20.8 | down |
| ENST00000590268 | PPP1R12C | 3.41E-10 | 9.8 | down |
| ENST00000498589 | PPIL2 | 3.11E-15 | 3.3 | down |
| NR_038397 | DNM3OS | <1.00E-15 | 8.3 | down |
| ENST00000451607 | GAS5 | <1.00E-15 | 19.0 | down |
| ENST00000489408 | ZNF860 | <1.00E-15 | 7.9 | down |
| ENST00000578824 | ACADVL | <1.00E-15 | 9.1 | down |
| ENST00000421068 | GAS5 | <1.00E-15 | 7.8 | down |
| NR_034179 | LOC653712 | 1.80E-07 | 6.3 | down |
| ENST00000421068 | GAS5 | <1.00E-15 | 19.9 | down |
| ENST00000530585 | RPL27A | <1.00E-15 | 2.4 | down |
| ENST00000584901 | EIF4A1 | <1.00E-15 | 2.8 | down |
| ENST00000580921 | MED24 | <1.00E-15 | 47.0 | down |
| ENST00000482732 | RPL10 | 4.96E-07 | 2.8 | down |
| ENST00000451607 | GAS5 | <1.00E-15 | 19.0 | down |
| ENST00000464971 | RN7SL308P | <1.00E-15 | 17.3 | down |
| ENST00000527871 | RPS2 | <1.00E-15 | 2.2 | down |
| ENST00000474814 | SNHG12 | <1.00E-15 | 7.8 | down |
| uc001yxa.1 | SNURF | <1.00E-15 | 290.8 | down |
| ENST00000580645 | CTD-2006O16.2 | <1.00E-15 | 10.5 | down |
| uc001hgr.2 | mir-29b-2 | 7.38E-11 | 2.7 | down |
| ENST00000580755 | RPL23A | <1.00E-15 | 21.7 | down |
| ENST00000580921 | MED24 | <1.00E-15 | 47.0 | down |
| ENST00000595794 | C19orf48 | <1.00E-15 | 15.3 | down |
| ENST00000459853 | RN7SL587P | 6.21E-13 | 8.0 | down |
| ENST00000580755 | RPL23A | <1.00E-15 | 21.7 | down |
| ENST00000513060 | GNB2L1 | <1.00E-15 | 21.1 | down |
| ENST00000565907 | RBMX | <1.00E-15 | 18.4 | down |
| NR_022008 | PWAR5 | <1.00E-15 | 40.1 | down |
| ENST00000600238 | RPL18A | <1.00E-15 | 6.7 | down |
| ENST00000607097 | MIR378D2 | 1.80E-07 | 3.9 | down |
| ENST00000421068 | GAS5 | <1.00E-15 | 7.8 | down |
| ENST00000477096 | ZRANB2 | <1.00E-15 | 3.5 | down |
| ENST00000565907 | RBMX | <1.00E-15 | 18.4 | down |
| ENST00000489392 | RPL7A | <1.00E-15 | 13.9 | down |
| ENST00000567229 | RPL4 | <1.00E-15 | 5.7 | down |
| ENST00000563749 | RPL13 | <1.00E-15 | 35.5 | down |
| ENST00000482732 | RPL10 | 4.96E-07 | 2.8 | down |
| ENST00000457890 | AC063976.3 | <1.00E-15 | 8.2 | down |
| ENST00000530564 | EIF4G2 | 1.65E-09 | 2.9 | down |
| ENST00000579495 | RPL17 | <1.00E-15 | 13.2 | down |
| ENST00000457540 | MTND2P28 | <1.00E-15 | 8.5 | down |
| ENST00000602461 | RP6-99M1.2 | <1.00E-15 | 2.3 | down |
| ENST00000595794 | C19orf48 | <1.00E-15 | 15.3 | down |
| ENST00000595794 | C19orf48 | <1.00E-15 | 15.3 | down |
| ENST00000576468 | AJ003147.9 | 2.35E-08 | 2.2 | down |
| ENST00000481456 | DDX39B | <1.00E-15 | 16.6 | down |
| ENST00000565907 | RBMX | <1.00E-15 | 18.4 | down |
| ENST00000474582 | RPS8 | <1.00E-15 | 12.5 | down |
| ENST00000584058 | RN7SL4P | 3.35E-14 | 21.9 | down |
| ENST00000478473 | C9orf3 | <1.00E-15 | 2.6 | down |
| ENST00000422990 | MTND2P23 | 1.28E-07 | 68.1 | down |
| ENST00000473668 | RN7SL364P | 7.31E-11 | 3.5 | down |
| ENST00000579495 | RPL17 | <1.00E-15 | 13.2 | down |
| ENST00000479428 | RN7SL546P | <1.00E-15 | 6.5 | down |
| ENST00000527871 | RPS2 | <1.00E-15 | 2.2 | down |
| ENST00000496254 | GNL3 | <1.00E-15 | 15.8 | down |
| ENST00000601517 | RP11-665N17.4 | <1.00E-15 | 3.5 | down |
| ENST00000513060 | GNB2L1 | <1.00E-15 | 21.1 | down |
| ENST00000579085 | EIF4A1 | 3.91E-09 | 5.9 | down |
| NR_027033 | MIRLET7BHG | <1.00E-15 | 3.3 | down |
| ENST00000467525 | FCGR2A | 1.01E-10 | 3.2 | down |
| ENST00000565336 | RP4-561L24.3 | <1.00E-15 | 5.7 | down |
| NR_108100 | SPACA6P-AS | <1.00E-15 | 12.6 | down |
| ENST00000584901 | EIF4A1 | <1.00E-15 | 2.8 | down |
| ENST00000364663 | RN7SKP237 | 2.33E-06 | 54.6 | down |
| ENST00000497899 | ENAH | 1.11E-08 | 6.5 | down |
| ENST00000547169 | CTDSP2 | <1.00E-15 | 3.1 | down |
| uc001yxa.1 | SNURF | <1.00E-15 | 290.8 | down |
| ENST00000547169 | CTDSP2 | <1.00E-15 | 3.1 | down |
| ENST00000594728 | METTL12 | <1.00E-15 | 10.7 | down |
| ENST00000497519 | RPL5 | <1.00E-15 | 11.8 | down |
| ENST00000546204 | TCP1 | <1.00E-15 | 5.6 | down |
| ENST00000602301 | VTRNA2-1 | <1.00E-15 | 13.6 | down |
| ENST00000538654 | SNHG1 | <1.00E-15 | 20.7 | down |
| ENST00000530564 | EIF4G2 | 1.65E-09 | 2.9 | down |
| ENST00000496055 | RABGGTB | <1.00E-15 | 4.3 | down |
| ENST00000564647 | RPL4 | <1.00E-15 | 6.7 | down |
| ENST00000546204 | TCP1 | <1.00E-15 | 5.6 | down |
| ENST00000483264 | CCAR1 | <1.00E-15 | 7.6 | down |
| ENST00000576762 | AJ003147.8 | <1.00E-15 | 3.2 | down |
| ENST00000514318 | GNB2L1 | <1.00E-15 | 23.4 | down |
| ENST00000467525 | FCGR2A | <1.00E-15 | 8.2 | down |
| ENST00000563749 | RPL13 | <1.00E-15 | 35.5 | down |
| ENST00000496055 | RABGGTB | <1.00E-15 | 4.3 | down |
| ENST00000514318 | GNB2L1 | <1.00E-15 | 23.4 | down |
| ENST00000467883 | RN7SL128P | <1.00E-15 | 7.7 | down |
| uc001yxa.1 | SNURF | <1.00E-15 | 290.8 | down |
| ENST00000547169 | CTDSP2 | <1.00E-15 | 3.1 | down |
| ENST00000482732 | RPL10 | 4.96E-07 | 2.8 | down |
| ENST00000567229 | RPL4 | <1.00E-15 | 5.7 | down |
| ENST00000572453 | MIR497HG | <1.00E-15 | 4.4 | down |
| ENST00000530564 | EIF4G2 | 1.65E-09 | 2.9 | down |
| ENST00000595794 | C19orf48 | <1.00E-15 | 15.3 | down |
| ENST00000579495 | RPL17 | <1.00E-15 | 13.2 | down |
| ENST00000470475 | RPS8 | <1.00E-15 | 9.7 | down |
| uc004cos.5 | TVAS5 | <1.00E-15 | 12.9 | down |
| ENST00000576468 | AJ003147.9 | 2.35E-08 | 2.2 | down |
| ENST00000564647 | RPL4 | <1.00E-15 | 6.7 | down |
| ENST00000576468 | AJ003147.9 | 2.35E-08 | 2.2 | down |
| ENST00000530585 | RPL27A | <1.00E-15 | 2.4 | down |
| ENST00000471023 | NOP56 | <1.00E-15 | 20.4 | down |
| NR_023358 | SCARNA9L | <1.00E-15 | 7.0 | down |
| ENST00000538654 | SNHG1 | <1.00E-15 | 20.7 | down |
| ENST00000523929 | HSPA9 | <1.00E-15 | 29.9 | down |
| ENST00000481456 | DDX39B | <1.00E-15 | 16.6 | down |
| ENST00000564647 | RPL4 | <1.00E-15 | 6.7 | down |
| ENST00000546204 | TCP1 | <1.00E-15 | 5.6 | down |
| ENST00000514318 | GNB2L1 | <1.00E-15 | 23.4 | down |
| ENST00000513060 | GNB2L1 | <1.00E-15 | 21.1 | down |
| ENST00000481456 | DDX39B | <1.00E-15 | 16.6 | down |
| uc001yxa.1 | SNURF | <1.00E-15 | 290.8 | down |
| uc021prp.1 | HI650153 | <1.00E-15 | 15.2 | down |
| ENST00000563103 | CTD-2651B20.6 | 2.08E-06 | 10.1 | down |
| ENST00000530585 | RPL27A | <1.00E-15 | 2.4 | down |
| ENST00000513060 | GNB2L1 | <1.00E-15 | 21.1 | down |
| ENST00000431043 | SNHG5 | <1.00E-15 | 12.0 | down |
| ENST00000567229 | RPL4 | <1.00E-15 | 5.7 | down |
| ENST00000473668 | RN7SL364P | 7.31E-11 | 3.5 | down |
| ENST00000579085 | EIF4A1 | 3.91E-09 | 5.9 | down |
| ENST00000603719 | MTND5P10 | <1.00E-15 | 13.7 | down |
| ENST00000595794 | C19orf48 | <1.00E-15 | 15.3 | down |
| ENST00000365328 | RN7SK | <1.00E-15 | 2.1 | down |
| ENST00000483264 | CCAR1 | <1.00E-15 | 7.6 | down |
| ENST00000482732 | RPL10 | 4.96E-07 | 2.8 | down |
| ENST00000471759 | RABGGTB | <1.00E-15 | 3.4 | down |
| ENST00000431268 | GAS5 | <1.00E-15 | 14.5 | down |
| ENST00000513060 | GNB2L1 | <1.00E-15 | 21.1 | down |
| ENST00000530564 | EIF4G2 | 1.65E-09 | 2.9 | down |
| ENST00000520612 | MTND6P3 | <1.00E-15 | 6.0 | down |
| ENST00000564647 | RPL4 | <1.00E-15 | 6.7 | down |
| ENST00000595794 | C19orf48 | <1.00E-15 | 15.3 | down |
| ENST00000422990 | MTND2P23 | 1.28E-07 | 68.1 | down |
| NR_110063 | LOC101927901 | <1.00E-15 | 8.5 | down |
| ENST00000530564 | EIF4G2 | 1.65E-09 | 2.9 | down |
| ENST00000580755 | RPL23A | <1.00E-15 | 21.7 | down |
| ENST00000459853 | RN7SL587P | 6.21E-13 | 8.0 | down |
| ENST00000575234 | ZNF75A | <1.00E-15 | 4.0 | down |
| ENST00000498999 | MTND4P12 | <1.00E-15 | 3.3 | down |
| NR_033947 | LIMD1-AS1 | <1.00E-15 | 2.5 | down |
| ENST00000515438 | RP11-474J18.1 | 2.85E-06 | 5.0 | down |
| ENST00000441633 | RP11-10B2.1 | 8.77E-08 | 4.2 | down |
| ENST00000431268 | GAS5 | <1.00E-15 | 14.5 | down |
| ENST00000484022 | GNL3 | <1.00E-15 | 8.6 | down |
| ENST00000514318 | GNB2L1 | <1.00E-15 | 23.4 | down |
| ENST00000460249 | FAM211A-AS1 | <1.00E-15 | 28.9 | down |
| ENST00000481456 | DDX39B | <1.00E-15 | 16.6 | down |
| ENST00000530564 | EIF4G2 | 1.65E-09 | 2.9 | down |
| ENST00000538654 | SNHG1 | <1.00E-15 | 20.7 | down |
| uc001yyb.4 | IPW | <1.00E-15 | 26.8 | down |
| ENST00000498999 | MTND4P12 | <1.00E-15 | 3.3 | down |
| ENST00000485706 | RPL7A | <1.00E-15 | 8.8 | down |
| ENST00000576468 | AJ003147.9 | 2.35E-08 | 2.2 | down |
| ENST00000473668 | RN7SL364P | 7.31E-11 | 3.5 | down |
| ENST00000513060 | GNB2L1 | <1.00E-15 | 21.1 | down |
| ENST00000481141 | RN7SL499P | <1.00E-15 | 6.2 | down |
| ENST00000513060 | GNB2L1 | <1.00E-15 | 21.1 | down |
| ENST00000497774 | FAM211A-AS1 | <1.00E-15 | 12.6 | down |
| ENST00000538654 | SNHG1 | <1.00E-15 | 21.6 | down |
| ENST00000579085 | EIF4A1 | 3.91E-09 | 5.9 | down |
| ENST00000607097 | MIR378D2 | 1.80E-07 | 3.9 | down |
| ENST00000576468 | AJ003147.9 | 2.35E-08 | 2.2 | down |
| ENST00000498589 | PPIL2 | 3.11E-15 | 3.3 | down |
| ENST00000513060 | GNB2L1 | <1.00E-15 | 21.1 | down |
| ENST00000496775 | NOP56 | <1.00E-15 | 39.9 | down |
| NR_001458 | MIR155HG | <1.00E-15 | 5.5 | down |
| ENST00000530585 | RPL27A | <1.00E-15 | 2.4 | down |
| uc001yxa.1 | SNURF | <1.00E-15 | 290.8 | down |
| ENST00000590268 | PPP1R12C | 3.41E-10 | 9.8 | down |
| NR_003604 | ZFAS1 | <1.00E-15 | 15.3 | down |
| ENST00000594728 | METTL12 | <1.00E-15 | 10.7 | down |
| ENST00000421068 | GAS5 | <1.00E-15 | 19.9 | down |
| ENST00000535568 | PGA5 | <1.00E-15 | 74.1 | down |
| ENST00000489408 | ZNF860 | <1.00E-15 | 7.9 | down |
| ENST00000517927 | MIR146A | <1.00E-15 | 5.6 | down |
| ENST00000514318 | GNB2L1 | <1.00E-15 | 23.4 | down |
| ENST00000421068 | GAS5 | <1.00E-15 | 19.9 | down |
| ENST00000536269 | PGA5 | <1.00E-15 | 38.1 | down |
| ENST00000579495 | RPL17 | <1.00E-15 | 13.2 | down |
| ENST00000566491 | RPL4 | <1.00E-15 | 36.7 | down |
| ENST00000477096 | ZRANB2 | <1.00E-15 | 3.5 | down |
| NR_038397 | DNM3OS | <1.00E-15 | 8.3 | down |
| ENST00000478473 | C9orf3 | <1.00E-15 | 2.6 | down |
| ENST00000563103 | CTD-2651B20.6 | 2.08E-06 | 10.1 | down |
| ENST00000607097 | MIR378D2 | 1.80E-07 | 3.9 | down |
| uc003nlp.1 | AK056211 | <1.00E-15 | 3.8 | down |
| ENST00000422293 | MTND2P9 | 9.47E-11 | 11.0 | down |
| ENST00000497899 | ENAH | 1.11E-08 | 6.5 | down |
| ENST00000595794 | C19orf48 | <1.00E-15 | 15.3 | down |
| ENST00000546204 | TCP1 | <1.00E-15 | 5.6 | down |
| ENST00000463926 | RN7SL43P | 1.04E-07 | 5.4 | down |
| ENST00000482732 | RPL10 | 4.96E-07 | 2.8 | down |
| ENST00000584901 | EIF4A1 | <1.00E-15 | 2.8 | down |
| ENST00000485101 | EIF4A2 | <1.00E-15 | 21.3 | down |
| ENST00000595794 | C19orf48 | <1.00E-15 | 15.3 | down |
| ENST00000600238 | RPL18A | <1.00E-15 | 6.7 | down |
| ENST00000513060 | GNB2L1 | <1.00E-15 | 21.1 | down |
| ENST00000498589 | PPIL2 | 3.11E-15 | 3.3 | down |
| ENST00000572453 | MIR497HG | <1.00E-15 | 4.4 | down |
| ENST00000529794 | TAF1D | <1.00E-15 | 4.2 | down |
| ENST00000431268 | GAS5 | <1.00E-15 | 14.5 | down |
| ENST00000485101 | EIF4A2 | <1.00E-15 | 21.3 | down |
| ENST00000467525 | FCGR2A | <1.00E-15 | 8.2 | down |
| ENST00000478473 | C9orf3 | <1.00E-15 | 2.6 | down |
| ENST00000520612 | MTND6P3 | <1.00E-15 | 6.0 | down |
| ENST00000521127 | SNHG6 | <1.00E-15 | 5.3 | down |
| ENST00000461448 | SNHG12 | <1.00E-15 | 2.1 | down |
| ENST00000470475 | RPS8 | <1.00E-15 | 9.7 | down |
| ENST00000578824 | ACADVL | <1.00E-15 | 9.1 | down |
| ENST00000595794 | C19orf48 | <1.00E-15 | 15.3 | down |
| ENST00000464971 | RN7SL308P | <1.00E-15 | 17.3 | down |
| ENST00000572453 | MIR497HG | <1.00E-15 | 4.4 | down |
| ENST00000538654 | SNHG1 | <1.00E-15 | 21.6 | down |
| ENST00000513060 | GNB2L1 | <1.00E-15 | 21.1 | down |
| ENST00000580921 | MED24 | <1.00E-15 | 47.0 | down |
| ENST00000579495 | RPL17 | <1.00E-15 | 13.2 | down |
| ENST00000565181 | RP11-1007O24.3 | 3.21E-09 | 20.9 | down |
| ENST00000485390 | RPS8 | <1.00E-15 | 36.3 | down |
| ENST00000579495 | RPL17 | <1.00E-15 | 13.2 | down |
| uc001yxa.1 | SNURF | <1.00E-15 | 290.8 | down |
| ENST00000511037 | MTND5P11 | 2.98E-12 | 2.8 | down |
| ENST00000462543 | RN7SL33P | 2.06E-06 | 9.4 | down |
| NR_027033 | MIRLET7BHG | <1.00E-15 | 8.3 | down |
| uc001yxn.4 | IPW | <1.00E-15 | 21.9 | down |
| NR_108100 | SPACA6P-AS | <1.00E-15 | 4.8 | down |
| ENST00000579085 | EIF4A1 | 3.91E-09 | 5.9 | down |
| ENST00000579085 | EIF4A1 | 3.91E-09 | 5.9 | down |
| ENST00000582718 | AC010761.8 | <1.00E-15 | 43.8 | down |
| ENST00000595794 | C19orf48 | <1.00E-15 | 15.3 | down |
| ENST00000579085 | EIF4A1 | 3.91E-09 | 5.9 | down |
| ENST00000538654 | SNHG1 | <1.00E-15 | 20.7 | down |
| ENST00000584901 | EIF4A1 | <1.00E-15 | 2.8 | down |
| ENST00000602652 | RP11-2B6.2 | <1.00E-15 | 9.5 | down |
| ENST00000474582 | RPS8 | <1.00E-15 | 12.5 | down |
| ENST00000421068 | GAS5 | <1.00E-15 | 7.8 | down |
| ENST00000565907 | RBMX | <1.00E-15 | 18.4 | down |
| ENST00000595794 | C19orf48 | <1.00E-15 | 15.3 | down |
| ENST00000576468 | AJ003147.9 | 2.35E-08 | 2.2 | down |
| ENST00000514318 | GNB2L1 | <1.00E-15 | 23.4 | down |
| ENST00000607097 | MIR378D2 | 1.80E-07 | 3.9 | down |
| ENST00000546204 | TCP1 | <1.00E-15 | 5.6 | down |
| ENST00000496775 | NOP56 | <1.00E-15 | 39.9 | down |
| ENST00000580921 | MED24 | <1.00E-15 | 47.0 | down |
| ENST00000485101 | EIF4A2 | <1.00E-15 | 21.3 | down |
| ENST00000482732 | RPL10 | 4.96E-07 | 2.8 | down |
| ENST00000602315 | MIR145 | <1.00E-15 | 3.6 | down |
| ENST00000421068 | GAS5 | <1.00E-15 | 19.9 | down |
| ENST00000578824 | ACADVL | <1.00E-15 | 9.1 | down |
| ENST00000467525 | FCGR2A | <1.00E-15 | 8.2 | down |
| NR_003672 | SNHG7 | 2.84E-04 | 2.3 | down |
| ENST00000576762 | AJ003147.8 | <1.00E-15 | 3.2 | down |
| ENST00000563749 | RPL13 | <1.00E-15 | 35.5 | down |
| ENST00000563749 | RPL13 | <1.00E-15 | 35.5 | down |
| ENST00000563749 | RPL13 | <1.00E-15 | 35.5 | down |
| ENST00000563749 | RPL13 | <1.00E-15 | 35.5 | down |
| ENST00000563749 | RPL13 | <1.00E-15 | 35.5 | down |
| ENST00000563749 | RPL13 | <1.00E-15 | 35.5 | down |
| ENST00000563749 | RPL13 | <1.00E-15 | 35.5 | down |
| ENST00000563749 | RPL13 | <1.00E-15 | 35.5 | down |
| Note: GC, gastric cancer; FC(abs), absolute fold change. | | | | |

| Table S2. The differentially expressed lncRNAs in GC | | | | | | | | | | |
| --- | --- | --- | --- | --- | --- | --- | --- | --- | --- | --- |
| Probe name | Gene symbol | GC-1 | CON-1 | GC-2 | CON-2 | GC-3 | CON-3 | *P* | FC(abs) | Regulation |
| OEV5_55319 | LINC00668 | 4.21 | 3.32 | 6.30 | 4.75 | 4.08 | 3.06 | 2.87E-02 | 2.2 | up |
| OEV5_44201 | CBFB | 3.41 | 2.27 | 4.48 | 2.86 | 4.11 | 2.78 | 1.01E-02 | 2.6 | up |
| OEV5_42905 | LOC101929475 | 4.32 | 3.48 | 4.05 | 2.50 | 3.65 | 2.69 | 3.62E-02 | 2.2 | up |
| A_33_P3418731 | ZNF542P | 2.81 | 1.98 | 2.88 | 1.98 | 3.72 | 2.17 | 4.17E-02 | 2.1 | up |
| OEV5_71319 | PXN-AS1 | 3.12 | 1.84 | 3.09 | 1.80 | 2.70 | 1.78 | 1.09E-02 | 2.2 | up |
| OEV5_77356 | LOC102723465 | 5.84 | 3.95 | 5.75 | 3.63 | 6.17 | 3.85 | 3.42E-03 | 4.3 | up |
| OEV5_48238 | LOC101929445 | 2.71 | 1.94 | 2.89 | 1.97 | 4.20 | 2.62 | 4.91E-02 | 2.1 | up |
| A_21_P0000348 | SNORA80A | 8.41 | 10.95 | 8.24 | 11.89 | 7.72 | 10.14 | 1.82E-02 | 7.3 | down |
| OEV5_38066 | LOC101928767 | 2.81 | 1.99 | 3.05 | 1.99 | 3.78 | 2.05 | 4.80E-02 | 2.3 | up |
| OEV5_39535 | RPS21 | 6.95 | 6.07 | 7.39 | 5.69 | 7.53 | 6.54 | 4.32E-02 | 2.3 | up |
| OEV5_44289 | CCDC101 | 5.51 | 3.62 | 3.59 | 2.72 | 4.01 | 2.36 | 4.12E-02 | 2.8 | up |
| OEV5_50914 | LINC01121 | 3.35 | 1.99 | 3.11 | 2.00 | 3.24 | 2.06 | 3.86E-03 | 2.3 | up |
| OEV5_36721 | TRAF2 | 4.20 | 1.98 | 3.54 | 2.01 | 3.39 | 2.07 | 2.45E-02 | 3.2 | up |
| OEV5_33753 | ABLIM3 | 2.79 | 2.00 | 3.69 | 1.97 | 3.80 | 2.24 | 4.15E-02 | 2.6 | up |
| OEV5_59943 | LOC101929633 | 6.62 | 8.10 | 6.31 | 7.75 | 5.96 | 6.60 | 4.90E-02 | 2.3 | down |
| A_21_P0012243 | CECR7 | 4.34 | 5.61 | 3.56 | 5.79 | 3.08 | 5.39 | 2.89E-02 | 3.8 | down |
| A_33_P3275973 | LOC100422737 | 2.75 | 3.93 | 2.30 | 4.30 | 2.84 | 4.38 | 2.21E-02 | 3.0 | down |
| OEV5_36604 | SUMF1 | 2.54 | 5.02 | 1.79 | 3.58 | 1.89 | 3.70 | 1.22E-02 | 4.1 | down |
| OEV5_36584 | TTLL7 | 2.59 | 4.35 | 2.74 | 4.49 | 3.31 | 5.20 | 6.42E-04 | 3.5 | down |
| A_33_P3322150 | LOC101927609 | 2.76 | 1.97 | 3.10 | 2.01 | 3.47 | 2.06 | 2.50E-02 | 2.1 | up |
| A_33_P3340613 | FAM95B1 | 4.87 | 5.84 | 2.82 | 4.25 | 4.70 | 5.40 | 3.95E-02 | 2.0 | down |
| OEV5_38200 | FANCA | 4.57 | 2.69 | 4.90 | 1.84 | 3.31 | 1.81 | 4.45E-02 | 4.4 | up |
| OEV5_63967 | EXOC3 | 4.91 | 7.32 | 3.61 | 5.97 | 4.58 | 5.71 | 4.25E-02 | 3.9 | down |
| OEV5_58491 | XXYLT1-AS2 | 2.74 | 4.37 | 2.67 | 4.28 | 4.01 | 5.00 | 2.14E-02 | 2.7 | down |
| OEV5_32733 | IL1RL1 | 2.72 | 4.57 | 2.79 | 3.95 | 3.02 | 4.20 | 2.48E-02 | 2.6 | down |
| OEV5_31657 | SFI1 | 8.44 | 7.12 | 7.68 | 6.80 | 8.24 | 7.31 | 1.85E-02 | 2.1 | up |
| OEV5_52401 | LINC01352 | 5.10 | 3.01 | 3.45 | 1.99 | 3.15 | 2.02 | 3.08E-02 | 2.9 | up |
| A_19_P00319929 | LINC01043 | 4.03 | 3.05 | 4.40 | 3.44 | 3.70 | 1.87 | 4.76E-02 | 2.4 | up |
| A_21_P0000437 | SNORD115-2 | 2.12 | 6.28 | 2.01 | 4.66 | 4.22 | 6.50 | 3.41E-02 | 8.2 | down |
| OEV5_32735 | TRPM7 | 3.94 | 3.05 | 4.26 | 3.04 | 3.59 | 2.53 | 8.08E-03 | 2.1 | up |
| OEV5_48793 | LIFR | 4.02 | 6.28 | 4.46 | 6.01 | 4.15 | 5.23 | 4.11E-02 | 3.1 | down |
| OEV5_46881 | ICAM2 | 7.91 | 13.10 | 8.03 | 14.08 | 9.98 | 12.61 | 4.59E-02 | 24.7 | down |
| OEV5_34772 | LINC00882 | 3.54 | 1.92 | 2.78 | 2.01 | 3.30 | 2.13 | 3.97E-02 | 2.3 | up |
| OEV5_72628 | ATOX1 | 2.69 | 1.96 | 3.52 | 1.97 | 3.57 | 2.31 | 3.92E-02 | 2.3 | up |
| OEV5_29450 | TUSC3 | 3.45 | 5.27 | 5.10 | 6.60 | 4.23 | 5.97 | 3.27E-03 | 3.2 | down |
| OEV5_54868 | CDKN2B-AS1 | 3.63 | 2.27 | 3.63 | 2.63 | 2.86 | 2.00 | 1.94E-02 | 2.1 | up |
| OEV5_73595 | TTLL7-IT1 | 2.05 | 4.45 | 1.89 | 3.11 | 1.79 | 4.42 | 4.16E-02 | 4.2 | down |
| OEV5_31245 | RELT | 5.00 | 2.72 | 4.53 | 3.46 | 4.71 | 2.32 | 4.55E-02 | 3.8 | up |
| OEV5_35276 | MYCBPAP | 2.72 | 1.74 | 3.04 | 1.79 | 3.73 | 2.78 | 8.14E-03 | 2.1 | up |
| OEV5_44832 | KIF18B | 6.11 | 4.59 | 4.95 | 3.98 | 5.79 | 4.22 | 1.94E-02 | 2.6 | up |
| OEV5_58148 | LOC257396 | 2.04 | 3.50 | 1.82 | 2.57 | 2.38 | 3.17 | 4.93E-02 | 2.0 | down |
| OEV5_50205 | LOC101929117 | 2.94 | 2.20 | 3.88 | 2.79 | 3.15 | 1.80 | 2.69E-02 | 2.1 | up |
| OEV5_65020 | LSM14B | 3.85 | 1.80 | 3.86 | 1.84 | 3.84 | 1.80 | 1.78E-05 | 4.1 | up |
| A_21_P0012096 | LOC101929378 | 2.46 | 1.85 | 5.69 | 4.24 | 3.36 | 2.12 | 4.92E-02 | 2.1 | up |
| A_21_P0000458 | SNORD115-28 | 2.86 | 5.22 | 1.82 | 4.47 | 2.72 | 4.43 | 1.52E-02 | 4.7 | down |
| A_24_P115529 | LOC51145 | 3.21 | 1.73 | 3.69 | 2.93 | 2.73 | 1.78 | 3.88E-02 | 2.1 | up |
| OEV5_42930 | LINC00472 | 2.35 | 4.87 | 3.02 | 4.77 | 1.82 | 3.90 | 1.10E-02 | 4.3 | down |
| A_21_P0009099 | LINC01373 | 2.12 | 3.62 | 1.76 | 3.34 | 1.78 | 2.88 | 1.11E-02 | 2.6 | down |
| A_21_P0006140 | LINC00950 | 2.14 | 3.82 | 1.77 | 2.96 | 1.78 | 2.66 | 3.27E-02 | 2.4 | down |
| OEV5_56439 | CALML3-AS1 | 7.55 | 5.61 | 6.91 | 5.08 | 6.57 | 5.45 | 2.39E-02 | 3.1 | up |
| A_19_P00315550 | LOC400043 | 9.87 | 11.31 | 9.90 | 11.05 | 9.87 | 10.70 | 2.35E-02 | 2.2 | down |
| OEV5_54962 | LOC100240735 | 5.17 | 6.80 | 5.25 | 6.37 | 5.24 | 6.23 | 2.33E-02 | 2.4 | down |
| OEV5_53666 | LINC01314 | 2.01 | 4.44 | 1.75 | 5.25 | 2.13 | 4.60 | 1.52E-02 | 7.0 | down |
| A_23_P170378 | PMCHL1 | 4.19 | 2.43 | 3.42 | 1.90 | 3.02 | 1.87 | 1.40E-02 | 2.8 | up |
| A_19_P00317815 | LOC101927934 | 4.33 | 2.65 | 5.71 | 2.50 | 5.04 | 1.79 | 3.42E-02 | 6.5 | up |
| OEV5_54254 | LOC339539 | 1.95 | 3.74 | 1.73 | 2.97 | 2.44 | 3.36 | 3.55E-02 | 2.5 | down |
| OEV5_31813 | ITSN1 | 4.58 | 3.00 | 3.35 | 2.13 | 3.78 | 2.22 | 6.30E-03 | 2.7 | up |
| A_32_P34826 | C21orf88 | 3.03 | 1.87 | 4.20 | 2.78 | 3.43 | 1.97 | 4.83E-03 | 2.5 | up |
| OEV5_78201 | UBE2R2-AS1 | 4.95 | 3.29 | 4.09 | 3.28 | 4.90 | 3.69 | 3.83E-02 | 2.3 | up |
| OEV5_50228 | PSG2 | 6.16 | 2.71 | 5.42 | 1.89 | 5.80 | 1.86 | 1.75E-03 | 12.5 | up |
| A_33_P3220872 | LOC1720 | 3.18 | 1.91 | 2.68 | 1.90 | 2.99 | 1.90 | 1.77E-02 | 2.1 | up |
| OEV5_52879 | TMEM75 | 3.56 | 2.31 | 3.74 | 2.68 | 3.68 | 2.53 | 2.34E-03 | 2.2 | up |
| OEV5_47719 | LOC101927787 | 3.63 | 1.78 | 3.06 | 1.83 | 2.67 | 1.78 | 4.17E-02 | 2.5 | up |
| OEV5_31916 | PRR5L | 4.90 | 3.97 | 5.54 | 3.95 | 5.08 | 4.22 | 4.09E-02 | 2.2 | up |
| OEV5_40040 | MIR7-3HG | 3.46 | 2.54 | 3.73 | 2.31 | 3.53 | 1.93 | 2.31E-02 | 2.5 | up |
| OEV5_57135 | SNHG17 | 8.86 | 12.89 | 9.29 | 14.09 | 8.96 | 12.29 | 1.09E-02 | 16.6 | down |
| OEV5_35060 | CCDC40 | 5.75 | 7.63 | 5.35 | 8.73 | 5.64 | 7.42 | 4.54E-02 | 5.1 | down |
| A_24_P6030 | SMIM11 | 2.43 | 5.73 | 1.77 | 5.20 | 1.87 | 3.66 | 3.27E-02 | 7.2 | down |
| OEV5_37806 | TSPAN2 | 4.14 | 3.08 | 3.84 | 1.78 | 3.89 | 2.26 | 3.21E-02 | 3.0 | up |
| OEV5_56824 | LAMTOR5-AS1 | 1.94 | 2.72 | 1.74 | 3.00 | 1.73 | 2.97 | 2.04E-02 | 2.1 | down |
| OEV5_46169 | C8orf46 | 1.93 | 5.10 | 1.74 | 4.69 | 2.79 | 4.68 | 2.14E-02 | 6.4 | down |
| A_21_P0014214 | CCAT1 | 5.12 | 3.89 | 6.16 | 3.83 | 7.09 | 4.98 | 3.04E-02 | 3.7 | up |
| OEV5_41521 | LYST | 9.14 | 8.12 | 9.10 | 7.89 | 9.57 | 8.62 | 5.40E-03 | 2.1 | up |
| OEV5_31312 | LOC101929771 | 4.79 | 3.20 | 4.40 | 3.29 | 3.60 | 2.52 | 1.69E-02 | 2.4 | up |
| A_21_P0014797 | LOC100507472 | 4.33 | 2.73 | 3.76 | 2.28 | 3.47 | 2.71 | 3.94E-02 | 2.4 | up |
| OEV5_27420 | HMG20B | 4.24 | 2.66 | 3.37 | 1.83 | 2.52 | 1.81 | 4.56E-02 | 2.4 | up |
| OEV5_53623 | LHFPL3-AS2 | 2.48 | 3.57 | 3.02 | 4.30 | 1.93 | 3.85 | 2.91E-02 | 2.7 | down |
| OEV5_46380 | TRIM24 | 3.62 | 4.89 | 3.50 | 5.55 | 3.90 | 4.86 | 4.73E-02 | 2.7 | down |
| OEV5_74568 | SNRPN | 4.77 | 7.85 | 4.25 | 6.32 | 5.31 | 7.76 | 1.36E-02 | 5.8 | down |
| OEV5_47267 | TBC1D9B | 4.10 | 3.43 | 3.04 | 1.79 | 3.20 | 1.96 | 3.11E-02 | 2.1 | up |
| OEV5_78012 | LOC101929416 | 5.07 | 6.23 | 4.02 | 5.61 | 4.98 | 5.74 | 3.95E-02 | 2.2 | down |
| OEV5_57392 | CSN1S2AP | 2.52 | 3.34 | 1.90 | 3.27 | 2.33 | 3.44 | 2.05E-02 | 2.1 | down |
| OEV5_55919 | LOC101929439 | 3.32 | 4.84 | 3.80 | 6.16 | 2.70 | 4.65 | 1.56E-02 | 3.8 | down |
| OEV5_36668 | TPD52L1 | 3.47 | 5.19 | 2.54 | 5.26 | 1.77 | 4.94 | 2.75E-02 | 5.8 | down |
| A_32_P169406 | LOC400043 | 5.45 | 9.58 | 6.21 | 9.60 | 6.99 | 8.74 | 4.85E-02 | 8.5 | down |
| OEV5_32447 | ATP10B | 4.62 | 2.31 | 6.40 | 2.80 | 5.71 | 3.59 | 2.86E-02 | 6.4 | up |
| OEV5_56267 | TRHDE-AS1 | 8.19 | 10.00 | 7.77 | 10.98 | 7.77 | 9.81 | 3.21E-02 | 5.1 | down |
| A_21_P0010543 | LOC101060442 | 4.65 | 3.56 | 3.81 | 2.21 | 4.10 | 1.75 | 4.52E-02 | 3.2 | up |
| OEV5_56704 | HMMR-AS1 | 6.42 | 5.14 | 5.72 | 4.78 | 6.51 | 5.51 | 9.47E-03 | 2.1 | up |
| OEV5_28738 | SPATA7 | 6.84 | 5.76 | 6.56 | 5.02 | 6.61 | 5.59 | 1.75E-02 | 2.3 | up |
| OEV5_35392 | APOL4 | 7.07 | 5.27 | 4.80 | 3.99 | 6.42 | 4.87 | 4.21E-02 | 2.6 | up |
| OEV5_58921 | HOXA-AS3 | 3.94 | 1.76 | 4.59 | 3.19 | 4.04 | 2.73 | 2.72E-02 | 3.1 | up |
| A_23_P70359 | AGPAT4-IT1 | 3.90 | 2.09 | 4.35 | 2.91 | 2.89 | 1.73 | 1.66E-02 | 2.8 | up |
| OEV5_58876 | WWTR1-AS1 | 1.83 | 3.29 | 2.66 | 3.71 | 1.69 | 3.09 | 9.59E-03 | 2.5 | down |
| A_33_P3361292 | LINC01105 | 1.89 | 5.06 | 1.70 | 4.98 | 1.71 | 4.20 | 6.87E-03 | 7.9 | down |
| OEV5_78442 | LOC100288152 | 4.21 | 5.74 | 3.42 | 5.50 | 4.27 | 5.79 | 1.15E-02 | 3.3 | down |
| OEV5_76929 | LOC102723716 | 1.90 | 3.45 | 2.18 | 2.85 | 1.72 | 2.97 | 4.62E-02 | 2.2 | down |
| OEV5_39558 | SMPD3 | 4.99 | 4.24 | 5.70 | 4.04 | 6.56 | 4.96 | 4.54E-02 | 2.5 | up |
| A_21_P0014221 | LOC100507464 | 7.95 | 12.90 | 7.93 | 13.90 | 7.98 | 12.29 | 8.98E-03 | 33.7 | down |
| OEV5_55827 | SNHG17 | 8.98 | 12.07 | 9.37 | 13.15 | 8.72 | 11.60 | 6.87E-03 | 9.5 | down |
| A_21_P0005851 | LOC102724687 | 2.62 | 5.83 | 1.68 | 5.03 | 2.09 | 3.93 | 2.83E-02 | 6.9 | down |
| A_33_P3576853 | NIFK-AS1 | 7.02 | 5.85 | 6.65 | 5.48 | 6.83 | 6.10 | 2.11E-02 | 2.0 | up |
| OEV5_59842 | XIST | 1.79 | 3.23 | 2.29 | 3.03 | 2.79 | 3.86 | 3.35E-02 | 2.1 | down |
| OEV5_32657 | LOC101927258 | 1.80 | 4.30 | 2.59 | 4.24 | 2.72 | 3.99 | 3.85E-02 | 3.5 | down |
| OEV5_27068 | ACADL | 1.73 | 3.72 | 1.66 | 2.67 | 1.64 | 4.02 | 4.73E-02 | 3.5 | down |
| OEV5_35307 | VAV2 | 3.77 | 2.96 | 3.32 | 2.16 | 3.70 | 2.36 | 1.95E-02 | 2.1 | up |
| OEV5_53238 | LOC102724301 | 4.13 | 2.59 | 4.07 | 3.09 | 3.32 | 1.69 | 2.06E-02 | 2.6 | up |
| OEV5_59246 | LINC01207 | 7.19 | 3.22 | 7.08 | 2.85 | 6.35 | 3.00 | 4.57E-03 | 14.4 | up |
| OEV5_52169 | LINC00910 | 3.59 | 2.28 | 4.01 | 3.34 | 3.99 | 2.57 | 4.03E-02 | 2.2 | up |
| A_33_P3287862 | LOC101060524 | 6.45 | 10.09 | 5.44 | 9.02 | 5.74 | 7.63 | 3.40E-02 | 8.2 | down |
| OEV5_78441 | LOC100288152 | 3.73 | 5.71 | 3.75 | 5.71 | 4.12 | 5.60 | 7.91E-03 | 3.5 | down |
| OEV5_40632 | ERC2 | 1.77 | 3.05 | 3.03 | 3.84 | 2.01 | 3.63 | 3.45E-02 | 2.4 | down |
| OEV5_56835 | LOC101927746 | 5.38 | 3.29 | 3.92 | 2.60 | 4.44 | 3.31 | 3.63E-02 | 2.9 | up |
| OEV5_76987 | LOC100288152 | 3.66 | 5.25 | 3.58 | 4.57 | 3.68 | 4.92 | 1.82E-02 | 2.4 | down |
| A_21_P0002013 | LOC100996549 | 1.79 | 2.75 | 2.46 | 4.52 | 1.81 | 3.90 | 4.45E-02 | 3.3 | down |
| OEV5_56876 | KLF3-AS1 | 1.70 | 4.44 | 4.06 | 5.85 | 2.44 | 4.21 | 2.23E-02 | 4.3 | down |
| OEV5_43892 | APOL2 | 8.12 | 6.20 | 6.23 | 4.78 | 7.54 | 5.71 | 6.78E-03 | 3.3 | up |
| OEV5_40294 | RIMKLB | 1.69 | 2.93 | 1.86 | 2.98 | 2.26 | 3.46 | 9.25E-04 | 2.3 | down |
| OEV5_30885 | C8orf46 | 1.76 | 4.76 | 1.64 | 4.12 | 2.04 | 4.14 | 1.02E-02 | 5.8 | down |
| A_33_P3286492 | HNF4G | 9.05 | 7.74 | 9.51 | 7.77 | 8.46 | 7.54 | 3.08E-02 | 2.5 | up |
| A_21_P0002325 | LOC101927156 | 3.20 | 4.89 | 3.41 | 4.44 | 4.74 | 5.57 | 4.46E-02 | 2.3 | down |
| OEV5_39041 | LOC81691 | 7.49 | 10.08 | 6.77 | 11.13 | 6.68 | 9.32 | 3.15E-02 | 9.2 | down |
| OEV5_76935 | LOC102724092 | 1.68 | 2.89 | 2.32 | 3.40 | 1.59 | 2.55 | 4.63E-03 | 2.1 | down |
| OEV5_49208 | CNIH3 | 3.85 | 6.39 | 4.65 | 8.02 | 3.82 | 5.49 | 3.55E-02 | 5.8 | down |
| A_21_P0003227 | LOC100506319 | 1.68 | 2.66 | 1.61 | 2.29 | 1.62 | 3.00 | 3.77E-02 | 2.0 | down |
| OEV5_77128 | LOC101928529 | 1.67 | 2.83 | 1.61 | 3.15 | 1.61 | 2.47 | 2.65E-02 | 2.3 | down |
| OEV5_77388 | LOC101927063 | 5.60 | 8.05 | 5.80 | 9.65 | 5.68 | 8.23 | 2.27E-02 | 7.7 | down |
| OEV5_40625 | PRC1 | 3.91 | 1.67 | 2.82 | 1.67 | 3.08 | 1.64 | 3.87E-02 | 3.0 | up |
| OEV5_38810 | IGFBP3 | 5.66 | 3.99 | 5.77 | 4.67 | 5.74 | 4.47 | 1.60E-02 | 2.5 | up |
| A_33_P3405606 | CASC8 | 1.69 | 3.60 | 2.24 | 3.26 | 1.60 | 2.84 | 3.48E-02 | 2.6 | down |
| OEV5_52819 | LINC01105 | 2.98 | 4.28 | 1.60 | 3.29 | 2.45 | 3.91 | 5.52E-03 | 2.8 | down |
| OEV5_53003 | LINC01105 | 1.65 | 4.46 | 1.60 | 5.44 | 1.61 | 4.05 | 1.88E-02 | 8.2 | down |
| A_21_P0002254 | LOC101928113 | 1.63 | 3.04 | 1.60 | 3.01 | 2.49 | 3.12 | 4.64E-02 | 2.2 | down |
| A_33_P3329128 | LOC729930 | 1.69 | 3.05 | 1.86 | 2.97 | 1.61 | 2.81 | 3.59E-03 | 2.3 | down |
| OEV5_43900 | CECR5 | 5.80 | 4.50 | 4.97 | 3.76 | 3.88 | 3.13 | 2.37E-02 | 2.1 | up |
| OEV5_41934 | LOC101928858 | 1.68 | 3.71 | 2.52 | 5.26 | 1.60 | 3.97 | 7.44E-03 | 5.2 | down |
| OEV5_55123 | LOC101929655 | 1.63 | 5.89 | 2.84 | 7.68 | 2.70 | 5.48 | 2.31E-02 | 15.6 | down |
| A_33_P3262625 | CECR7 | 4.24 | 5.75 | 2.69 | 5.21 | 3.14 | 5.11 | 2.05E-02 | 4.0 | down |
| OEV5_34353 | HSP90AA1 | 7.51 | 3.51 | 5.26 | 2.45 | 4.80 | 2.56 | 2.82E-02 | 8.1 | up |
| OEV5_57247 | LOC101927391 | 1.65 | 4.50 | 1.56 | 4.08 | 1.77 | 3.77 | 9.79E-03 | 5.5 | down |
| OEV5_55795 | LINC01082 | 1.66 | 2.97 | 2.00 | 3.15 | 2.90 | 4.36 | 4.65E-03 | 2.5 | down |
| OEV5_49761 | GSTM5 | 4.93 | 7.32 | 4.28 | 6.78 | 3.23 | 4.51 | 3.38E-02 | 4.2 | down |
| OEV5_43523 | CASP1 | 4.73 | 3.51 | 2.95 | 1.63 | 3.76 | 3.20 | 4.92E-02 | 2.1 | up |
| OEV5_58660 | NTM-IT | 4.09 | 2.55 | 3.49 | 2.79 | 3.58 | 2.55 | 4.60E-02 | 2.1 | up |
| A_33_P3335535 | LOC100128682 | 1.64 | 3.28 | 2.79 | 4.01 | 2.43 | 3.15 | 4.68E-02 | 2.3 | down |
| OEV5_39516 | MACC1 | 2.81 | 2.00 | 5.25 | 3.78 | 4.06 | 2.86 | 2.53E-02 | 2.2 | up |
| OEV5_38916 | EPB41L4A | 1.66 | 4.66 | 1.88 | 6.16 | 2.01 | 4.22 | 3.45E-02 | 9.0 | down |
| A_21_P0005628 | CCAT1 | 6.15 | 2.84 | 7.05 | 4.29 | 8.22 | 5.50 | 4.22E-03 | 7.6 | up |
| A_19_P00809335 | C8orf46 | 1.59 | 4.85 | 1.83 | 4.92 | 3.10 | 4.77 | 3.35E-02 | 6.4 | down |
| OEV5_65810 | COTL1 | 7.99 | 6.63 | 6.50 | 5.70 | 7.73 | 6.53 | 2.14E-02 | 2.2 | up |
| OEV5_56726 | LINC00939 | 2.92 | 1.69 | 4.81 | 2.27 | 4.06 | 1.99 | 3.65E-02 | 3.9 | up |
| OEV5_28243 | DKK1 | 1.60 | 2.91 | 1.57 | 2.84 | 2.74 | 3.66 | 1.11E-02 | 2.2 | down |
| OEV5_27919 | UROS | 5.46 | 4.60 | 5.26 | 3.65 | 5.74 | 4.89 | 4.79E-02 | 2.2 | up |
| OEV5_45982 | LPHN3 | 1.60 | 2.89 | 1.56 | 2.95 | 3.37 | 4.37 | 8.93E-03 | 2.3 | down |
| OEV5_53205 | CASC11 | 3.01 | 1.77 | 3.42 | 1.60 | 2.72 | 1.72 | 3.15E-02 | 2.6 | up |
| A_33_P3406796 | C5orf66-AS1 | 3.91 | 5.23 | 3.72 | 5.78 | 4.96 | 6.45 | 1.84E-02 | 3.1 | down |
| A_33_P3312534 | TRAPPC10 | 3.38 | 2.57 | 3.74 | 2.10 | 4.42 | 3.49 | 4.85E-02 | 2.2 | up |
| OEV5_48544 | MT1X | 4.90 | 8.00 | 5.11 | 9.34 | 4.64 | 7.05 | 2.54E-02 | 9.5 | down |
| OEV5_46939 | MKLN1 | 3.37 | 4.54 | 3.45 | 4.24 | 3.12 | 4.32 | 1.49E-02 | 2.1 | down |
| OEV5_45119 | KCTD1 | 1.57 | 3.94 | 1.56 | 3.76 | 2.53 | 3.54 | 4.91E-02 | 3.6 | down |
| OEV5_56274 | LINC00710 | 5.07 | 7.45 | 4.23 | 6.41 | 5.58 | 7.09 | 1.64E-02 | 4.1 | down |
| OEV5_55702 | LOC100506834 | 1.59 | 3.50 | 3.44 | 4.39 | 1.57 | 3.74 | 4.55E-02 | 3.2 | down |
| OEV5_55725 | LINC00486 | 2.94 | 1.57 | 2.83 | 1.58 | 3.26 | 1.89 | 9.11E-04 | 2.5 | up |
| A_33_P3264200 | EGFEM1P | 1.58 | 2.80 | 2.10 | 3.40 | 3.56 | 4.63 | 3.21E-03 | 2.3 | down |
| OEV5_45572 | LRRTM3 | 4.97 | 2.49 | 2.92 | 1.58 | 3.63 | 2.30 | 4.56E-02 | 3.3 | up |
| OEV5_28245 | EIF3C | 6.62 | 8.86 | 6.27 | 9.94 | 6.01 | 8.04 | 3.57E-02 | 6.3 | down |
| OEV5_42101 | LMBR1 | 1.59 | 3.55 | 2.80 | 3.71 | 1.57 | 2.91 | 4.41E-02 | 2.6 | down |
| OEV5_42628 | LIMK2 | 6.97 | 5.80 | 6.08 | 4.87 | 6.58 | 5.89 | 2.58E-02 | 2.0 | up |
| OEV5_44331 | IGHMBP2 | 3.58 | 2.58 | 3.63 | 2.28 | 3.40 | 2.67 | 2.94E-02 | 2.0 | up |
| OEV5_58146 | CCAT1 | 5.91 | 3.62 | 6.88 | 3.99 | 8.11 | 5.81 | 6.39E-03 | 5.6 | up |
| OEV5_59134 | RAET1E-AS1 | 2.96 | 4.48 | 3.92 | 4.65 | 1.57 | 2.89 | 3.76E-02 | 2.3 | down |
| OEV5_44720 | CEP85 | 3.04 | 1.58 | 2.88 | 1.57 | 3.35 | 1.56 | 8.30E-03 | 2.9 | up |
| OEV5_46206 | PTPRZ1 | 1.61 | 4.49 | 1.57 | 3.69 | 1.59 | 3.24 | 2.49E-02 | 4.6 | down |
| A_21_P0011733 | LOC100505851 | 1.68 | 2.62 | 2.03 | 3.67 | 1.87 | 3.35 | 2.35E-02 | 2.6 | down |
| OEV5_57924 | LINC01144 | 7.34 | 5.54 | 6.90 | 5.83 | 6.90 | 5.89 | 3.65E-02 | 2.5 | up |
| OEV5_45290 | ZNF91 | 2.70 | 4.00 | 2.84 | 4.40 | 2.72 | 3.71 | 1.60E-02 | 2.4 | down |
| OEV5_46887 | C16orf97 | 3.14 | 1.57 | 2.89 | 1.58 | 3.86 | 1.97 | 1.09E-02 | 3.0 | up |
| OEV5_38922 | PRPF40B | 4.08 | 3.15 | 4.05 | 3.07 | 2.68 | 1.56 | 3.35E-03 | 2.0 | up |
| OEV5_44676 | PAN2 | 1.58 | 3.05 | 2.05 | 3.61 | 2.05 | 2.71 | 4.96E-02 | 2.3 | down |
| OEV5_37926 | CLIC5 | 2.88 | 1.79 | 3.50 | 2.30 | 3.16 | 1.88 | 1.99E-03 | 2.3 | up |
| A_19_P00807328 | LOC102724209 | 2.84 | 1.58 | 3.17 | 1.78 | 3.43 | 1.59 | 1.37E-02 | 2.8 | up |
| OEV5_29147 | LONRF1 | 6.14 | 7.89 | 6.01 | 9.10 | 5.97 | 7.53 | 4.70E-02 | 4.4 | down |
| OEV5_46258 | CASP1 | 4.66 | 3.75 | 3.29 | 1.61 | 4.56 | 3.14 | 2.71E-02 | 2.5 | up |
| OEV5_54459 | LOC100631378 | 2.89 | 1.53 | 3.18 | 1.56 | 2.73 | 1.58 | 9.53E-03 | 2.6 | up |
| A_21_P0012518 | LINC01206 | 1.55 | 2.76 | 1.60 | 2.39 | 1.83 | 2.95 | 1.50E-02 | 2.1 | down |
| OEV5_31565 | FARP1 | 5.34 | 7.24 | 6.26 | 7.50 | 5.74 | 6.71 | 3.86E-02 | 2.6 | down |
| OEV5_54382 | LOC100287098 | 4.06 | 1.53 | 3.21 | 1.56 | 4.04 | 2.66 | 3.36E-02 | 3.6 | up |
| OEV5_44673 | SP3 | 4.89 | 2.26 | 2.86 | 1.58 | 4.04 | 1.59 | 3.77E-02 | 4.3 | up |
| OEV5_64990 | AXDND1 | 1.70 | 3.38 | 1.60 | 3.17 | 2.41 | 3.36 | 2.49E-02 | 2.6 | down |
| OEV5_42882 | FARP1 | 3.88 | 4.83 | 4.27 | 5.53 | 3.74 | 4.90 | 6.32E-03 | 2.2 | down |
| OEV5_57775 | SNHG17 | 7.70 | 12.29 | 8.13 | 13.13 | 7.76 | 11.48 | 7.25E-03 | 21.6 | down |
| OEV5_59259 | FAR2P2 | 4.09 | 2.76 | 4.79 | 3.35 | 4.63 | 3.94 | 3.91E-02 | 2.2 | up |
| OEV5_58094 | DIRC3 | 1.71 | 3.69 | 2.57 | 3.78 | 1.65 | 3.06 | 2.15E-02 | 2.9 | down |
| OEV5_39762 | S100A10 | 11.28 | 10.08 | 11.63 | 9.55 | 11.98 | 10.72 | 3.35E-02 | 2.9 | up |
| OEV5_51975 | ATXN8OS | 8.28 | 6.43 | 7.80 | 5.94 | 7.57 | 6.62 | 3.52E-02 | 2.9 | up |
| OEV5_33944 | LOC401410 | 3.34 | 6.09 | 2.75 | 4.46 | 1.57 | 3.85 | 1.75E-02 | 4.7 | down |
| OEV5_30315 | VPS72 | 5.69 | 4.53 | 5.10 | 4.28 | 5.61 | 4.25 | 1.96E-02 | 2.2 | up |
| OEV5_55578 | CTSLP2 | 1.67 | 3.63 | 3.05 | 3.90 | 1.79 | 3.17 | 4.81E-02 | 2.6 | down |
| OEV5_52062 | WDFY3-AS2 | 1.59 | 4.09 | 2.61 | 4.10 | 2.51 | 4.37 | 2.21E-02 | 3.9 | down |
| OEV5_46480 | CECR7 | 3.33 | 5.85 | 2.58 | 5.17 | 2.38 | 5.31 | 2.19E-03 | 6.4 | down |
| OEV5_44801 | COL7A1 | 5.38 | 1.54 | 5.44 | 2.16 | 3.99 | 1.58 | 1.67E-02 | 9.0 | up |
| OEV5_41661 | LOC101927946 | 1.56 | 2.90 | 1.83 | 3.34 | 1.56 | 2.22 | 4.60E-02 | 2.3 | down |
| A_21_P0000850 | FAM212B-AS1 | 1.83 | 3.06 | 1.83 | 2.67 | 1.67 | 3.40 | 3.88E-02 | 2.4 | down |
| OEV5_43873 | FLJ43585 | 3.01 | 3.83 | 2.95 | 4.19 | 3.06 | 4.30 | 1.62E-02 | 2.1 | down |
| OEV5_33854 | LOC155060 | 3.46 | 5.19 | 4.44 | 6.07 | 3.11 | 5.04 | 2.42E-03 | 3.4 | down |
| OEV5_43896 | ABCA8 | 3.55 | 5.00 | 3.75 | 4.68 | 4.10 | 5.07 | 2.17E-02 | 2.2 | down |
| A_23_P63736 | LINC00839 | 4.42 | 3.56 | 4.43 | 3.31 | 4.46 | 3.34 | 6.87E-03 | 2.0 | up |
| OEV5_50022 | MST1 | 4.78 | 6.88 | 4.99 | 6.11 | 5.28 | 6.79 | 3.13E-02 | 3.0 | down |
| A_21_P0006456 | XIST | 3.41 | 4.33 | 1.77 | 3.40 | 2.40 | 4.04 | 2.80E-02 | 2.6 | down |
| A_21_P0007509 | THRIL | 6.27 | 5.31 | 5.18 | 4.46 | 6.27 | 4.84 | 3.78E-02 | 2.0 | up |
| OEV5_54160 | CASC9 | 3.94 | 2.90 | 4.69 | 3.29 | 3.10 | 1.70 | 8.81E-03 | 2.4 | up |
| OEV5_54233 | LOC102724484 | 4.13 | 3.14 | 3.95 | 2.78 | 3.66 | 2.44 | 3.73E-03 | 2.2 | up |
| OEV5_57321 | CHL1-AS1 | 1.77 | 3.19 | 1.65 | 2.90 | 1.70 | 3.36 | 7.00E-03 | 2.7 | down |
| A_24_P352116 | SNHG7 | 4.19 | 3.62 | 5.03 | 3.83 | 4.49 | 3.23 | 4.53E-02 | 2.0 | up |
| OEV5_55704 | LOC101927450 | 6.97 | 5.68 | 4.20 | 3.26 | 5.78 | 4.54 | 9.37E-03 | 2.2 | up |
| OEV5_44567 | PTPRK | 7.87 | 10.68 | 8.61 | 11.72 | 8.32 | 10.26 | 1.72E-02 | 6.2 | down |
| A_21_P0001816 | LOC400940 | 1.64 | 3.85 | 1.62 | 3.57 | 1.63 | 2.80 | 2.96E-02 | 3.4 | down |
| OEV5_51148 | FLJ20444 | 2.57 | 4.66 | 2.83 | 6.02 | 3.35 | 4.94 | 3.97E-02 | 4.9 | down |
| A_33_P3225630 | IGFL4 | 3.03 | 1.72 | 2.64 | 1.71 | 2.95 | 1.74 | 9.37E-03 | 2.2 | up |
| OEV5_37689 | KATNBL1 | 5.80 | 4.80 | 4.65 | 2.67 | 5.31 | 3.77 | 3.37E-02 | 2.8 | up |
| A_21_P0005830 | LOC100507464 | 8.40 | 12.82 | 8.04 | 13.80 | 7.97 | 12.19 | 9.96E-03 | 27.8 | down |
| A_19_P00805218 | LINC01197 | 4.47 | 3.34 | 4.65 | 3.33 | 2.94 | 2.33 | 4.03E-02 | 2.0 | up |
| OEV5_49646 | LOC101929408 | 4.16 | 5.03 | 3.49 | 4.49 | 3.34 | 4.55 | 9.52E-03 | 2.0 | down |
| OEV5_54406 | LINC00226 | 1.90 | 2.71 | 1.71 | 3.14 | 1.76 | 2.88 | 2.52E-02 | 2.2 | down |
| A_33_P3632937 | LOC100131262 | 5.29 | 4.47 | 5.42 | 4.09 | 4.70 | 3.79 | 2.31E-02 | 2.0 | up |
| OEV5_27333 | MT1X | 3.10 | 6.71 | 3.89 | 8.29 | 3.34 | 5.96 | 2.05E-02 | 11.6 | down |
| OEV5_53492 | LOC100132215 | 3.11 | 1.71 | 2.92 | 1.68 | 3.43 | 1.84 | 4.86E-03 | 2.7 | up |
| A_21_P0001189 | LOC100505918 | 1.70 | 2.62 | 2.26 | 3.51 | 1.67 | 2.80 | 7.45E-03 | 2.1 | down |
| OEV5_44067 | SOX6 | 3.80 | 5.26 | 4.03 | 5.00 | 4.37 | 5.88 | 1.66E-02 | 2.5 | down |
| OEV5_40565 | LINC00923 | 6.59 | 10.98 | 5.60 | 12.18 | 5.95 | 10.39 | 1.92E-02 | 35.2 | down |
| OEV5_58971 | LINC01197 | 1.73 | 3.60 | 3.80 | 5.99 | 1.90 | 4.54 | 9.73E-03 | 4.7 | down |
| OEV5_57643 | LINC00640 | 3.54 | 2.13 | 2.42 | 1.71 | 2.80 | 1.74 | 3.41E-02 | 2.1 | up |
| A_21_P0002212 | LOC100506235 | 6.50 | 7.95 | 6.23 | 9.24 | 5.69 | 7.69 | 4.25E-02 | 4.4 | down |
| OEV5_57896 | LOC101928167 | 1.97 | 3.05 | 1.73 | 3.19 | 2.38 | 3.35 | 1.63E-02 | 2.2 | down |
| A_21_P0000438 | SNORD115-2 | 3.64 | 6.94 | 3.60 | 5.46 | 4.43 | 6.65 | 2.99E-02 | 5.5 | down |
| OEV5_59571 | PP7080 | 9.75 | 11.31 | 9.39 | 10.66 | 9.66 | 10.33 | 4.69E-02 | 2.2 | down |
| OEV5_57218 | LINC00877 | 1.77 | 3.24 | 1.85 | 2.99 | 2.66 | 3.43 | 3.07E-02 | 2.2 | down |
| A_21_P0000681 | LIMS3-LOC440895 | 3.61 | 2.04 | 3.68 | 2.83 | 3.21 | 2.02 | 2.92E-02 | 2.3 | up |
| OEV5_77628 | LOC643327 | 1.79 | 3.27 | 2.91 | 4.32 | 2.30 | 2.94 | 4.90E-02 | 2.3 | down |
| OEV5_61319 | BHLHE40-AS1 | 4.49 | 2.92 | 4.59 | 3.54 | 4.23 | 3.19 | 1.99E-02 | 2.3 | up |
| OEV5_53647 | RBFADN | 3.51 | 2.33 | 4.46 | 3.50 | 3.20 | 2.16 | 3.68E-03 | 2.1 | up |
| OEV5_28377 | SLC37A3 | 1.78 | 3.28 | 2.07 | 4.76 | 1.72 | 3.38 | 3.43E-02 | 3.9 | down |
| A_21_P0014098 | IDH1-AS1 | 4.87 | 3.88 | 4.31 | 3.37 | 4.43 | 3.29 | 3.30E-03 | 2.0 | up |
| A_33_P3282359 | ANKRD20A9P | 2.10 | 3.79 | 1.90 | 3.93 | 2.99 | 3.89 | 4.49E-02 | 2.9 | down |
| OEV5_58937 | ZNF674-AS1 | 3.66 | 2.16 | 5.07 | 3.78 | 4.07 | 3.33 | 3.49E-02 | 2.3 | up |
| OEV5_48672 | NCALD | 3.73 | 5.27 | 4.49 | 5.90 | 3.44 | 5.08 | 1.79E-03 | 2.9 | down |
| A_21_P0012308 | CECR7 | 1.81 | 5.81 | 2.89 | 5.12 | 2.66 | 5.11 | 3.54E-02 | 7.4 | down |
| OEV5_34519 | GLCCI1 | 4.16 | 2.69 | 3.21 | 1.72 | 2.82 | 2.16 | 4.83E-02 | 2.3 | up |
| OEV5_40032 | TRMU | 5.92 | 3.67 | 5.10 | 2.89 | 5.72 | 4.01 | 7.21E-03 | 4.2 | up |
| OEV5_54840 | PP7080 | 9.80 | 11.06 | 9.38 | 10.54 | 9.66 | 10.35 | 2.72E-02 | 2.1 | down |
| OEV5_53612 | DLEU1 | 5.23 | 4.19 | 5.38 | 4.28 | 5.40 | 4.34 | 3.49E-04 | 2.1 | up |
| OEV5_54775 | RAPGEF4-AS1 | 1.86 | 3.00 | 1.97 | 3.32 | 1.75 | 2.93 | 2.70E-03 | 2.3 | down |
| OEV5_55117 | LOC101927637 | 2.04 | 6.12 | 1.73 | 4.30 | 1.84 | 4.34 | 2.74E-02 | 8.3 | down |
| OEV5_34260 | RAB21 | 3.02 | 1.76 | 2.87 | 1.72 | 3.86 | 3.20 | 3.02E-02 | 2.0 | up |
| A_33_P3356361 | SRP14-AS1 | 2.41 | 4.73 | 3.85 | 5.03 | 1.94 | 3.22 | 4.89E-02 | 3.0 | down |
| OEV5_52985 | RASAL2-AS1 | 4.33 | 2.63 | 3.69 | 2.48 | 3.42 | 2.37 | 2.18E-02 | 2.5 | up |
| OEV5_36399 | FOXRED1 | 3.08 | 4.01 | 2.98 | 4.29 | 2.36 | 3.44 | 9.94E-03 | 2.1 | down |
| A_21_P0012412 | EGFEM1P | 1.83 | 4.27 | 1.70 | 3.52 | 3.41 | 5.50 | 7.21E-03 | 4.3 | down |
| OEV5_33926 | SMIM11 | 4.93 | 7.31 | 4.85 | 7.01 | 4.99 | 6.13 | 3.78E-02 | 3.7 | down |
| OEV5_53276 | LINC01105 | 5.64 | 6.70 | 4.02 | 6.42 | 3.25 | 5.70 | 4.94E-02 | 3.9 | down |
| OEV5_56774 | LOC102467213 | 4.83 | 6.21 | 4.06 | 5.32 | 3.94 | 5.41 | 2.09E-03 | 2.6 | down |
| OEV5_69467 | LOC101929753 | 4.42 | 2.80 | 2.54 | 1.74 | 4.05 | 2.83 | 3.62E-02 | 2.3 | up |
| A_19_P00316897 | CTD-3080P12.3 | 4.58 | 3.16 | 3.42 | 1.75 | 2.96 | 2.08 | 2.94E-02 | 2.5 | up |
| OEV5_57747 | LOC286189 | 2.41 | 3.68 | 3.05 | 4.32 | 3.43 | 4.19 | 2.30E-02 | 2.1 | down |
| OEV5_50373 | KATNBL1 | 5.85 | 4.89 | 4.90 | 3.66 | 5.65 | 4.79 | 1.20E-02 | 2.0 | up |
| Note: GC, gastric cancer; FC(abs), absolute fold change. | | | | | | | | | | |

| Table S3. The differentially expressed mRNAs in GC | | | | | | | | | | |
| --- | --- | --- | --- | --- | --- | --- | --- | --- | --- | --- |
| Probe name | Gene symbol | GC-1 | CON-1 | GC-2 | CON-2 | GC-3 | CON-3 | *P* | FC(abs) | Regulation |
| A_23_P15174 | MT1F | 9.19 | 11.14 | 8.09 | 11.81 | 8.57 | 11.21 | 3.28E-02 | 6.8 | down |
| A_23_P60079 | ANGPT2 | 5.69 | 3.78 | 6.10 | 2.24 | 7.20 | 4.88 | 4.52E-02 | 6.5 | up |
| A_23_P89799 | ACAA2 | 8.11 | 7.18 | 8.12 | 6.44 | 8.02 | 7.01 | 3.68E-02 | 2.3 | up |
| A_23_P137391 | ENO1 | 11.59 | 10.26 | 10.99 | 9.89 | 11.41 | 10.72 | 3.07E-02 | 2.1 | up |
| A_33_P3271241 | PPA1 | 10.90 | 9.80 | 10.74 | 9.40 | 11.06 | 10.03 | 6.38E-03 | 2.2 | up |
| A_23_P80902 | KIF15 | 5.59 | 3.47 | 4.55 | 3.33 | 5.32 | 4.02 | 3.24E-02 | 2.9 | up |
| A_23_P141092 | TFAP4 | 5.88 | 4.76 | 5.75 | 4.20 | 4.94 | 4.20 | 4.00E-02 | 2.2 | up |
| A_23_P161152 | PDSS1 | 7.24 | 6.25 | 7.87 | 5.97 | 7.19 | 6.16 | 4.80E-02 | 2.5 | up |
| A_23_P253752 | MTFR2 | 5.33 | 2.60 | 5.55 | 1.82 | 5.23 | 3.64 | 4.88E-02 | 6.4 | up |
| A_32_P186474 | RACGAP1 | 3.89 | 1.95 | 3.26 | 1.98 | 3.64 | 2.00 | 1.37E-02 | 3.1 | up |
| A_24_P260639 | HIST1H1D | 9.81 | 8.39 | 9.26 | 7.30 | 9.61 | 8.42 | 2.17E-02 | 2.9 | up |
| A_33_P3222228 | FUT6 | 7.20 | 4.97 | 7.77 | 5.32 | 7.75 | 5.06 | 2.99E-03 | 5.5 | up |
| A_23_P3681 | NETO2 | 6.81 | 5.44 | 6.36 | 5.13 | 5.62 | 4.81 | 2.13E-02 | 2.2 | up |
| A_23_P130811 | ZNF536 | 2.30 | 4.32 | 1.75 | 3.60 | 5.20 | 6.48 | 1.71E-02 | 3.3 | down |
| A_23_P421379 | IGF2 | 6.85 | 5.31 | 7.52 | 5.91 | 7.71 | 6.28 | 1.20E-03 | 2.9 | up |
| A_32_P213831 | STRIP2 | 5.94 | 3.81 | 3.97 | 2.66 | 4.78 | 3.07 | 1.85E-02 | 3.3 | up |
| A_23_P7402 | PDZD2 | 4.06 | 5.24 | 5.21 | 6.75 | 5.46 | 6.12 | 4.73E-02 | 2.2 | down |
| A_23_P69537 | NMU | 4.30 | 2.62 | 3.45 | 2.27 | 4.44 | 2.97 | 9.83E-03 | 2.7 | up |
| A_24_P282251 | FGA | 3.20 | 8.43 | 1.81 | 7.68 | 2.63 | 6.22 | 1.86E-02 | 29.7 | down |
| A_23_P157926 | LINGO2 | 2.00 | 4.44 | 2.64 | 4.92 | 2.56 | 4.11 | 1.69E-02 | 4.3 | down |
| A_33_P3238969 | ONECUT3 | 8.72 | 3.59 | 6.05 | 3.52 | 7.45 | 4.46 | 4.74E-02 | 11.7 | up |
| A_23_P323924 | ZFYVE27 | 6.71 | 12.03 | 6.38 | 13.10 | 6.67 | 11.27 | 1.24E-02 | 46.8 | down |
| A_24_P331704 | KRT80 | 4.05 | 2.28 | 4.50 | 3.19 | 5.34 | 2.72 | 3.86E-02 | 3.7 | up |
| A_33_P3314341 | KIAA1958 | 5.11 | 6.91 | 5.36 | 7.38 | 5.12 | 6.35 | 1.85E-02 | 3.2 | down |
| A_23_P74349 | NUF2 | 7.22 | 5.00 | 6.66 | 4.84 | 6.45 | 5.37 | 3.66E-02 | 3.3 | up |
| A_33_P3239228 | MUC3A | 8.67 | 7.23 | 9.20 | 7.15 | 8.30 | 7.41 | 4.92E-02 | 2.8 | up |
| A_23_P430728 | ATP4A | 2.60 | 11.65 | 1.87 | 11.06 | 2.23 | 7.16 | 3.12E-02 | 211.7 | down |
| A_23_P32629 | HS6ST3 | 3.59 | 4.89 | 3.10 | 5.64 | 4.07 | 5.68 | 3.97E-02 | 3.5 | down |
| A_33_P3340040 | GINS4 | 5.75 | 3.41 | 4.73 | 1.98 | 4.87 | 3.48 | 3.27E-02 | 4.5 | up |
| A_24_P151692 | POF1B | 9.41 | 8.41 | 9.47 | 8.58 | 9.69 | 7.99 | 4.29E-02 | 2.3 | up |
| A_33_P3307267 | VWF | 3.69 | 2.08 | 2.68 | 1.76 | 3.04 | 2.06 | 3.38E-02 | 2.2 | up |
| A_23_P50108 | NDC80 | 7.05 | 4.81 | 6.33 | 4.46 | 6.81 | 5.45 | 1.91E-02 | 3.5 | up |
| A_23_P110288 | GSTCD | 6.53 | 5.15 | 4.89 | 3.91 | 6.00 | 4.79 | 9.26E-03 | 2.3 | up |
| A_23_P146644 | ANXA2 | 12.91 | 12.26 | 13.25 | 11.91 | 13.96 | 12.94 | 3.82E-02 | 2.0 | up |
| A_33_P3298387 | PLK1 | 8.23 | 6.58 | 7.72 | 6.41 | 7.77 | 6.99 | 3.85E-02 | 2.4 | up |
| A_33_P3285945 | CCL21 | 5.97 | 8.40 | 7.37 | 8.54 | 9.70 | 11.22 | 4.57E-02 | 3.3 | down |
| A_33_P3369885 | MROH1 | 6.02 | 4.09 | 5.32 | 4.42 | 5.14 | 3.81 | 4.40E-02 | 2.6 | up |
| A_33_P3258612 | PCNA | 9.11 | 8.18 | 9.66 | 8.13 | 9.19 | 8.40 | 4.11E-02 | 2.1 | up |
| A_23_P3038 | GPX2 | 13.37 | 12.28 | 13.06 | 11.83 | 13.30 | 12.36 | 5.93E-03 | 2.1 | up |
| A_23_P94103 | SCARA5 | 4.25 | 7.45 | 5.81 | 8.84 | 6.42 | 9.65 | 3.97E-04 | 8.9 | down |
| A_23_P99275 | KLRB1 | 5.05 | 6.96 | 5.10 | 6.02 | 6.77 | 8.21 | 3.84E-02 | 2.7 | down |
| A_21_P0011476 | CHEK2 | 5.90 | 4.92 | 6.40 | 4.57 | 5.53 | 4.44 | 3.99E-02 | 2.5 | up |
| A_24_P200427 | PAICS | 8.46 | 6.94 | 8.02 | 6.85 | 7.76 | 6.80 | 1.80E-02 | 2.3 | up |
| A_23_P120125 | COLEC11 | 2.53 | 3.85 | 1.99 | 4.09 | 3.99 | 6.01 | 1.84E-02 | 3.5 | down |
| A_23_P70448 | HIST1H1A | 5.93 | 4.37 | 6.31 | 5.26 | 6.74 | 5.76 | 2.25E-02 | 2.3 | up |
| A_23_P161135 | LEPR | 2.57 | 4.81 | 3.53 | 5.88 | 3.20 | 4.58 | 2.32E-02 | 4.0 | down |
| A_23_P311901 | ATP10B | 8.62 | 5.67 | 8.72 | 6.33 | 8.29 | 4.87 | 1.02E-02 | 7.6 | up |
| A_23_P308136 | TRIM50 | 4.97 | 8.98 | 4.45 | 8.83 | 4.45 | 7.04 | 2.13E-02 | 12.6 | down |
| A_23_P114259 | ASB11 | 2.14 | 4.65 | 1.89 | 4.24 | 1.81 | 3.52 | 1.21E-02 | 4.6 | down |
| A_24_P244699 | NUDT15 | 6.95 | 5.70 | 6.51 | 5.56 | 6.16 | 5.15 | 7.33E-03 | 2.1 | up |
| A_23_P15450 | TMEM100 | 3.52 | 5.06 | 4.05 | 5.23 | 4.83 | 6.40 | 8.08E-03 | 2.7 | down |
| A_23_P23292 | RXRG | 2.13 | 3.89 | 2.23 | 4.16 | 2.40 | 4.64 | 5.00E-03 | 3.9 | down |
| A_23_P107173 | MEOX1 | 3.43 | 2.09 | 4.61 | 2.14 | 4.26 | 2.62 | 3.32E-02 | 3.5 | up |
| A_32_P34003 | FIGN | 1.77 | 3.83 | 3.40 | 4.27 | 2.90 | 4.54 | 4.94E-02 | 2.9 | down |
| A_23_P149200 | CDC20 | 8.95 | 7.44 | 8.20 | 7.46 | 8.86 | 7.94 | 4.50E-02 | 2.1 | up |
| A_33_P3901921 | PARPBP | 5.84 | 4.51 | 4.96 | 3.80 | 5.44 | 4.14 | 1.75E-03 | 2.4 | up |
| A_23_P38732 | CDH2 | 2.70 | 5.85 | 1.94 | 6.29 | 1.96 | 4.13 | 3.62E-02 | 9.4 | down |
| A_33_P3252369 | TMEM182 | 3.62 | 1.80 | 2.73 | 1.83 | 2.99 | 1.80 | 4.07E-02 | 2.5 | up |
| A_23_P350698 | ANKFN1 | 2.15 | 4.30 | 1.85 | 4.44 | 3.19 | 4.45 | 3.62E-02 | 4.0 | down |
| A_23_P78782 | CA11 | 5.06 | 6.64 | 5.97 | 6.82 | 5.35 | 6.31 | 3.87E-02 | 2.2 | down |
| A_23_P163481 | BUB1B | 7.21 | 5.36 | 6.52 | 5.23 | 7.08 | 5.75 | 1.45E-02 | 2.8 | up |
| A_33_P3377691 | C4orf46 | 5.23 | 3.87 | 4.47 | 3.62 | 4.69 | 3.82 | 2.53E-02 | 2.0 | up |
| A_32_P1173 | MB21D1 | 7.39 | 6.06 | 6.63 | 5.68 | 7.07 | 6.29 | 2.54E-02 | 2.0 | up |
| A_23_P65757 | CCNB2 | 5.48 | 3.44 | 4.32 | 3.43 | 5.45 | 4.04 | 4.88E-02 | 2.7 | up |
| A_24_P165864 | P2RY14 | 3.17 | 5.46 | 3.63 | 5.76 | 4.10 | 6.47 | 1.01E-03 | 4.8 | down |
| A_23_P250385 | HIST1H1B | 10.43 | 8.34 | 10.02 | 8.10 | 10.30 | 9.03 | 1.92E-02 | 3.4 | up |
| A_33_P3347872 | LOC100128727 | 2.66 | 1.92 | 3.93 | 2.76 | 3.51 | 2.02 | 3.46E-02 | 2.2 | up |
| A_33_P3281028 | MACROD2 | 2.20 | 5.36 | 2.34 | 4.68 | 3.54 | 5.13 | 3.49E-02 | 5.2 | down |
| A_23_P331670 | PYGB | 10.09 | 7.63 | 9.38 | 7.64 | 8.54 | 7.48 | 4.92E-02 | 3.4 | up |
| A_23_P157914 | MAMDC2 | 2.97 | 6.56 | 5.29 | 7.64 | 4.78 | 7.57 | 1.50E-02 | 7.5 | down |
| A_33_P3728979 | FAM151B | 3.82 | 2.84 | 4.61 | 2.79 | 3.68 | 2.01 | 2.92E-02 | 2.8 | up |
| A_33_P3347201 | VSTM2A | 2.94 | 5.02 | 2.47 | 5.67 | 4.67 | 6.26 | 4.07E-02 | 4.9 | down |
| A_23_P93282 | HIST1H3J | 11.18 | 8.36 | 9.92 | 7.89 | 10.47 | 9.01 | 3.31E-02 | 4.3 | up |
| A_23_P215132 | WDR91 | 6.59 | 8.22 | 7.31 | 8.46 | 6.97 | 7.91 | 2.66E-02 | 2.4 | down |
| A_23_P104073 | S100A3 | 3.33 | 2.29 | 3.73 | 2.25 | 3.40 | 2.49 | 2.26E-02 | 2.2 | up |
| A_23_P344594 | TMEM174 | 5.47 | 6.55 | 4.99 | 5.71 | 4.92 | 6.33 | 3.33E-02 | 2.1 | down |
| A_23_P203540 | EHF | 9.77 | 8.29 | 8.42 | 7.64 | 9.01 | 7.78 | 2.95E-02 | 2.2 | up |
| A_23_P70991 | AIMP2 | 7.73 | 6.18 | 7.65 | 6.30 | 7.19 | 6.23 | 1.73E-02 | 2.4 | up |
| A_33_P3358824 | PLOD1 | 7.76 | 6.30 | 7.53 | 6.66 | 7.95 | 6.71 | 1.93E-02 | 2.3 | up |
| A_24_P385134 | SCD5 | 2.77 | 4.81 | 4.18 | 5.73 | 3.30 | 5.44 | 8.82E-03 | 3.8 | down |
| A_24_P206328 | PDE1C | 4.45 | 5.76 | 4.30 | 5.57 | 5.33 | 6.90 | 4.70E-03 | 2.6 | down |
| A_23_P363174 | HIST1H2AL | 9.45 | 6.98 | 8.99 | 7.15 | 9.27 | 8.15 | 4.29E-02 | 3.5 | up |
| A_23_P25873 | WDHD1 | 6.35 | 5.07 | 5.61 | 4.88 | 5.92 | 4.70 | 2.61E-02 | 2.1 | up |
| A_23_P111037 | HIST1H3A | 9.99 | 8.99 | 10.27 | 8.71 | 10.08 | 9.24 | 3.49E-02 | 2.2 | up |
| A_33_P3235410 | PTPLA | 3.61 | 4.92 | 3.85 | 5.42 | 3.80 | 4.46 | 4.86E-02 | 2.3 | down |
| A_24_P860797 | PAIP2B | 5.30 | 7.96 | 6.31 | 8.51 | 5.06 | 7.51 | 3.02E-03 | 5.4 | down |
| A_23_P345707 | TICRR | 5.94 | 3.78 | 5.03 | 3.30 | 5.47 | 3.48 | 4.10E-03 | 3.9 | up |
| A_33_P3413114 | ADAMTSL1 | 4.31 | 5.90 | 4.39 | 5.70 | 4.43 | 6.61 | 2.22E-02 | 3.2 | down |
| A_23_P5200 | NPHS1 | 4.64 | 7.36 | 4.94 | 9.49 | 3.17 | 6.73 | 2.08E-02 | 12.2 | down |
| A_24_P74932 | PLP2 | 11.51 | 9.93 | 10.56 | 9.85 | 11.16 | 9.96 | 4.39E-02 | 2.2 | up |
| A_23_P55270 | CCL18 | 6.13 | 3.28 | 5.17 | 1.93 | 6.30 | 4.61 | 3.05E-02 | 6.0 | up |
| A_23_P218827 | POLQ | 6.04 | 5.07 | 5.57 | 4.15 | 6.16 | 5.14 | 1.54E-02 | 2.2 | up |
| A_32_P96719 | SHCBP1 | 5.39 | 2.73 | 4.69 | 1.91 | 5.40 | 2.60 | 2.61E-04 | 6.7 | up |
| A_24_P71468 | QPCT | 4.10 | 1.84 | 4.49 | 1.94 | 5.30 | 3.58 | 1.23E-02 | 4.5 | up |
| A_23_P8640 | GPER1 | 1.99 | 8.40 | 4.59 | 9.33 | 4.46 | 8.95 | 1.32E-02 | 37.1 | down |
| A_23_P375 | CDCA8 | 8.80 | 7.28 | 8.11 | 6.94 | 8.49 | 7.51 | 1.64E-02 | 2.3 | up |
| A_32_P105549 | ANXA8L1 | 2.56 | 1.92 | 3.37 | 2.14 | 3.22 | 1.93 | 3.68E-02 | 2.1 | up |
| A_23_P251421 | CDCA7 | 6.03 | 2.90 | 6.56 | 1.95 | 5.94 | 3.44 | 3.19E-02 | 10.7 | up |
| A_23_P50990 | CENPO | 6.98 | 5.79 | 6.61 | 5.42 | 6.78 | 5.64 | 2.33E-04 | 2.3 | up |
| A_33_P3302025 | ADAMTSL1 | 4.65 | 7.45 | 5.72 | 6.98 | 5.84 | 8.12 | 4.32E-02 | 4.3 | down |
| A_33_P3357199 | SH2D6 | 1.99 | 5.38 | 3.63 | 6.71 | 2.89 | 4.97 | 1.86E-02 | 7.2 | down |
| A_24_P70906 | PDILT | 1.95 | 4.06 | 1.69 | 4.56 | 1.78 | 4.46 | 7.74E-03 | 5.9 | down |
| A_24_P205589 | ACOT7 | 8.65 | 6.74 | 8.37 | 7.13 | 8.47 | 7.28 | 2.56E-02 | 2.7 | up |
| A_24_P62783 | FABP3 | 4.88 | 6.69 | 5.78 | 7.84 | 4.11 | 5.18 | 3.14E-02 | 3.1 | down |
| A_23_P75071 | KIF20B | 6.06 | 4.95 | 6.18 | 4.01 | 6.45 | 5.23 | 4.61E-02 | 2.8 | up |
| A_23_P415411 | HIST1H4E | 11.68 | 10.64 | 11.35 | 10.08 | 11.33 | 10.50 | 1.41E-02 | 2.1 | up |
| A_23_P133123 | MND1 | 5.84 | 4.16 | 4.22 | 2.17 | 5.06 | 3.25 | 3.39E-03 | 3.6 | up |
| A_23_P24433 | CTSF | 6.47 | 7.86 | 7.08 | 7.77 | 6.85 | 7.94 | 3.60E-02 | 2.1 | down |
| A_19_P00812553 | PPA1 | 8.53 | 7.54 | 8.58 | 7.48 | 8.76 | 7.64 | 1.54E-03 | 2.1 | up |
| A_33_P3346444 | PCDH11Y | 2.88 | 5.05 | 2.04 | 4.40 | 3.55 | 6.03 | 1.45E-03 | 5.1 | down |
| A_24_P223384 | HIST1H2AB | 9.75 | 7.11 | 8.38 | 6.67 | 8.37 | 7.16 | 4.75E-02 | 3.6 | up |
| A_33_P3319041 | HMGB3 | 5.59 | 3.87 | 4.90 | 3.12 | 4.69 | 3.61 | 2.08E-02 | 2.9 | up |
| A_33_P3236568 | LCE2D | 2.37 | 3.27 | 2.58 | 3.56 | 1.83 | 2.99 | 5.58E-03 | 2.0 | down |
| A_24_P365506 | FERMT1 | 8.53 | 6.33 | 7.38 | 6.24 | 8.17 | 6.07 | 3.33E-02 | 3.5 | up |
| A_24_P7965 | ESRRG | 7.68 | 10.81 | 6.46 | 9.83 | 6.75 | 9.22 | 8.02E-03 | 7.9 | down |
| A_33_P3220207 | ARMC3 | 1.99 | 3.34 | 2.50 | 3.60 | 1.74 | 3.64 | 2.57E-02 | 2.7 | down |
| A_24_P160466 | GPRIN1 | 5.83 | 4.16 | 5.26 | 4.12 | 6.03 | 4.60 | 1.17E-02 | 2.7 | up |
| A_24_P231104 | LEPR | 2.33 | 6.64 | 4.74 | 7.82 | 1.82 | 5.74 | 9.29E-03 | 13.7 | down |
| A_23_P81825 | GUCA1B | 4.39 | 3.19 | 3.92 | 1.75 | 3.89 | 2.55 | 3.49E-02 | 3.0 | up |
| A_33_P3831099 | LOC401433 | 5.12 | 3.27 | 3.26 | 1.75 | 4.23 | 1.75 | 2.05E-02 | 3.9 | up |
| A_33_P3360426 | WDR1 | 10.59 | 9.60 | 10.62 | 9.23 | 10.87 | 10.10 | 2.85E-02 | 2.1 | up |
| A_23_P105144 | SCUBE2 | 3.85 | 6.60 | 4.71 | 7.65 | 4.94 | 6.59 | 2.57E-02 | 5.5 | down |
| A_33_P3268507 | CEACAM1 | 11.03 | 8.79 | 11.47 | 8.41 | 11.59 | 7.91 | 1.87E-02 | 8.0 | up |
| A_23_P214459 | PRPH2 | 3.16 | 4.66 | 5.12 | 6.38 | 4.36 | 5.17 | 2.76E-02 | 2.3 | down |
| A_23_P253524 | CENPE | 6.23 | 4.27 | 5.50 | 4.19 | 6.30 | 4.84 | 1.49E-02 | 3.0 | up |
| A_23_P7957 | GNMT | 4.59 | 7.18 | 3.66 | 6.86 | 4.64 | 6.13 | 3.99E-02 | 5.4 | down |
| A_24_P225970 | SGOL1 | 5.80 | 4.57 | 5.11 | 3.77 | 5.72 | 3.89 | 1.57E-02 | 2.8 | up |
| A_23_P52974 | GIF | 1.99 | 15.06 | 1.97 | 13.75 | 2.44 | 12.81 | 4.37E-03 | 3426.1 | down |
| A_32_P224234 | LOC645195 | 1.80 | 4.25 | 1.70 | 4.38 | 2.45 | 3.81 | 3.41E-02 | 4.5 | down |
| A_23_P210109 | CYP26B1 | 5.22 | 3.72 | 6.94 | 4.56 | 5.84 | 3.89 | 1.64E-02 | 3.8 | up |
| A_23_P35684 | INPP5F | 5.89 | 4.94 | 6.01 | 4.56 | 5.99 | 5.30 | 4.28E-02 | 2.0 | up |
| A_23_P153524 | C19orf73 | 4.83 | 3.23 | 5.13 | 3.65 | 3.19 | 1.77 | 1.22E-03 | 2.8 | up |
| A_33_P3266923 | LMTK2 | 9.02 | 7.80 | 9.57 | 7.83 | 8.24 | 7.46 | 4.59E-02 | 2.4 | up |
| A_33_P3310009 | DCAF8L2 | 2.10 | 3.55 | 4.32 | 5.01 | 2.73 | 4.14 | 4.10E-02 | 2.3 | down |
| A_23_P121374 | SEMA5B | 5.59 | 3.70 | 5.91 | 3.52 | 6.09 | 4.79 | 2.76E-02 | 3.6 | up |
| A_24_P330518 | CA12 | 5.25 | 7.11 | 8.18 | 9.24 | 6.04 | 7.56 | 2.36E-02 | 2.8 | down |
| A_33_P3337599 | PRKDC | 7.16 | 6.15 | 7.14 | 5.91 | 6.88 | 5.76 | 3.42E-03 | 2.2 | up |
| A_23_P121222 | RAD18 | 3.99 | 2.30 | 3.17 | 2.25 | 3.69 | 2.23 | 2.73E-02 | 2.6 | up |
| A_23_P57588 | GTSE1 | 7.09 | 4.36 | 6.40 | 4.45 | 7.01 | 5.44 | 2.58E-02 | 4.2 | up |
| A_23_P103486 | CYP2J2 | 6.95 | 5.69 | 6.18 | 5.35 | 6.41 | 5.21 | 1.52E-02 | 2.1 | up |
| A_24_P217834 | HIST1H3D | 12.95 | 10.57 | 11.85 | 9.94 | 12.23 | 11.20 | 4.65E-02 | 3.4 | up |
| A_23_P150549 | PGA3 | 4.72 | 18.88 | 4.35 | 18.99 | 6.04 | 18.81 | 1.65E-03 | 14821.1 | down |
| A_23_P429977 | KCNQ1 | 6.38 | 9.07 | 5.99 | 8.18 | 5.65 | 6.82 | 4.60E-02 | 4.0 | down |
| A_23_P372988 | PRR22 | 4.69 | 3.95 | 3.06 | 1.70 | 3.78 | 2.84 | 3.18E-02 | 2.0 | up |
| A_23_P70445 | HIST1H3E | 11.92 | 9.60 | 11.25 | 9.48 | 11.54 | 10.48 | 4.24E-02 | 3.3 | up |
| A_33_P3374205 | MKI67 | 9.73 | 6.72 | 8.97 | 6.52 | 8.65 | 7.31 | 4.40E-02 | 4.8 | up |
| A_23_P400505 | C1QTNF9 | 1.75 | 3.61 | 2.01 | 3.45 | 2.03 | 4.18 | 1.26E-02 | 3.5 | down |
| A_24_P695306 | TMEM229A | 3.46 | 6.61 | 3.23 | 5.96 | 1.73 | 4.70 | 1.66E-03 | 7.7 | down |
| A_23_P12241 | MCOLN3 | 1.96 | 4.76 | 3.11 | 6.11 | 2.81 | 5.26 | 3.48E-03 | 6.7 | down |
| A_33_P3212994 | ZWINT | 7.03 | 5.27 | 5.97 | 5.18 | 6.54 | 5.20 | 4.38E-02 | 2.5 | up |
| A_23_P118741 | SLC16A13 | 6.02 | 4.62 | 5.56 | 4.76 | 6.21 | 5.02 | 2.36E-02 | 2.2 | up |
| A_24_P55148 | HIST1H2BJ | 13.52 | 11.69 | 12.83 | 11.14 | 12.86 | 12.03 | 4.40E-02 | 2.7 | up |
| A_23_P314672 | C11orf40 | 1.77 | 5.55 | 1.68 | 7.14 | 1.66 | 5.69 | 1.36E-02 | 21.5 | down |
| A_23_P117797 | CLN6 | 8.25 | 6.79 | 7.36 | 6.45 | 7.48 | 6.74 | 4.01E-02 | 2.0 | up |
| A_24_P99090 | CKAP2 | 6.31 | 4.04 | 6.26 | 3.66 | 5.89 | 4.74 | 4.43E-02 | 4.0 | up |
| A_23_P63402 | GPSM2 | 7.04 | 4.93 | 6.54 | 5.25 | 6.91 | 5.74 | 3.59E-02 | 2.9 | up |
| A_33_P3398526 | BCL2L11 | 9.38 | 12.13 | 8.75 | 12.98 | 9.03 | 11.53 | 2.79E-02 | 9.0 | down |
| A_33_P3272744 | RHBG | 4.73 | 2.81 | 3.54 | 1.71 | 3.19 | 1.66 | 4.42E-03 | 3.4 | up |
| A_33_P3345812 | GPER1 | 7.33 | 8.81 | 6.81 | 9.48 | 7.11 | 9.54 | 2.62E-02 | 4.6 | down |
| A_33_P3300232 | HYDIN | 2.28 | 3.39 | 3.49 | 5.08 | 1.79 | 3.84 | 2.75E-02 | 3.0 | down |
| A_24_P664850 | ST8SIA2 | 4.01 | 3.09 | 3.18 | 1.92 | 3.86 | 2.23 | 2.48E-02 | 2.4 | up |
| A_24_P397386 | LIFR | 4.71 | 8.73 | 4.43 | 8.03 | 5.08 | 7.01 | 3.82E-02 | 9.1 | down |
| A_24_P419132 | CENPI | 5.92 | 2.53 | 4.90 | 1.69 | 5.14 | 3.30 | 2.92E-02 | 7.0 | up |
| A_23_P403081 | C5orf34 | 3.29 | 1.71 | 3.69 | 2.41 | 3.38 | 1.67 | 6.85E-03 | 2.9 | up |
| A_23_P66241 | MT1M | 6.17 | 8.51 | 4.21 | 8.94 | 4.45 | 8.14 | 3.52E-02 | 12.0 | down |
| A_23_P23873 | PAPPA2 | 1.72 | 2.79 | 1.64 | 2.40 | 2.18 | 3.53 | 2.45E-02 | 2.1 | down |
| A_23_P143190 | MYBL2 | 8.51 | 7.43 | 8.69 | 7.51 | 8.26 | 7.43 | 1.06E-02 | 2.0 | up |
| A_33_P3386760 | CHEK2 | 5.85 | 3.44 | 5.80 | 3.79 | 5.55 | 4.35 | 3.49E-02 | 3.7 | up |
| A_23_P140450 | SLC27A2 | 6.54 | 4.60 | 6.92 | 3.58 | 6.26 | 4.47 | 4.13E-02 | 5.1 | up |
| A_24_P350759 | SLC1A2 | 1.71 | 5.93 | 2.34 | 6.84 | 3.64 | 5.73 | 4.17E-02 | 12.1 | down |
| A_23_P95453 | NRXN1 | 1.73 | 3.23 | 1.62 | 3.40 | 2.55 | 4.12 | 2.81E-03 | 3.1 | down |
| A_32_P225355 | CPEB2 | 3.29 | 5.55 | 3.72 | 5.24 | 3.91 | 4.92 | 4.78E-02 | 3.0 | down |
| A_24_P115932 | PTGDR2 | 3.28 | 6.35 | 3.25 | 5.77 | 5.35 | 6.82 | 3.76E-02 | 5.1 | down |
| A_23_P429491 | DDIAS | 4.94 | 3.94 | 3.83 | 2.61 | 4.49 | 3.67 | 1.33E-02 | 2.0 | up |
| A_23_P47565 | LDHA | 12.44 | 11.07 | 12.53 | 10.87 | 12.96 | 11.98 | 2.07E-02 | 2.5 | up |
| A_33_P3326210 | ESCO2 | 8.00 | 5.99 | 6.91 | 5.77 | 7.48 | 5.41 | 2.90E-02 | 3.3 | up |
| A_23_P204947 | GJB2 | 6.20 | 3.87 | 7.51 | 4.01 | 6.60 | 4.68 | 3.22E-02 | 6.0 | up |
| A_24_P296254 | ARHGAP11A | 3.63 | 1.69 | 3.29 | 2.41 | 3.58 | 1.78 | 4.38E-02 | 2.9 | up |
| A_24_P360206 | PCDHA11 | 1.76 | 3.81 | 3.55 | 4.80 | 4.53 | 5.84 | 2.69E-02 | 2.9 | down |
| A_23_P147918 | S100A16 | 11.70 | 10.25 | 10.85 | 10.11 | 11.98 | 10.51 | 3.73E-02 | 2.3 | up |
| A_23_P390518 | TNFRSF11A | 8.69 | 6.28 | 7.70 | 5.79 | 7.17 | 5.62 | 1.60E-02 | 3.9 | up |
| A_23_P15582 | XYLT2 | 5.05 | 8.05 | 5.48 | 8.04 | 5.60 | 7.36 | 2.11E-02 | 5.4 | down |
| A_23_P88691 | CHRNA5 | 6.31 | 3.35 | 5.49 | 2.79 | 5.53 | 3.69 | 1.76E-02 | 5.7 | up |
| A_23_P94296 | ADAM7 | 3.39 | 1.72 | 2.67 | 1.65 | 2.77 | 1.61 | 2.26E-02 | 2.4 | up |
| A_23_P64919 | RERGL | 1.68 | 4.71 | 2.68 | 4.17 | 2.56 | 5.43 | 3.71E-02 | 5.5 | down |
| A_24_P193592 | CCNF | 4.67 | 2.30 | 4.48 | 1.82 | 4.66 | 3.50 | 4.59E-02 | 4.2 | up |
| A_24_P19228 | GAMT | 5.77 | 7.82 | 6.14 | 7.82 | 6.52 | 7.51 | 3.66E-02 | 3.0 | down |
| A_24_P207995 | L1CAM | 3.09 | 4.70 | 3.88 | 6.16 | 4.62 | 6.82 | 1.08E-02 | 4.1 | down |
| A_33_P3343737 | CELA3A | 3.73 | 5.70 | 2.09 | 3.56 | 3.08 | 3.97 | 4.36E-02 | 2.7 | down |
| A_33_P3290567 | WEE1 | 1.75 | 3.42 | 1.94 | 3.39 | 1.67 | 3.05 | 3.48E-03 | 2.8 | down |
| A_23_P362148 | DNER | 1.68 | 7.16 | 1.62 | 7.25 | 1.59 | 4.60 | 3.13E-02 | 26.1 | down |
| A_24_P227091 | KIF11 | 8.90 | 6.86 | 8.16 | 6.39 | 8.02 | 7.05 | 3.80E-02 | 3.0 | up |
| A_23_P114423 | RGN | 1.71 | 4.74 | 3.16 | 5.48 | 2.19 | 4.20 | 1.48E-02 | 5.5 | down |
| A_24_P56363 | CAB39L | 4.51 | 6.93 | 5.90 | 7.73 | 5.58 | 7.44 | 8.89E-03 | 4.1 | down |
| A_33_P3344127 | HIST1H2AC | 10.80 | 8.95 | 9.96 | 7.95 | 10.15 | 9.18 | 3.80E-02 | 3.1 | up |
| A_23_P138328 | PM20D1 | 1.73 | 5.08 | 2.06 | 6.37 | 1.95 | 3.99 | 3.89E-02 | 9.4 | down |
| A_33_P3308949 | DBT | 3.50 | 4.44 | 3.39 | 4.66 | 2.75 | 3.74 | 9.71E-03 | 2.1 | down |
| A_33_P3258660 | SCD5 | 7.17 | 7.98 | 7.64 | 8.63 | 7.22 | 8.67 | 2.92E-02 | 2.1 | down |
| A_33_P3217393 | CD276 | 8.48 | 7.48 | 8.90 | 7.80 | 8.56 | 7.63 | 2.40E-03 | 2.0 | up |
| A_23_P69310 | CCRL2 | 6.56 | 4.18 | 4.73 | 2.35 | 6.38 | 3.57 | 3.11E-03 | 5.7 | up |
| A_24_P108311 | NEDD4L | 6.65 | 8.03 | 6.09 | 7.73 | 5.64 | 6.74 | 1.30E-02 | 2.6 | down |
| A_32_P54274 | DRD5 | 7.21 | 9.34 | 6.26 | 8.41 | 6.13 | 7.17 | 4.03E-02 | 3.4 | down |
| A_33_P3336657 | PLEKHM3 | 5.61 | 7.10 | 6.26 | 8.17 | 6.16 | 7.03 | 4.23E-02 | 2.7 | down |
| A_33_P3385785 | S100A12 | 4.77 | 3.25 | 4.31 | 3.28 | 5.01 | 3.98 | 1.81E-02 | 2.3 | up |
| A_21_P0000151 | ASB11 | 4.08 | 7.18 | 2.24 | 7.08 | 2.84 | 5.62 | 3.07E-02 | 11.9 | down |
| A_33_P3303542 | SSC5D | 6.98 | 11.87 | 8.19 | 12.95 | 6.56 | 11.17 | 2.82E-04 | 27.0 | down |
| A_33_P3335910 | SYNE1 | 3.26 | 7.22 | 2.93 | 5.78 | 3.52 | 5.78 | 2.60E-02 | 8.1 | down |
| A_24_P346855 | MKI67 | 6.52 | 4.27 | 6.19 | 4.42 | 6.77 | 4.71 | 4.74E-03 | 4.1 | up |
| A_23_P152428 | MARVELD3 | 5.41 | 2.64 | 5.80 | 2.58 | 3.87 | 1.58 | 9.58E-03 | 6.8 | up |
| A_33_P3263232 | LRRC3 | 3.71 | 1.58 | 5.14 | 2.33 | 4.77 | 1.58 | 1.27E-02 | 6.6 | up |
| A_33_P3306146 | PLAU | 9.09 | 5.79 | 9.26 | 5.70 | 8.74 | 7.02 | 3.82E-02 | 7.3 | up |
| A_33_P3345534 | KRT14 | 3.97 | 2.71 | 4.88 | 3.49 | 4.59 | 3.12 | 1.94E-03 | 2.6 | up |
| A_23_P401 | CENPF | 7.47 | 5.70 | 6.91 | 5.42 | 7.30 | 6.01 | 8.57E-03 | 2.9 | up |
| A_33_P3745164 | CADM2 | 1.60 | 3.10 | 1.57 | 2.25 | 1.57 | 3.14 | 4.77E-02 | 2.4 | down |
| A_23_P63789 | ZWINT | 7.72 | 6.60 | 7.21 | 6.16 | 7.85 | 6.30 | 1.61E-02 | 2.4 | up |
| A_21_P0011475 | CHEK2 | 6.38 | 5.13 | 6.78 | 5.25 | 6.19 | 5.39 | 3.02E-02 | 2.3 | up |
| A_19_P00806947 | PCNA | 9.33 | 8.08 | 9.59 | 8.08 | 9.12 | 8.46 | 4.58E-02 | 2.2 | up |
| A_23_P204879 | CAB39L | 4.47 | 6.83 | 6.14 | 7.65 | 5.81 | 7.41 | 2.13E-02 | 3.5 | down |
| A_23_P161338 | PPA1 | 8.70 | 7.81 | 8.65 | 7.54 | 9.23 | 7.99 | 8.61E-03 | 2.1 | up |
| A_23_P28246 | SLC23A3 | 4.37 | 2.78 | 3.55 | 2.65 | 4.81 | 3.98 | 4.57E-02 | 2.2 | up |
| A_32_P201773 | AMMECR1 | 6.74 | 4.71 | 5.90 | 4.49 | 6.54 | 4.43 | 1.37E-02 | 3.6 | up |
| A_23_P167683 | GDNF | 1.53 | 3.98 | 2.80 | 4.40 | 4.10 | 5.23 | 4.68E-02 | 3.3 | down |
| A_33_P3253501 | HIST2H2BF | 13.60 | 11.95 | 12.91 | 11.46 | 13.05 | 12.35 | 4.87E-02 | 2.4 | up |
| A_33_P3331853 | PDE1C | 3.45 | 5.61 | 3.52 | 5.19 | 4.82 | 6.30 | 1.26E-02 | 3.4 | down |
| A_23_P411335 | SGOL2 | 5.82 | 4.17 | 5.02 | 3.18 | 6.07 | 4.72 | 7.98E-03 | 3.1 | up |
| A_33_P3240693 | THSD4 | 1.57 | 6.67 | 2.58 | 5.91 | 1.56 | 5.12 | 1.86E-02 | 16.0 | down |
| A_32_P397824 | FIGN | 1.56 | 3.86 | 3.67 | 4.77 | 3.01 | 4.87 | 3.77E-02 | 3.4 | down |
| A_24_P357726 | PSG8 | 4.78 | 2.60 | 4.74 | 3.49 | 4.86 | 3.24 | 2.48E-02 | 3.2 | up |
| A_33_P3339361 | ARHGAP11A | 5.12 | 2.54 | 4.41 | 1.58 | 4.62 | 2.37 | 4.30E-03 | 5.9 | up |
| A_24_P356509 | RAD51D | 4.42 | 3.38 | 4.56 | 3.26 | 4.38 | 3.00 | 6.72E-03 | 2.4 | up |
| A_23_P55616 | SLC14A1 | 3.58 | 5.47 | 3.57 | 4.51 | 2.92 | 4.91 | 4.13E-02 | 3.0 | down |
| A_33_P3618960 | P2RX6 | 1.60 | 3.61 | 2.30 | 3.68 | 1.59 | 2.57 | 4.02E-02 | 2.7 | down |
| A_23_P375372 | FGA | 1.61 | 9.96 | 2.80 | 8.98 | 1.60 | 7.75 | 1.10E-02 | 119.0 | down |
| A_24_P214231 | STIL | 6.20 | 3.11 | 4.30 | 1.64 | 5.19 | 3.87 | 4.81E-02 | 5.1 | up |
| A_32_P44568 | LDHA | 10.71 | 9.32 | 10.58 | 9.06 | 10.92 | 9.71 | 3.92E-03 | 2.6 | up |
| A_33_P3273669 | NRXN1 | 1.70 | 4.23 | 3.11 | 4.71 | 3.00 | 6.03 | 2.98E-02 | 5.2 | down |
| A_23_P418785 | STXBP5L | 1.64 | 2.72 | 2.10 | 3.12 | 2.11 | 3.35 | 3.22E-03 | 2.2 | down |
| A_21_P0013574 | MTHFD1L | 7.69 | 5.74 | 7.58 | 5.48 | 6.86 | 5.89 | 4.22E-02 | 3.2 | up |
| A_33_P3221111 | PGA3 | 7.63 | 11.04 | 7.09 | 11.57 | 7.07 | 10.46 | 8.97E-03 | 13.5 | down |
| A_23_P118174 | PLK1 | 9.38 | 7.40 | 8.89 | 7.22 | 8.91 | 7.79 | 2.38E-02 | 3.0 | up |
| A_23_P211522 | SYNGR1 | 9.22 | 10.44 | 8.48 | 9.98 | 8.75 | 9.49 | 3.58E-02 | 2.2 | down |
| A_32_P88240 | KBTBD12 | 1.71 | 3.32 | 1.65 | 3.36 | 1.68 | 2.46 | 4.40E-02 | 2.6 | down |
| A_24_P323598 | ESCO2 | 5.69 | 4.29 | 4.86 | 3.83 | 5.84 | 4.00 | 2.65E-02 | 2.7 | up |
| A_33_P3365228 | SEMA5B | 5.42 | 3.87 | 5.47 | 3.11 | 5.86 | 4.39 | 2.39E-02 | 3.5 | up |
| A_33_P3420757 | AQP4 | 3.49 | 9.22 | 3.68 | 8.86 | 3.47 | 6.39 | 3.30E-02 | 24.4 | down |
| A_33_P3293748 | TRIM50 | 1.76 | 7.64 | 1.68 | 7.30 | 1.72 | 4.76 | 3.34E-02 | 28.7 | down |
| A_33_P3345816 | GPER1 | 4.22 | 8.09 | 3.34 | 9.00 | 2.55 | 8.33 | 1.43E-02 | 34.3 | down |
| A_21_P0000121 | C19orf81 | 2.37 | 3.02 | 2.20 | 3.70 | 1.80 | 3.16 | 4.74E-02 | 2.2 | down |
| A_33_P3293187 | SLC14A2 | 4.32 | 6.72 | 4.56 | 8.43 | 3.93 | 6.69 | 2.06E-02 | 8.0 | down |
| A_24_P15754 | TOMM40 | 8.50 | 7.24 | 8.31 | 6.86 | 7.74 | 7.03 | 3.62E-02 | 2.2 | up |
| A_33_P3265359 | HES6 | 6.00 | 4.40 | 6.97 | 5.28 | 5.50 | 3.92 | 4.34E-04 | 3.1 | up |
| A_19_P00807336 | PPA1 | 10.26 | 9.32 | 10.27 | 8.90 | 10.64 | 9.47 | 1.12E-02 | 2.2 | up |
| A_24_P25544 | GDNF | 1.79 | 2.78 | 2.81 | 3.74 | 3.63 | 4.85 | 6.64E-03 | 2.1 | down |
| A_23_P334883 | SHANK2 | 4.45 | 5.73 | 5.02 | 5.85 | 4.00 | 4.88 | 1.98E-02 | 2.0 | down |
| A_33_P3284404 | SYNGR1 | 9.95 | 11.30 | 9.43 | 10.74 | 9.68 | 10.36 | 3.64E-02 | 2.2 | down |
| A_23_P143526 | S100B | 2.55 | 4.15 | 1.80 | 3.73 | 3.16 | 4.93 | 3.01E-03 | 3.4 | down |
| A_33_P3309832 | PLEKHS1 | 6.50 | 3.24 | 4.92 | 1.73 | 4.71 | 2.70 | 2.00E-02 | 7.0 | up |
| Note: GC, gastric cancer; FC(abs), absolute fold change. | | | | | | | | | | |

| Table S4. The correlation of differentially expressed mRNAs with differentially m^6^A-methylated and expressed lncRNAs | | | | | | | | |
| --- | --- | --- | --- | --- | --- | --- | --- | --- |
| mRNAs | RASAL2-AS1 | | LINC00910 | | SNHG7 | | LINC01105 | |
|  | *r* | *P* | *r* | *P* | *r* | *P* | *r* | *P* |
| MT1F | -.828^*^ | 0.042 | -0.796 | 0.058 | -.887^*^ | 0.018 | -.911^*^ | 0.012 |
| ANGPT2 | 0.658 | 0.155 | 0.578 | 0.230 | 0.592 | 0.215 | 0.667 | 0.148 |
| ACAA2 | .885^*^ | 0.019 | 0.631 | 0.179 | 0.748 | 0.087 | .827^*^ | 0.042 |
| ENO1 | .835^*^ | 0.038 | 0.568 | 0.240 | 0.501 | 0.311 | 0.704 | 0.119 |
| PPA1 | .841^*^ | 0.036 | 0.687 | 0.132 | 0.682 | 0.136 | 0.809 | 0.051 |
| KIF15 | .874^*^ | 0.023 | 0.662 | 0.152 | 0.548 | 0.260 | 0.769 | 0.074 |
| TFAP4 | .946^**^ | 0.004 | 0.579 | 0.229 | 0.752 | 0.085 | .833^*^ | 0.039 |
| PDSS1 | .852^*^ | 0.031 | 0.779 | 0.068 | .910^*^ | 0.012 | .921^**^ | 0.009 |
| MTFR2 | .838^*^ | 0.037 | 0.685 | 0.133 | 0.711 | 0.113 | .816^*^ | 0.047 |
| RACGAP1 | .945^**^ | 0.004 | 0.795 | 0.059 | 0.738 | 0.094 | .908^*^ | 0.012 |
| HIST1H1D | .856^*^ | 0.030 | 0.527 | 0.283 | 0.581 | 0.227 | 0.723 | 0.105 |
| FUT6 | .851^*^ | 0.032 | .912^*^ | 0.011 | .909^*^ | 0.012 | .970^**^ | 0.001 |
| NETO2 | .962^**^ | 0.002 | 0.594 | 0.214 | 0.713 | 0.111 | .833^*^ | 0.040 |
| ZNF536 | -0.679 | 0.138 | -0.528 | 0.282 | -0.696 | 0.124 | -0.691 | 0.129 |
| IGF2 | 0.675 | 0.142 | .886^*^ | 0.019 | 0.792 | 0.060 | .852^*^ | 0.031 |
| STRIP2 | .900^*^ | 0.014 | 0.439 | 0.384 | 0.449 | 0.372 | 0.665 | 0.150 |
| PDZD2 | -.849^*^ | 0.033 | -0.234 | 0.655 | -0.398 | 0.434 | -0.553 | 0.256 |
| NMU | .821^*^ | 0.045 | 0.643 | 0.168 | 0.549 | 0.259 | 0.742 | 0.091 |
| FGA | -.819^*^ | 0.046 | -.852^*^ | 0.031 | -.827^*^ | 0.042 | -.908^*^ | 0.012 |
| LINGO2 | -.939^**^ | 0.005 | -0.701 | 0.121 | -0.696 | 0.125 | -.857^*^ | 0.029 |
| ONECUT3 | .919^**^ | 0.009 | 0.678 | 0.139 | 0.568 | 0.239 | 0.800 | 0.056 |
| ZFYVE27 | -.892^*^ | 0.017 | -0.773 | 0.071 | -0.803 | 0.054 | -.900^*^ | 0.014 |
| KRT80 | 0.689 | 0.130 | .938^**^ | 0.006 | .824^*^ | 0.044 | .888^*^ | 0.018 |
| KIAA1958 | -.858^*^ | 0.029 | -0.685 | 0.133 | -0.671 | 0.145 | -0.811 | 0.050 |
| NUF2 | .961^**^ | 0.002 | 0.716 | 0.109 | 0.702 | 0.120 | .874^*^ | 0.023 |
| MUC3A | .880^*^ | 0.021 | 0.784 | 0.065 | .874^*^ | 0.023 | .923^**^ | 0.009 |
| ATP4A | -.820^*^ | 0.046 | -0.788 | 0.063 | -0.735 | 0.096 | -.855^*^ | 0.030 |
| HS6ST3 | -.905^*^ | 0.013 | -0.705 | 0.118 | -.884^*^ | 0.019 | -.907^*^ | 0.013 |
| GINS4 | .900^*^ | 0.015 | 0.514 | 0.297 | 0.568 | 0.240 | 0.731 | 0.099 |
| POF1B | .849^*^ | 0.032 | .897^*^ | 0.015 | .899^*^ | 0.015 | .960^**^ | 0.002 |
| VWF | .945^**^ | 0.004 | 0.589 | 0.219 | 0.542 | 0.267 | 0.768 | 0.074 |
| NDC80 | .882^*^ | 0.020 | 0.677 | 0.140 | 0.618 | 0.191 | 0.801 | 0.056 |
| GSTCD | 0.771 | 0.073 | 0.297 | 0.567 | 0.289 | 0.578 | 0.509 | 0.302 |
| ANXA2 | 0.508 | 0.303 | 0.592 | 0.216 | 0.544 | 0.265 | 0.597 | 0.211 |
| PLK1 | .930^**^ | 0.007 | 0.684 | 0.134 | 0.642 | 0.169 | .830^*^ | 0.041 |
| CCL21 | -0.752 | 0.085 | -0.391 | 0.443 | -0.534 | 0.275 | -0.616 | 0.193 |
| MROH1 | .977^**^ | 0.001 | 0.783 | 0.066 | 0.749 | 0.087 | .920^**^ | 0.009 |
| PCNA | .819^*^ | 0.046 | .816^*^ | 0.048 | .889^*^ | 0.018 | .915^*^ | 0.011 |
| GPX2 | .893^*^ | 0.017 | 0.655 | 0.158 | 0.669 | 0.146 | .813^*^ | 0.049 |
| SCARA5 | -.967^**^ | 0.002 | -0.600 | 0.208 | -0.712 | 0.112 | -.836^*^ | 0.038 |
| KLRB1 | -0.764 | 0.077 | -0.670 | 0.145 | -0.748 | 0.087 | -0.794 | 0.059 |
| CHEK2 | .889^*^ | 0.018 | 0.740 | 0.093 | .913^*^ | 0.011 | .923^**^ | 0.009 |
| PAICS | .997^**^ | 0.000 | 0.733 | 0.098 | 0.755 | 0.083 | .911^*^ | 0.011 |
| COLEC11 | -0.791 | 0.061 | -0.621 | 0.189 | -.830^*^ | 0.041 | -.813^*^ | 0.049 |
| HIST1H1A | 0.560 | 0.248 | .832^*^ | 0.040 | 0.635 | 0.176 | 0.735 | 0.096 |
| LEPR | -.900^*^ | 0.015 | -0.559 | 0.248 | -0.578 | 0.230 | -0.751 | 0.085 |
| ATP10B | .914^*^ | 0.011 | .899^*^ | 0.015 | .915^*^ | 0.011 | .992^**^ | 0.000 |
| TRIM50 | -0.807 | 0.052 | -0.795 | 0.059 | -0.750 | 0.086 | -.857^*^ | 0.029 |
| ASB11 | -.817^*^ | 0.047 | -.846^*^ | 0.034 | -0.775 | 0.070 | -.889^*^ | 0.018 |
| NUDT15 | .981^**^ | 0.001 | 0.686 | 0.132 | 0.759 | 0.080 | .889^*^ | 0.018 |
| TMEM100 | -.912^*^ | 0.011 | -0.631 | 0.179 | -0.749 | 0.086 | -.838^*^ | 0.037 |
| RXRG | -.945^**^ | 0.004 | -0.807 | 0.052 | -.876^*^ | 0.022 | -.957^**^ | 0.003 |
| MEOX1 | 0.720 | 0.107 | .841^*^ | 0.036 | .880^*^ | 0.021 | .882^*^ | 0.020 |
| FIGN | -.942^**^ | 0.005 | -0.557 | 0.251 | -0.538 | 0.271 | -0.754 | 0.083 |
| CDC20 | .832^*^ | 0.040 | 0.669 | 0.146 | 0.517 | 0.294 | 0.745 | 0.089 |
| PARPBP | .922^**^ | 0.009 | 0.563 | 0.245 | 0.585 | 0.222 | 0.764 | 0.077 |
| CDH2 | -0.766 | 0.076 | -0.733 | 0.097 | -0.723 | 0.104 | -0.810 | 0.051 |
| TMEM182 | .971^**^ | 0.001 | 0.722 | 0.106 | 0.652 | 0.160 | .863^*^ | 0.027 |
| ANKFN1 | -.937^**^ | 0.006 | -0.754 | 0.083 | -.856^*^ | 0.030 | -.928^**^ | 0.007 |
| CA11 | -.876^*^ | 0.022 | -0.618 | 0.191 | -0.507 | 0.304 | -0.740 | 0.092 |
| BUB1B | .895^*^ | 0.016 | 0.724 | 0.103 | 0.642 | 0.170 | .831^*^ | 0.040 |
| C4orf46 | .964^**^ | 0.002 | 0.629 | 0.181 | 0.598 | 0.210 | 0.809 | 0.051 |
| MB21D1 | .883^*^ | 0.020 | 0.562 | 0.246 | 0.503 | 0.309 | 0.721 | 0.106 |
| CCNB2 | 0.811 | 0.050 | 0.649 | 0.163 | 0.488 | 0.326 | 0.720 | 0.107 |
| P2RY14 | -.974^**^ | 0.001 | -0.760 | 0.080 | -.834^*^ | 0.039 | -.938^**^ | 0.006 |
| HIST1H1B | .875^*^ | 0.022 | 0.716 | 0.109 | 0.668 | 0.147 | .828^*^ | 0.042 |
| LOC100128727 | 0.554 | 0.254 | .938^**^ | 0.006 | .943^**^ | 0.005 | .873^*^ | 0.023 |
| MACROD2 | -.949^**^ | 0.004 | -.830^*^ | 0.041 | -.830^*^ | 0.041 | -.953^**^ | 0.003 |
| PYGB | .988^**^ | 0.000 | 0.688 | 0.131 | 0.719 | 0.107 | .880^*^ | 0.021 |
| MAMDC2 | -.969^**^ | 0.001 | -0.587 | 0.220 | -0.603 | 0.205 | -0.797 | 0.058 |
| FAM151B | .831^*^ | 0.040 | .812^*^ | 0.050 | .971^**^ | 0.001 | .945^**^ | 0.004 |
| VSTM2A | -.900^*^ | 0.014 | -0.648 | 0.164 | -.838^*^ | 0.037 | -.869^*^ | 0.025 |
| HIST1H3J | .907^*^ | 0.013 | 0.628 | 0.182 | 0.566 | 0.241 | 0.776 | 0.070 |
| WDR91 | -.918^**^ | 0.010 | -0.655 | 0.158 | -0.592 | 0.216 | -0.798 | 0.057 |
| S100A3 | .853^*^ | 0.031 | .832^*^ | 0.040 | .881^*^ | 0.020 | .932^**^ | 0.007 |
| TMEM174 | -0.680 | 0.137 | -.994^**^ | 0.000 | -.897^*^ | 0.015 | -.928^**^ | 0.008 |
| EHF | .905^*^ | 0.013 | 0.469 | 0.348 | 0.462 | 0.357 | 0.682 | 0.136 |
| AIMP2 | .965^**^ | 0.002 | .818^*^ | 0.047 | .838^*^ | 0.037 | .957^**^ | 0.003 |
| PLOD1 | .846^*^ | 0.034 | .876^*^ | 0.022 | 0.751 | 0.085 | .903^*^ | 0.014 |
| SCD5 | -.919^**^ | 0.010 | -0.585 | 0.223 | -0.590 | 0.218 | -0.772 | 0.072 |
| PDE1C | -.857^*^ | 0.029 | -0.740 | 0.093 | -.867^*^ | 0.025 | -.895^*^ | 0.016 |
| HIST1H2AL | .842^*^ | 0.036 | 0.741 | 0.092 | 0.635 | 0.176 | .814^*^ | 0.049 |
| WDHD1 | .962^**^ | 0.002 | 0.686 | 0.133 | 0.667 | 0.148 | .851^*^ | 0.032 |
| HIST1H3A | .845^*^ | 0.034 | 0.748 | 0.087 | 0.803 | 0.054 | .872^*^ | 0.023 |
| PTPLA | -.847^*^ | 0.033 | -0.599 | 0.209 | -0.607 | 0.201 | -0.754 | 0.083 |
| PAIP2B | -.853^*^ | 0.031 | -0.691 | 0.128 | -0.627 | 0.183 | -0.797 | 0.058 |
| TICRR | .957^**^ | 0.003 | 0.707 | 0.116 | 0.704 | 0.118 | .870^*^ | 0.024 |
| ADAMTSL1 | -.911^*^ | 0.011 | -.870^*^ | 0.024 | -.897^*^ | 0.015 | -.974^**^ | 0.001 |
| NPHS1 | -0.738 | 0.094 | -0.593 | 0.214 | -0.609 | 0.200 | -0.709 | 0.114 |
| PLP2 | .920^**^ | 0.009 | 0.675 | 0.141 | 0.577 | 0.230 | 0.802 | 0.055 |
| CCL18 | 0.755 | 0.083 | 0.526 | 0.284 | 0.488 | 0.326 | 0.652 | 0.161 |
| POLQ | 0.777 | 0.069 | 0.483 | 0.332 | 0.513 | 0.299 | 0.653 | 0.160 |
| SHCBP1 | .910^*^ | 0.012 | 0.724 | 0.104 | 0.731 | 0.099 | .866^*^ | 0.026 |
| QPCT | 0.652 | 0.160 | 0.726 | 0.103 | 0.628 | 0.182 | 0.731 | 0.099 |
| GPER1 | -.987^**^ | 0.000 | -0.700 | 0.122 | -0.701 | 0.121 | -.878^*^ | 0.021 |
| CDCA8 | .911^*^ | 0.011 | 0.638 | 0.173 | 0.592 | 0.216 | 0.789 | 0.062 |
| ANXA8L1 | 0.682 | 0.135 | .910^*^ | 0.012 | .950^**^ | 0.004 | .915^*^ | 0.010 |
| CDCA7 | .876^*^ | 0.022 | 0.737 | 0.095 | 0.798 | 0.057 | .879^*^ | 0.021 |
| CENPO | .947^**^ | 0.004 | 0.709 | 0.115 | 0.734 | 0.097 | .876^*^ | 0.022 |
| ADAMTSL1 | -.966^**^ | 0.002 | -0.788 | 0.062 | -0.760 | 0.079 | -.921^**^ | 0.009 |
| SH2D6 | -.900^*^ | 0.014 | -0.568 | 0.239 | -0.558 | 0.250 | -0.748 | 0.087 |
| PDILT | -.905^*^ | 0.013 | -0.810 | 0.051 | -.872^*^ | 0.024 | -.941^**^ | 0.005 |
| ACOT7 | .904^*^ | 0.013 | .858^*^ | 0.029 | 0.759 | 0.080 | .922^**^ | 0.009 |
| FABP3 | -0.547 | 0.262 | -0.371 | 0.470 | -0.278 | 0.594 | -0.444 | 0.378 |
| KIF20B | 0.766 | 0.076 | 0.594 | 0.214 | 0.654 | 0.159 | 0.736 | 0.096 |
| HIST1H4E | .941^**^ | 0.005 | 0.611 | 0.198 | 0.684 | 0.134 | .821^*^ | 0.045 |
| MND1 | .843^*^ | 0.035 | 0.366 | 0.475 | 0.449 | 0.372 | 0.616 | 0.193 |
| CTSF | -.966^**^ | 0.002 | -0.771 | 0.073 | -0.703 | 0.119 | -.896^*^ | 0.016 |
| PPA1 | .862^*^ | 0.027 | .838^*^ | 0.037 | .827^*^ | 0.043 | .920^**^ | 0.009 |
| PCDH11Y | -.854^*^ | 0.030 | -.859^*^ | 0.029 | -.950^**^ | 0.004 | -.965^**^ | 0.002 |
| HIST1H2AB | .973^**^ | 0.001 | 0.598 | 0.210 | 0.581 | 0.226 | 0.796 | 0.058 |
| HMGB3 | .969^**^ | 0.001 | 0.587 | 0.221 | 0.659 | 0.155 | .815^*^ | 0.048 |
| LCE2D | -0.720 | 0.107 | -0.671 | 0.145 | -0.602 | 0.206 | -0.729 | 0.100 |
| FERMT1 | .935^**^ | 0.006 | 0.737 | 0.094 | 0.671 | 0.144 | .861^*^ | 0.028 |
| ESRRG | -0.753 | 0.084 | -.894^*^ | 0.016 | -.837^*^ | 0.038 | -.901^*^ | 0.014 |
| ARMC3 | -.873^*^ | 0.023 | -0.758 | 0.081 | -0.728 | 0.101 | -.863^*^ | 0.027 |
| GPRIN1 | .837^*^ | 0.038 | 0.743 | 0.091 | 0.634 | 0.176 | .812^*^ | 0.050 |
| LEPR | -0.797 | 0.058 | -0.606 | 0.202 | -0.512 | 0.299 | -0.706 | 0.117 |
| GUCA1B | .889^*^ | 0.018 | 0.488 | 0.326 | 0.635 | 0.175 | 0.740 | 0.093 |
| LOC401433 | .882^*^ | 0.020 | 0.458 | 0.361 | 0.521 | 0.289 | 0.688 | 0.131 |
| WDR1 | 0.755 | 0.083 | 0.649 | 0.163 | 0.636 | 0.175 | 0.746 | 0.089 |
| SCUBE2 | -.952^**^ | 0.003 | -0.619 | 0.190 | -0.670 | 0.145 | -.824^*^ | 0.044 |
| CEACAM1 | .877^*^ | 0.022 | .843^*^ | 0.035 | .903^*^ | 0.014 | .953^**^ | 0.003 |
| PRPH2 | -0.754 | 0.084 | -0.155 | 0.769 | -0.202 | 0.701 | -0.422 | 0.405 |
| CENPE | .847^*^ | 0.033 | 0.716 | 0.109 | 0.607 | 0.201 | 0.798 | 0.057 |
| GNMT | -.824^*^ | 0.044 | -.832^*^ | 0.040 | -.843^*^ | 0.035 | -.908^*^ | 0.012 |
| SGOL1 | .898^*^ | 0.015 | 0.624 | 0.185 | 0.675 | 0.141 | 0.806 | 0.053 |
| GIF | -.910^*^ | 0.012 | -.864^*^ | 0.026 | -.835^*^ | 0.038 | -.952^**^ | 0.003 |
| LOC645195 | -.929^**^ | 0.007 | -0.773 | 0.071 | -0.807 | 0.052 | -.916^*^ | 0.010 |
| CYP26B1 | 0.696 | 0.125 | .921^**^ | 0.009 | .972^**^ | 0.001 | .932^**^ | 0.007 |
| INPP5F | .812^*^ | 0.049 | 0.672 | 0.144 | 0.704 | 0.119 | 0.799 | 0.057 |
| C19orf73 | 0.780 | 0.067 | 0.647 | 0.165 | 0.795 | 0.059 | 0.807 | 0.052 |
| LMTK2 | .862^*^ | 0.027 | 0.736 | 0.096 | .887^*^ | 0.018 | .902^*^ | 0.014 |
| DCAF8L2 | -0.697 | 0.124 | -0.205 | 0.697 | -0.177 | 0.737 | -0.409 | 0.420 |
| SEMA5B | 0.755 | 0.082 | 0.733 | 0.097 | 0.697 | 0.124 | 0.797 | 0.058 |
| CA12 | -0.649 | 0.163 | -0.143 | 0.787 | -0.113 | 0.831 | -0.347 | 0.501 |
| PRKDC | .956^**^ | 0.003 | 0.771 | 0.073 | .868^*^ | 0.025 | .946^**^ | 0.004 |
| RAD18 | .942^**^ | 0.005 | 0.742 | 0.091 | 0.672 | 0.143 | .866^*^ | 0.026 |
| GTSE1 | .853^*^ | 0.031 | 0.743 | 0.091 | 0.635 | 0.176 | .819^*^ | 0.046 |
| CYP2J2 | .966^**^ | 0.002 | 0.637 | 0.174 | 0.654 | 0.159 | .831^*^ | 0.041 |
| HIST1H3D | .887^*^ | 0.018 | 0.563 | 0.244 | 0.520 | 0.290 | 0.729 | 0.100 |
| PGA3 | -.934^**^ | 0.006 | -.844^*^ | 0.035 | -.864^*^ | 0.026 | -.963^**^ | 0.002 |
| KCNQ1 | -0.642 | 0.169 | -0.774 | 0.071 | -0.620 | 0.189 | -0.742 | 0.091 |
| PRR22 | 0.667 | 0.148 | 0.043 | 0.936 | 0.185 | 0.726 | 0.340 | 0.509 |
| HIST1H3E | .877^*^ | 0.022 | 0.686 | 0.132 | 0.605 | 0.203 | 0.798 | 0.057 |
| MKI67 | .955^**^ | 0.003 | 0.707 | 0.117 | 0.687 | 0.131 | .863^*^ | 0.027 |
| C1QTNF9 | -.942^**^ | 0.005 | -.856^*^ | 0.030 | -.869^*^ | 0.025 | -.972^**^ | 0.001 |
| TMEM229A | -0.681 | 0.136 | -0.807 | 0.053 | -0.675 | 0.141 | -0.788 | 0.063 |
| MCOLN3 | -.947^**^ | 0.004 | -0.622 | 0.187 | -0.674 | 0.142 | -.824^*^ | 0.044 |
| ZWINT | .939^**^ | 0.006 | 0.677 | 0.140 | 0.594 | 0.214 | .815^*^ | 0.048 |
| SLC16A13 | .830^*^ | 0.041 | 0.783 | 0.065 | 0.646 | 0.166 | .828^*^ | 0.042 |
| HIST1H2BJ | .921^**^ | 0.009 | 0.590 | 0.218 | 0.589 | 0.219 | 0.774 | 0.071 |
| C11orf40 | -.895^*^ | 0.016 | -0.729 | 0.100 | -0.793 | 0.060 | -.882^*^ | 0.020 |
| CLN6 | .962^**^ | 0.002 | 0.560 | 0.248 | 0.544 | 0.264 | 0.765 | 0.076 |
| CKAP2 | .891^*^ | 0.017 | 0.711 | 0.113 | 0.725 | 0.103 | .852^*^ | 0.031 |
| GPSM2 | .868^*^ | 0.025 | 0.794 | 0.060 | 0.665 | 0.150 | .853^*^ | 0.031 |
| BCL2L11 | -.845^*^ | 0.034 | -0.746 | 0.089 | -0.795 | 0.059 | -.869^*^ | 0.025 |
| RHBG | .959^**^ | 0.003 | 0.501 | 0.311 | 0.618 | 0.191 | 0.766 | 0.076 |
| GPER1 | -.882^*^ | 0.020 | -0.785 | 0.065 | -.902^*^ | 0.014 | -.933^**^ | 0.007 |
| HYDIN | -0.689 | 0.130 | -0.379 | 0.459 | -0.394 | 0.439 | -0.541 | 0.267 |
| ST8SIA2 | .843^*^ | 0.035 | 0.465 | 0.353 | 0.556 | 0.252 | 0.686 | 0.132 |
| LIFR | -.859^*^ | 0.028 | -.830^*^ | 0.041 | -0.776 | 0.070 | -.899^*^ | 0.015 |
| CENPI | .909^*^ | 0.012 | 0.645 | 0.167 | 0.630 | 0.180 | 0.803 | 0.054 |
| C5orf34 | .844^*^ | 0.035 | .961^**^ | 0.002 | .941^**^ | 0.005 | .995^**^ | 0.000 |
| MT1M | -0.748 | 0.088 | -.825^*^ | 0.043 | -.884^*^ | 0.020 | -.888^*^ | 0.018 |
| PAPPA2 | -.847^*^ | 0.033 | -.817^*^ | 0.047 | -.886^*^ | 0.019 | -.925^**^ | 0.008 |
| MYBL2 | .928^**^ | 0.008 | .848^*^ | 0.033 | .897^*^ | 0.015 | .972^**^ | 0.001 |
| CHEK2 | .885^*^ | 0.019 | .822^*^ | 0.045 | 0.761 | 0.079 | .902^*^ | 0.014 |
| SLC27A2 | .895^*^ | 0.016 | 0.694 | 0.126 | 0.810 | 0.050 | .875^*^ | 0.023 |
| SLC1A2 | -.964^**^ | 0.002 | -0.679 | 0.138 | -0.740 | 0.093 | -.874^*^ | 0.023 |
| NRXN1 | -.933^**^ | 0.007 | -0.750 | 0.086 | -.883^*^ | 0.020 | -.934^**^ | 0.006 |
| CPEB2 | -.937^**^ | 0.006 | -0.795 | 0.059 | -0.723 | 0.104 | -.900^*^ | 0.014 |
| PTGDR2 | -.918^**^ | 0.010 | -0.748 | 0.087 | -.820^*^ | 0.046 | -.907^*^ | 0.013 |
| DDIAS | 0.759 | 0.080 | 0.252 | 0.630 | 0.307 | 0.553 | 0.494 | 0.319 |
| LDHA | 0.705 | 0.118 | 0.681 | 0.136 | 0.617 | 0.192 | 0.732 | 0.098 |
| ESCO2 | .953^**^ | 0.003 | 0.716 | 0.110 | 0.692 | 0.128 | .867^*^ | 0.025 |
| GJB2 | 0.797 | 0.058 | .840^*^ | 0.036 | .879^*^ | 0.021 | .912^*^ | 0.011 |
| ARHGAP11A | .888^*^ | 0.018 | .925^**^ | 0.008 | .817^*^ | 0.047 | .959^**^ | 0.002 |
| PCDHA11 | -.850^*^ | 0.032 | -0.342 | 0.507 | -0.478 | 0.337 | -0.619 | 0.190 |
| S100A16 | 0.782 | 0.066 | 0.658 | 0.155 | 0.523 | 0.287 | 0.723 | 0.104 |
| TNFRSF11A | .996^**^ | 0.000 | 0.641 | 0.170 | 0.702 | 0.120 | .860^*^ | 0.028 |
| XYLT2 | -.932^**^ | 0.007 | -0.783 | 0.066 | -0.750 | 0.086 | -.902^*^ | 0.014 |
| CHRNA5 | .949^**^ | 0.004 | 0.699 | 0.123 | 0.697 | 0.124 | .861^*^ | 0.028 |
| ADAM7 | .985^**^ | 0.000 | 0.727 | 0.102 | 0.698 | 0.123 | .886^*^ | 0.019 |
| RERGL | -.954^**^ | 0.003 | -.830^*^ | 0.041 | -0.796 | 0.058 | -.944^**^ | 0.005 |
| CCNF | 0.805 | 0.053 | 0.654 | 0.159 | 0.621 | 0.188 | 0.762 | 0.078 |
| GAMT | -.967^**^ | 0.002 | -0.756 | 0.082 | -0.747 | 0.088 | -.905^*^ | 0.013 |
| L1CAM | -.913^*^ | 0.011 | -0.511 | 0.301 | -0.700 | 0.122 | -0.778 | 0.069 |
| CELA3A | -0.463 | 0.355 | -.881^*^ | 0.020 | -0.754 | 0.083 | -0.755 | 0.083 |
| WEE1 | -.889^*^ | 0.018 | -.819^*^ | 0.046 | -0.765 | 0.077 | -.903^*^ | 0.014 |
| DNER | -.831^*^ | 0.041 | -0.755 | 0.082 | -0.708 | 0.115 | -.839^*^ | 0.037 |
| KIF11 | .961^**^ | 0.002 | 0.630 | 0.180 | 0.641 | 0.171 | .822^*^ | 0.045 |
| RGN | -.889^*^ | 0.018 | -0.624 | 0.186 | -0.567 | 0.240 | -0.767 | 0.075 |
| CAB39L | -.969^**^ | 0.001 | -0.603 | 0.205 | -0.622 | 0.187 | -0.809 | 0.051 |
| HIST1H2AC | .887^*^ | 0.019 | 0.511 | 0.300 | 0.540 | 0.269 | 0.716 | 0.110 |
| PM20D1 | -.847^*^ | 0.033 | -0.635 | 0.176 | -0.653 | 0.160 | -0.783 | 0.066 |
| DBT | -0.619 | 0.190 | -0.651 | 0.161 | -0.577 | 0.230 | -0.674 | 0.142 |
| SCD5 | -.889^*^ | 0.018 | -0.614 | 0.195 | -0.694 | 0.126 | -0.805 | 0.053 |
| CD276 | .832^*^ | 0.040 | .930^**^ | 0.007 | .939^**^ | 0.005 | .978^**^ | 0.001 |
| CCRL2 | .840^*^ | 0.037 | 0.500 | 0.313 | 0.511 | 0.300 | 0.684 | 0.134 |
| NEDD4L | -0.577 | 0.231 | -0.752 | 0.085 | -0.644 | 0.168 | -0.716 | 0.110 |
| DRD5 | -0.551 | 0.257 | -0.775 | 0.071 | -0.640 | 0.171 | -0.712 | 0.112 |
| PLEKHM3 | -.876^*^ | 0.022 | -0.481 | 0.334 | -0.544 | 0.264 | -0.702 | 0.120 |
| S100A12 | 0.769 | 0.074 | 0.702 | 0.120 | 0.561 | 0.246 | 0.747 | 0.088 |
| ASB11 | -0.742 | 0.091 | -.820^*^ | 0.046 | -.831^*^ | 0.040 | -.867^*^ | 0.025 |
| SSC5D | -.873^*^ | 0.023 | -0.727 | 0.102 | -0.694 | 0.126 | -.840^*^ | 0.036 |
| SYNE1 | -.854^*^ | 0.031 | -.914^*^ | 0.011 | -.828^*^ | 0.042 | -.945^**^ | 0.004 |
| MKI67 | .876^*^ | 0.022 | .836^*^ | 0.038 | 0.763 | 0.078 | .904^*^ | 0.013 |
| MARVELD3 | .930^**^ | 0.007 | 0.764 | 0.077 | .886^*^ | 0.019 | .939^**^ | 0.006 |
| LRRC3 | 0.768 | 0.075 | .942^**^ | 0.005 | .957^**^ | 0.003 | .964^**^ | 0.002 |
| PLAU | .874^*^ | 0.023 | 0.767 | 0.075 | 0.757 | 0.081 | .876^*^ | 0.022 |
| KRT14 | 0.697 | 0.124 | .963^**^ | 0.002 | .927^**^ | 0.008 | .933^**^ | 0.007 |
| CENPF | .907^*^ | 0.013 | 0.710 | 0.114 | 0.666 | 0.149 | .839^*^ | 0.037 |
| CADM2 | -.829^*^ | 0.041 | -.969^**^ | 0.001 | -.881^*^ | 0.021 | -.973^**^ | 0.001 |
| ZWINT | .878^*^ | 0.021 | 0.691 | 0.128 | 0.688 | 0.131 | .827^*^ | 0.042 |
| CHEK2 | .875^*^ | 0.022 | .841^*^ | 0.036 | .884^*^ | 0.019 | .945^**^ | 0.004 |
| PCNA | .876^*^ | 0.022 | 0.790 | 0.061 | .821^*^ | 0.045 | .906^*^ | 0.013 |
| CAB39L | -.958^**^ | 0.003 | -0.530 | 0.279 | -0.545 | 0.264 | -0.753 | 0.084 |
| PPA1 | 0.760 | 0.080 | 0.730 | 0.100 | 0.695 | 0.125 | 0.797 | 0.058 |
| SLC23A3 | 0.580 | 0.228 | 0.492 | 0.322 | 0.297 | 0.568 | 0.508 | 0.304 |
| AMMECR1 | .937^**^ | 0.006 | 0.757 | 0.081 | 0.724 | 0.104 | .886^*^ | 0.019 |
| GDNF | -.923^**^ | 0.009 | -0.497 | 0.316 | -0.600 | 0.208 | -0.744 | 0.090 |
| HIST2H2BF | .900^*^ | 0.014 | 0.566 | 0.242 | 0.545 | 0.264 | 0.743 | 0.091 |
| PDE1C | -.923^**^ | 0.009 | -0.768 | 0.074 | -.842^*^ | 0.036 | -.923^**^ | 0.009 |
| SGOL2 | 0.746 | 0.089 | 0.503 | 0.309 | 0.466 | 0.352 | 0.633 | 0.177 |
| THSD4 | -.878^*^ | 0.021 | -.830^*^ | 0.041 | -0.720 | 0.107 | -.888^*^ | 0.018 |
| FIGN | -.918^**^ | 0.010 | -0.468 | 0.350 | -0.475 | 0.341 | -0.691 | 0.128 |
| PSG8 | .846^*^ | 0.034 | .941^**^ | 0.005 | .819^*^ | 0.046 | .949^**^ | 0.004 |
| ARHGAP11A | .940^**^ | 0.005 | 0.682 | 0.136 | 0.720 | 0.106 | .859^*^ | 0.028 |
| RAD51D | .920^**^ | 0.009 | .838^*^ | 0.037 | .913^*^ | 0.011 | .971^**^ | 0.001 |
| SLC14A1 | -0.751 | 0.085 | -.948^**^ | 0.004 | -0.791 | 0.061 | -.905^*^ | 0.013 |
| P2RX6 | -0.781 | 0.066 | -0.643 | 0.169 | -0.508 | 0.303 | -0.712 | 0.112 |
| FGA | -.890^*^ | 0.017 | -.838^*^ | 0.037 | -0.756 | 0.082 | -.908^*^ | 0.012 |
| STIL | .834^*^ | 0.039 | 0.444 | 0.378 | 0.414 | 0.415 | 0.629 | 0.181 |
| LDHA | .842^*^ | 0.035 | 0.742 | 0.092 | 0.710 | 0.114 | .839^*^ | 0.037 |
| NRXN1 | -.945^**^ | 0.004 | -0.666 | 0.149 | -0.720 | 0.106 | -.855^*^ | 0.030 |
| STXBP5L | -.969^**^ | 0.001 | -0.669 | 0.146 | -0.750 | 0.086 | -.875^*^ | 0.022 |
| MTHFD1L | .957^**^ | 0.003 | 0.720 | 0.107 | 0.778 | 0.068 | .898^*^ | 0.015 |
| PGA3 | -.861^*^ | 0.028 | -0.802 | 0.055 | -.822^*^ | 0.045 | -.905^*^ | 0.013 |
| PLK1 | .940^**^ | 0.005 | 0.726 | 0.102 | 0.698 | 0.123 | .868^*^ | 0.025 |
| SYNGR1 | -0.651 | 0.161 | -.840^*^ | 0.036 | -0.781 | 0.067 | -.822^*^ | 0.045 |
| KBTBD12 | -0.807 | 0.052 | -0.731 | 0.099 | -0.684 | 0.134 | -.813^*^ | 0.049 |
| ESCO2 | .857^*^ | 0.029 | 0.664 | 0.150 | 0.623 | 0.186 | 0.788 | 0.063 |
| SEMA5B | 0.787 | 0.063 | 0.657 | 0.156 | 0.673 | 0.143 | 0.774 | 0.071 |
| AQP4 | -.827^*^ | 0.042 | -0.770 | 0.073 | -0.694 | 0.126 | -.838^*^ | 0.037 |
| TRIM50 | -.828^*^ | 0.042 | -0.776 | 0.070 | -0.713 | 0.112 | -.846^*^ | 0.034 |
| GPER1 | -.835^*^ | 0.039 | -.817^*^ | 0.047 | -.850^*^ | 0.032 | -.909^*^ | 0.012 |
| C19orf81 | -0.755 | 0.083 | -0.659 | 0.154 | -0.733 | 0.098 | -0.781 | 0.067 |
| SLC14A2 | -.839^*^ | 0.037 | -0.631 | 0.179 | -0.688 | 0.130 | -0.790 | 0.062 |
| TOMM40 | .972^**^ | 0.001 | 0.647 | 0.165 | 0.757 | 0.082 | .870^*^ | 0.024 |
| HES6 | 0.777 | 0.069 | .866^*^ | 0.026 | .945^**^ | 0.005 | .935^**^ | 0.006 |
| PPA1 | 0.804 | 0.054 | 0.706 | 0.117 | 0.719 | 0.108 | .813^*^ | 0.049 |
| GDNF | -0.774 | 0.071 | -0.299 | 0.565 | -0.500 | 0.313 | -0.580 | 0.228 |
| SHANK2 | -0.628 | 0.182 | -0.559 | 0.249 | -0.388 | 0.447 | -0.581 | 0.226 |
| SYNGR1 | -0.698 | 0.123 | -.864^*^ | 0.027 | -0.776 | 0.070 | -.848^*^ | 0.033 |
| S100B | -.844^*^ | 0.035 | -.834^*^ | 0.039 | -.948^**^ | 0.004 | -.951^**^ | 0.004 |
| PLEKHS1 | .965^**^ | 0.002 | 0.554 | 0.254 | 0.619 | 0.190 | 0.789 | 0.062 |
| Note: ^*^, significance at *P*<0.05; ^**^, significance at *P*<0.01. | | | | | | | | |

| Table S5. The characteristics of three GC cases to be detected | | | |
| --- | --- | --- | --- |
| Variables | GC-1 | GC-2 | GC-3 |
| Gender | male | male | male |
| Age | 69 | 66 | 64 |
| Tumor cite | corpus, angle and antrum | antrum | antrum |
| Macroscopic type | ulcerative/invasive type | ulcerative/invasive type | ulcerative/invasive type |
| Histological type | middle/low differentiation | low differentiation | middle differentiation |
| Lymphatic metastasis | negative | positive | positive |
| Note: GC, gastric cancer. | | | |

| Table S6. The mRNA expression levels of selected differentially co-expressed genes in GC cell lines based on CCLE | | | | | | | | | | | | | | | | | | | | | | | | | | | | | | |
| --- | --- | --- | --- | --- | --- | --- | --- | --- | --- | --- | --- | --- | --- | --- | --- | --- | --- | --- | --- | --- | --- | --- | --- | --- | --- | --- | --- | --- | --- | --- |
| GC cell lines | Differentiation | CEACAM1 | ATP10B | FUT6 | PSG8 | ARHGAP11A | C5orf34 | FAM151B | CHEK2 | AIMP2 | RAD51D | POF1B | PPA1 | S100A3 | PCNA | MYBL2 | CD276 | PGA3 | GIF | GPER1 | FGA | SYNE1 | GNMT | MACROD2 | PCDH11Y | C1QTNF9 | S100B | ADAMTSL1 | CADM2 | PAPPA2 |
| RERFGC1B | Unknown | 1.63 | -3.29 | -3.79 | -2.26 | 3.96 | 0.94 | -0.74 | 3.04 | 4.64 | 3.30 | 4.75 | 4.73 | -0.53 | 6.44 | 8.61 | 4.73 | -7.48 | -13.00 | 1.02 | 1.55 | 0.25 | -3.53 | -1.62 | -13.00 | -13.00 | -3.88 | -6.04 | -3.00 | -5.25 |
| HS-746T | Low | 2.02 | -8.21 | -13.00 | -3.23 | 4.64 | 2.36 | -2.23 | 2.45 | 3.93 | 2.46 | -1.15 | 7.31 | 2.44 | 6.72 | 0.25 | 4.21 | -13.00 | -13.00 | 0.00 | -6.49 | 1.27 | -1.50 | -7.43 | -5.51 | -13.00 | -6.04 | -4.43 | -4.26 | -2.01 |
| LMSU | Unknown | -2.39 | -6.61 | -13.00 | -6.46 | 5.01 | 1.68 | -2.41 | 1.73 | 5.18 | 1.92 | -4.03 | 6.27 | 2.35 | 6.84 | 5.96 | 3.31 | -13.00 | -13.00 | -3.03 | -4.40 | -0.48 | -1.03 | -6.17 | -13.00 | -13.00 | -5.61 | 3.33 | -7.40 | 0.84 |
| ECC10 | Low | 3.42 | -7.55 | -4.15 | -6.81 | 4.60 | 1.92 | -3.12 | 2.19 | 4.85 | 3.21 | -4.44 | 5.42 | 2.52 | 8.17 | 5.93 | 4.92 | -6.11 | -2.44 | -2.06 | -6.09 | -0.05 | -1.44 | -0.29 | -13.00 | -13.00 | -2.64 | -2.96 | -8.75 | 0.77 |
| TGBC11TKB | Unknown | 2.18 | -4.04 | -1.77 | -13.00 | 4.32 | 1.48 | -1.05 | 3.17 | 4.48 | 2.62 | 5.28 | 6.37 | -3.18 | 6.52 | 3.41 | 3.81 | -13.00 | -13.00 | 0.74 | -13.00 | -4.77 | -2.37 | -2.91 | -9.25 | -13.00 | -1.19 | -3.59 | -9.15 | -4.11 |
| SNU-520 | Low | -2.32 | 1.75 | 1.39 | -6.39 | 4.80 | 0.38 | 0.05 | 2.55 | 4.82 | 2.73 | 5.98 | 7.66 | -0.41 | 7.92 | 4.63 | 2.64 | -7.68 | -13.00 | -2.68 | -6.99 | -7.17 | -5.54 | -2.06 | -13.00 | -13.00 | -1.70 | -7.32 | -9.32 | -6.45 |
| GSS | Unknown | -4.13 | -9.02 | -8.79 | -4.82 | 4.20 | 1.01 | -2.34 | 3.07 | 6.70 | 2.93 | -1.26 | 6.86 | -2.13 | 6.66 | 5.79 | 3.82 | -8.58 | -13.00 | -3.75 | -6.56 | -0.95 | -2.11 | -1.85 | -13.00 | -13.00 | -4.62 | -7.80 | -6.90 | -7.03 |
| SNU-620 | Low | 5.52 | 3.55 | -0.47 | -6.53 | 3.76 | 1.21 | -3.07 | 2.59 | 4.34 | 3.19 | 6.24 | 7.03 | -3.17 | 7.74 | 3.90 | 3.23 | -13.00 | -5.85 | 0.52 | -7.13 | -7.27 | -2.99 | -2.22 | -13.00 | -13.00 | -1.29 | -8.05 | -7.66 | -9.01 |
| ECC12 | Low | 1.74 | -4.59 | -5.40 | -13.00 | 4.41 | 1.99 | -0.84 | 3.17 | 4.63 | 2.97 | -3.11 | 5.95 | -2.17 | 7.64 | 7.15 | 3.33 | -13.00 | -13.00 | 2.22 | -7.22 | 0.27 | -1.41 | -1.19 | -6.34 | -13.00 | -2.50 | 2.12 | 3.72 | -4.85 |
| GSU | Undifferentiated | 2.42 | 3.00 | 2.61 | -6.20 | 4.33 | 0.23 | -2.02 | 2.03 | 5.24 | 2.21 | 6.22 | 7.36 | -2.36 | 6.75 | 4.52 | 0.09 | -13.00 | -13.00 | 1.59 | -5.22 | -9.04 | -3.19 | -2.72 | -13.00 | -13.00 | -7.36 | -7.99 | -13.00 | -8.69 |
| FU97 | Undifferentiated | 4.38 | -13.00 | -0.58 | -13.00 | 3.98 | 2.69 | -2.01 | 4.30 | 4.46 | 1.57 | -5.73 | 6.69 | -4.39 | 7.73 | 7.26 | 6.16 | -13.00 | -13.00 | 2.13 | 6.20 | -1.39 | -0.09 | -3.63 | -13.00 | -13.00 | -13.00 | -9.53 | -8.78 | -5.45 |
| GCIY | Undifferentiated | 1.35 | -5.74 | 0.04 | -13.00 | 4.87 | 2.13 | -1.76 | 2.50 | 3.57 | 2.97 | 3.46 | 5.04 | -1.66 | 5.52 | 5.26 | 3.81 | -13.00 | -4.13 | 1.51 | -0.93 | 3.19 | -1.97 | 3.30 | -13.00 | -13.00 | -4.96 | -1.51 | -1.59 | -5.29 |
| SH-10-TC | Unknown | 1.38 | -9.69 | -7.46 | -6.37 | 5.36 | 1.78 | -0.37 | 3.02 | 4.81 | 3.36 | -4.33 | 6.25 | 1.38 | 7.66 | 6.49 | 5.09 | -13.00 | -7.28 | -0.10 | -6.56 | -4.20 | -1.59 | -6.17 | -13.00 | -13.00 | -5.52 | -3.97 | -8.89 | -9.02 |
| MKN1 | High | 0.58 | -8.13 | -13.00 | -7.39 | 3.95 | 3.12 | -2.06 | 2.48 | 4.44 | 1.07 | -2.54 | 5.70 | 4.46 | 7.05 | 7.31 | 5.63 | -7.69 | -13.00 | -0.03 | -2.48 | 0.35 | -1.19 | -3.53 | -13.00 | -13.00 | -13.00 | -6.25 | -4.37 | -5.13 |
| MKN74 | Middle | -0.26 | -3.89 | -3.36 | -3.85 | 4.17 | 1.73 | -0.10 | 1.88 | 5.38 | 1.97 | -1.56 | 6.68 | -1.54 | 5.57 | 3.46 | 3.07 | -6.61 | -7.63 | -0.28 | -5.59 | -5.91 | -1.94 | -2.12 | -10.35 | -13.00 | -13.00 | -7.09 | -9.25 | -5.52 |
| KE-39 | Low | 3.56 | -3.89 | -4.23 | -13.00 | 4.09 | 1.47 | -3.33 | 2.98 | 4.21 | 3.13 | 3.95 | 4.59 | -3.27 | 8.81 | 5.70 | 3.67 | -7.61 | -13.00 | 0.12 | -8.91 | -0.82 | -2.88 | -2.38 | -13.00 | -13.00 | -5.87 | -13.00 | -8.25 | -9.37 |
| HGC-27 | Undifferentiated | -5.15 | -9.09 | -6.28 | -13.00 | 4.47 | 2.56 | -1.81 | 3.16 | 6.76 | 2.73 | -4.34 | 6.92 | -0.66 | 7.89 | 6.27 | 3.92 | -13.00 | -13.00 | 3.98 | -7.95 | -5.19 | -2.05 | -3.65 | -10.39 | -13.00 | -6.50 | 0.19 | 1.78 | -13.00 |
| HUG1N | Unknown | 1.46 | 2.30 | 3.04 | -13.00 | 4.23 | 1.48 | -2.04 | 2.83 | 5.31 | 1.73 | 5.22 | 7.49 | -1.65 | 8.09 | 6.70 | 2.69 | -13.00 | -13.00 | -3.11 | -8.02 | -6.94 | -1.45 | -4.96 | -13.00 | -13.00 | -3.40 | -5.77 | -13.00 | -7.32 |
| NUGC-4 | Low | 1.84 | -2.13 | 3.48 | -13.00 | 3.08 | 1.11 | -2.10 | 1.62 | 5.50 | 2.75 | 5.12 | 7.14 | -0.22 | 6.51 | 3.47 | 2.02 | -13.00 | -5.20 | -1.86 | -8.48 | -4.37 | -3.44 | 2.11 | -13.00 | -13.00 | -3.86 | -13.00 | -8.23 | -7.13 |
| SNU-16 | Low | 2.28 | 1.42 | 1.02 | -8.18 | 4.85 | 2.43 | -1.35 | 3.95 | 5.13 | 3.01 | 5.97 | 7.11 | -3.15 | 8.12 | 6.49 | 1.31 | -13.00 | -13.00 | -1.98 | -4.32 | -7.18 | -2.09 | -2.34 | -13.00 | -13.00 | -7.33 | -10.29 | -7.12 | -8.25 |
| NCI-N87 | High | 0.77 | -1.04 | -1.38 | -7.46 | 5.01 | 2.01 | -2.26 | 3.43 | 4.47 | 2.66 | 4.41 | 7.50 | -1.22 | 7.65 | 5.34 | 4.48 | -8.34 | -13.00 | 2.27 | -5.06 | -1.50 | -1.74 | -2.16 | -11.08 | -13.00 | -3.80 | -8.83 | -9.98 | -6.07 |
| OCUM-1 | Low | 4.71 | -0.01 | -4.26 | -6.14 | 3.87 | 1.14 | -1.55 | 2.86 | 4.12 | 2.02 | 5.31 | 6.99 | 0.46 | 7.58 | 4.86 | 5.11 | -13.00 | -13.00 | 0.24 | 1.37 | -6.98 | -1.09 | -6.56 | -13.00 | -13.00 | -4.29 | -6.79 | -5.62 | -8.21 |
| SNU-5 | Low | 4.63 | 2.58 | -13.00 | -13.00 | 4.69 | 2.08 | -1.87 | 2.78 | 5.64 | 2.60 | 3.55 | 7.29 | 2.28 | 7.29 | 6.14 | 3.23 | -13.00 | -13.00 | -1.40 | -8.08 | -3.28 | -5.05 | -7.60 | -13.00 | -13.00 | -1.46 | -7.00 | -13.00 | -8.54 |
| SNU-601 | Low | 4.08 | -3.89 | 1.59 | -7.35 | 4.72 | 1.02 | -2.16 | 3.53 | 4.91 | 2.42 | 3.43 | 7.37 | -2.73 | 7.37 | 6.80 | 3.96 | -13.00 | -6.67 | 2.18 | -7.95 | -7.04 | -2.33 | -0.54 | -13.00 | -13.00 | -1.39 | -6.45 | -13.00 | -8.09 |
| SNU-668 | Low | -1.90 | -7.45 | -13.00 | -6.29 | 3.57 | 2.30 | -1.66 | 2.31 | 4.78 | 1.36 | -5.60 | 7.33 | 2.17 | 7.84 | 5.94 | 3.67 | -13.00 | -13.00 | 4.18 | -7.90 | -7.85 | -3.28 | -8.09 | -13.00 | -13.00 | -0.99 | -10.40 | -9.23 | -8.36 |
| NCCSTCK140 | Unknown | 3.47 | -4.04 | -0.70 | -4.08 | 2.43 | 1.25 | -0.94 | 2.10 | 4.50 | 1.68 | 3.33 | 4.85 | -1.36 | 6.70 | 4.52 | 5.42 | -13.00 | -13.00 | -1.92 | -3.87 | -2.49 | -2.30 | -2.38 | -9.70 | -13.00 | -13.00 | -4.25 | -0.37 | -4.34 |
| SNU-719 | Middle | 4.65 | 1.62 | -5.50 | -13.00 | 3.32 | 1.62 | -2.57 | 2.78 | 4.54 | 2.94 | 4.88 | 6.87 | -1.30 | 7.99 | 4.94 | 3.88 | -13.00 | -7.12 | -3.68 | -4.81 | -2.13 | -1.78 | -2.22 | -13.00 | -13.00 | -1.74 | -7.90 | -8.15 | -5.11 |
| SNU-216 | Middle | -0.12 | -9.07 | -3.29 | 6.38 | 3.73 | 1.23 | -0.90 | 1.82 | 3.97 | 1.82 | 1.77 | 6.76 | -1.89 | 7.88 | 5.90 | 2.80 | -7.64 | -13.00 | -5.02 | -7.94 | -6.25 | -1.74 | -2.18 | -13.00 | -13.00 | -1.82 | -4.44 | -7.69 | -7.82 |
| NUGC-2 | Low | 2.02 | -7.60 | -1.04 | -2.48 | 3.34 | 2.50 | -1.90 | 2.96 | 5.43 | 1.55 | 0.54 | 5.71 | -1.61 | 7.32 | 7.74 | 4.90 | -13.00 | -13.00 | -2.19 | -4.15 | -2.35 | -2.22 | -3.24 | -13.00 | -13.00 | -13.00 | -6.97 | -9.39 | -2.06 |
| SNU-1 | Low | -2.66 | -9.25 | -9.02 | -5.19 | 5.23 | 3.02 | -0.45 | 3.02 | 4.05 | 2.43 | -4.62 | 6.48 | 0.80 | 8.35 | 6.41 | 4.07 | -13.00 | -13.00 | -5.08 | -6.11 | -6.84 | -0.54 | -1.10 | -13.00 | -13.00 | 0.54 | -13.00 | -13.00 | -7.99 |
| AGS | Middle | 2.42 | -2.42 | -13.00 | -2.96 | 4.74 | 1.24 | -1.94 | 2.66 | 4.29 | 2.34 | 4.87 | 6.83 | -0.95 | 8.11 | 7.44 | 4.85 | -6.93 | -4.63 | 0.49 | 2.80 | -0.90 | -2.33 | -6.75 | -13.00 | -13.00 | -5.78 | -7.42 | -7.98 | -13.00 |
| KATOIII | Low | 6.22 | 1.77 | 0.32 | -6.46 | 5.15 | 1.91 | -4.30 | 3.70 | 5.25 | 3.47 | 5.31 | 7.13 | 1.02 | 7.97 | 5.37 | -4.73 | -8.35 | -13.00 | 0.57 | -6.65 | -4.67 | -1.62 | -3.13 | -13.00 | -13.00 | -6.20 | -7.35 | -8.99 | -7.53 |
| NUGC-3 | Low | 0.52 | -5.96 | -2.22 | -8.03 | 4.57 | 2.83 | -1.85 | 3.61 | 5.02 | 1.82 | -3.24 | 6.50 | 1.39 | 8.29 | 5.78 | 3.88 | -13.00 | -13.00 | -4.50 | -13.00 | -3.44 | -1.38 | -2.80 | -13.00 | -13.00 | -3.48 | -9.14 | -8.39 | -5.93 |
| IM95 | Unknown | 1.72 | 3.47 | 2.37 | -13.00 | 4.57 | 1.99 | -0.91 | 3.64 | 5.53 | 2.73 | 4.44 | 6.74 | -2.10 | 7.70 | 6.00 | 3.00 | -13.00 | -7.52 | -5.30 | -4.99 | -7.84 | -2.65 | 2.04 | -10.24 | -13.00 | -7.35 | -10.30 | -13.00 | -6.26 |
| MKN7 | High | 1.46 | -5.59 | -2.39 | -0.34 | 3.53 | 2.67 | -3.68 | 2.95 | 5.11 | 1.12 | -5.40 | 6.04 | 1.69 | 7.64 | 6.11 | 4.45 | -13.00 | -13.00 | -4.41 | -5.74 | -3.58 | -1.93 | -2.74 | -9.98 | -13.00 | -13.00 | -3.32 | -8.88 | -13.00 |
| MKN45 | Low | 5.00 | 0.55 | -7.85 | -13.00 | 5.08 | 1.97 | -2.62 | 2.96 | 5.13 | 3.03 | 6.06 | 7.92 | -0.07 | 7.63 | 7.22 | 3.91 | -8.64 | -13.00 | -1.48 | -5.24 | -0.04 | -3.04 | -4.38 | -13.00 | -13.00 | -13.00 | -8.13 | -5.58 | -10.41 |
| 23132/87 | Middle | 3.11 | 0.07 | 3.30 | -13.00 | 4.71 | 1.98 | 0.17 | 3.53 | 4.53 | 1.73 | 4.58 | 6.59 | -2.59 | 6.97 | 5.45 | -0.34 | -13.00 | -13.00 | -4.22 | -0.79 | -3.90 | -2.40 | -2.87 | -6.66 | -13.00 | -6.10 | -13.00 | -4.52 | -4.06 |
| Note: GC, gastric cancer; CCLE, Cancer Cell Line Encyclopedia. | | | | | | | | | | | | | | | | | | | | | | | | | | | | | | |
